# Supported Gold Nanoparticle-Catalyzed Selective Reduction of Multifunctional, Aromatic Nitro Precursors into Amines and Synthesis of 3,4-Dihydroquinoxalin-2-Ones

**DOI:** 10.3390/molecules27144395

**Published:** 2022-07-08

**Authors:** Domna Iordanidou, Michael G. Kallitsakis, Marina A. Tzani, Dimitris I. Ioannou, Tryfon Zarganes-Tzitzikas, Constantinos G. Neochoritis, Alexander Dömling, Michael A. Terzidis, Ioannis N. Lykakis

**Affiliations:** 1 Department of Chemistry, Aristotle University of Thessaloniki, University Campus, 54124 Thessaloniki, Greece; domy-jo@hotmail.com (D.I.); kallitsos29@gmail.com (M.G.K.); marina_tzani@hotmail.com (M.A.T.); ioannou.dim1996@gmail.com (D.I.I.); 2 Department of Nutritional Sciences and Dietetics, International Hellenic University, Sindos Campus, 57400 Thessaloniki, Greece; 3 Alzheimer’s Research UK Oxford Drug Discovery Institute, University of Oxford, Oxford OX3 7FZ, UK; tryfon.zarganis-tzitzikas@ndm.ox.ac.uk; 4 Department of Chemistry, University of Crete, Voutes Heraklion, 70013 Heraklion, Greece; kneochor@uoc.gr; 5 Department of Pharmacy, University of Groningen, A. Deusinglaan 1, 9700 AV Groningen, The Netherlands; a.s.s.domling@rug.nl

**Keywords:** gold nanoparticles, reduction, heterocycles, catalysis, 3,4-dihydroquinoxalin-2-ones, nitro compounds, aromatic amines

## Abstract

The synthesis of 3,4-dihydroquinoxalin-2-ones via the selective reduction of aromatic, multifunctional nitro precursors catalyzed by supported gold nanoparticles is reported. The reaction proceeds through the in situ formation of the corresponding amines under heterogeneous transfer hydrogenation of the initial nitro compounds catalyzed by the commercially available Au/TiO_2_-Et_3_SiH catalytic system, followed by an intramolecular C-N transamidation upon treatment with silica acting as a mild acid. Under the present conditions, the Au/TiO_2_-TMDS system was also found to catalyze efficiently the present selective reduction process. Both transfer hydrogenation processes showed very good functional-group tolerance and were successfully applied to access more structurally demanding products bearing other reducible moieties such as chloro, aldehyde or methyl ketone. An easily scalable (up to 1 mmol), low catalyst loading (0.6 mol%) synthetic protocol was realized, providing access to this important scaffold. Under these mild catalytic conditions, the desired products were isolated in good to high yields and with a TON of 130. A library analysis was also performed to demonstrate the usefulness of our synthetic strategy and the physicochemical profile of the derivatives.

## 1. Introduction

The pharmaceutical, fine chemical and agrochemical industries utilize a wide range of amines and their derivatives that result from the reduction of the nitro group [1]. These fundamental organic chemistry transformations usually rely on transition metal-catalyzed hydrogenation [2]. However, its low chemoselectivity remains a continuous and persisting problem when other easily reducible groups are present. The role of noble metal nanoparticles such as gold (AuNPs) [3,4,5,6,7,8,9] and silver (AgNPs) [10,11,12,13,14], cobalt, iron, palladium, nickel and manganese-based compounds or oxides in such transformations have been extensively explored [15,16,17,18,19,20,21,22,23,24]. Hydrogenation as well as transfer hydrogenation processes have been used for the selective reduction of nitro compounds into the corresponding amines, but in most cases with significant drawbacks. The hydrogenation process requires high H_2_ pressure and temperature, with a very good applicability in monosubstituted aromatic nitro compounds. However, transfer hydrogenation is considered as a more chemoselective process, requiring milder conditions (e.g., ambient temperature and pressure) and works efficiently in the presence of several reducing agents, such as borohydrides, hydrosilanes, hydrazines, CO/H_2_O and HCOONH_4_ [7,8,9]. Thus, new catalytic and synthetic strategies are focused on the development of more efficient transfer hydrogenation processes that allow the selective reduction of aromatic amines in the presence of diverse functional moieties or protecting groups [25,26].

**Scheme 1:**
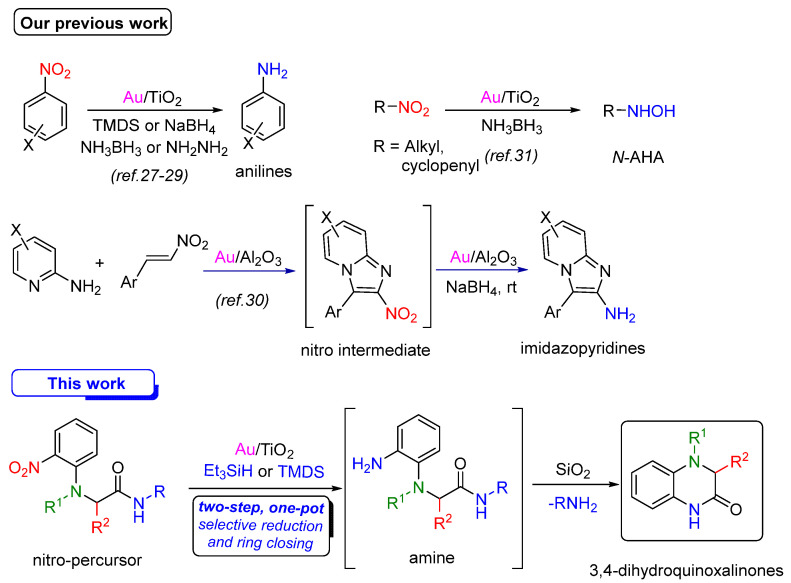
AuNP-catalyzed transfer hydrogenation processes towards the synthesis of *N*-substituted 3,4-dihydroquinoxalin-2-ones.

New synthetic methodologies for the synthesis of anilines and/or *N*-aryl hydroxylamines (*N*-AHA) using catalytic systems based on supported AuNPs and AgNPs on mesoporous titania, silica or alumina (Au/TiO_2_, Ag/TiO_2_, Ag/HMS and Au/Al_2_O_3_) under mild conditions were already reported by our research groups [27,28,29,30,31,32,33,34]. The application of mild conditions along with simple reducing agents, such as borohydrides, boranes, hydrosilanes and hydrazines, allowed the clean and rapid synthesis of the target compounds in high yields and with high chemoselectivity (Scheme 1). In this study, we explored the efficiency of the Au/TiO_2_-hydrosilane catalytic system in the highly diverse and demanding environment of multicomponent reactions (MCRs) in the absence of protecting groups [17,18]. The described protocol was then applied to the synthesis of aromatic amines that could be used for the one-pot mild synthesis of *N*-substituted 3,4-dihydroquinoxalin-2-one derivatives. Τhe discovery of post-modifications on the original MCR “core structure” is of great importance, as they can significantly enhance the diversity and complexity of the resulting compounds [35,36,37,38,39,40,41]. 

**Scheme 2:**
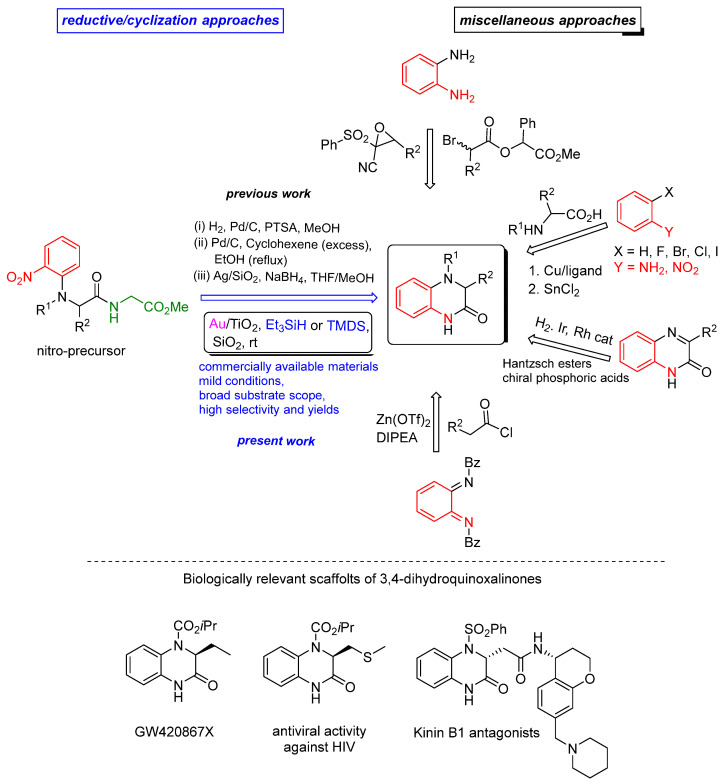
Synthetic approaches for the 3,4-dihydroquinoxalin-2-ones.

Dihydroquinoxalinones are bioactive compounds of high interest, due to their anticancer [42,43,44] and anti-inflammatory [45] properties, as well as their activity against neurological diseases [46] (Scheme 2). Dihydroquinaxolinone derivatives have been mainly synthesized via coupling of *o*-nitroaryl- or *o*-aminoaryl halides with amino acids [43,45,47,48,49,50,51,52,53,54], or transition metal-catalyzed asymmetric hydrogenation of the corresponding quinoxaline derivatives (Scheme 2) [55,56,57,58,59,60,61,62,63]. However, these methods required air-sensitive catalysts, toxic organic solvents, and several ligands. Few studies have also reported the nucleophilic substitution/cyclization of *o*-phenylendiamines with α-bromo-α-phenyl ester, α-sulfonyl epoxides or substituted trichloromethylcarbinols (Scheme 2) [42,64,65,66,67,68,69,70]. Recently, the hetero-Diels–Alder reaction of ketene enolates with ortho-benzoquinone diimides [68,69] and a photo-organocatalytic approach have been reported for the synthesis of substituted dihydroquinoxalinones [70] (Scheme 2). Nevertheless, several sequential steps involving protecting groups and specific pre-synthesized starting materials were required.

Herein, we report a two-step protocol for the mild synthesis of *N*-substituted 3,4-dihydroquinoxalin-2-ones. Initially, the Au-catalyzed heterogeneous selective reduction of the multifunctional nitro compounds into the corresponding amines was achieved. The presence of other easily reducible groups, such as carbonyl, amide, ester and halogen moieties, did not affect the chemoselectivity. Subsequent treatment with silica, as an acidic medium, led to the in situ intramolecular transesterification of the initially formed amine (Scheme 1). To the best of our knowledge, a limited number of studies have reported on the success of MCR products transformed into quinoxaline derivatives in the presence of Pd/C (10 mol%) as a catalyst (a) under hydrogenation and acidic conditions using *p*-toluenesulfonic acid (Scheme 2, (i)) [71], and (b) through transfer hydrogenation with an excess of cyclohexene under refluxed conditions in ethanol (catalyst/substrate ratio = 1/2) (Scheme 2, (ii)) [72]. However, prolonged reaction times and multistep workup protocols were proposed for the final product isolation. Recently, our research group reported the selective reduction of aromatic nitro compounds into amines and, subsequently, into substituted dihydroquinoxalinones using AgNPs supported on mesoporous silica and NaBH_4_ as the reducing agent (Scheme 2, (iii)) [34]. The present approach is an ideal venue for the use of the commercially available Au/TiO_2_-Et_3_SiH and Au/TiO_2_-TMDS catalytic systems towards the mild and efficient selective transfer hydrogenation of a series of multifunctional, aromatic nitro precursors into amines and the synthesis of *N*-substituted 3,4-dihydroquinoxalin-2-ones (Scheme 2, (iii)) [34].

## 2. Results and Discussion

### 2.1. Catalytic Conditions Evaluation

**Scheme 3:**
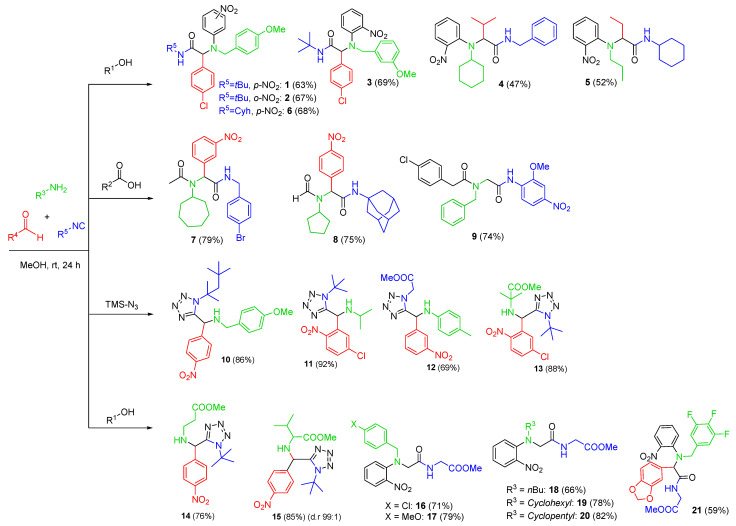
Synthetic procedures for multifunctional, aromatic nitro precursors **1**–**21**.

The synthesis of the multifunctional, aromatic nitro compounds **1**–**21** (Scheme 2) was performed based on the reported isocyanide-based MCRs [35,36,40,41,73,74]. Briefly, an equimolar mixture of the corresponding isocyanide and phenols (**1**–**6**, **16**–**21**), isocyanide and carboxylic acids (**7**–**9**), or isocyanide and TMSN_3_ (**10**–**15**) were added into the solution of an amine (5 mmol) and a carbonyl compound in methanol at ambient conditions (for details, see Materials and Methods) [75,76,77,78,79,80,81,82,83,84]. After stirring for the appropriate time, yellow or light-yellow products were isolated by silica gel column chromatography in good to high yields (Scheme 3). All nitro compounds were characterized by NMR and HRMS spectroscopy (see SI). 

To identify the optimum conditions of the present catalytic reaction, we first investigated the selective reduction of nitro derivative **1** into the corresponding amine **1a** (Table 1). The supported AuNP catalysts, i.e., Au/TiO_2_, Au/Al_2_O_3_ and Au/ZnO, as well as the complex Ph_3_PAuNTf_2_ and the salts HAuCl_4_ and AuCl, were used without further purification. All commercial heterogeneous catalysts feature a ca. 1 wt% Au loading and exhibit an average AuNP size of about 2–3 nm. In all cases, MeOH or an equimolar mixture of THF/MeOH were used as the reaction medium. In blank experiments, common reducing agents, including NaBH_4_, NH_3_BH_3_, LiBH_4_, LiAlH_4_, NH_3_NH_3_^.^H_2_O and NaH were found to be inactive in the absence of a catalyst (Table 1, entries 1–4). However, several degradation products, such as the cleavage of the *tert*-amide of the anisole group, were observed with ^1^H NMR of the crude mixtures in the case of LiAlH_4_ and NaH. In contrast, an efficient reduction was observed, based on TLC analysis, in the presence of Au/TiO_2_, when NaBH_4_, NH_3_BH_3_ and LiBH_4_ were used as reducing agents within THF/MeOH (1/1) (Table 1, entries 5–7). In addition, NaBH_4_ was found to be active within THF or MeOH (Table 1, entries 8 and 9), as well as in their mixtures of different ratios (results not shown); however, in the lower amount of 1 equiv. (based on the amount of **1**), a lower conversion was observed even at a prolonged reaction time of 18 h (Table 1, entry 10). Although the reactions showed high selectivity (>99%) based on the ^1^H NMR spectra of the crude mixtures, amine **1a** was isolated in low yields ranging between 48 and 84% (Table 1, entries 5–10), probably due to the difficult separation of **1a** by column chromatography. 

Based on these results and our previous mechanistic studies on AuNP-catalyzed selective reduction of nitroarenes into anilines in the presence of 1,1,3,3-tetramethyldisiloxane (TMDS) [29], we subsequently used a series of different hydrosilanes as hydrogen donor molecules. To improve the solubility of the starting material, the following screening tests were conducted in an equimolar mixture of THF/MeOH. Among the examined hydrosilanes (Table 1, entries 11–19), TMDS, dimethylphenylsilane (DMPS), diphenylsilane (DPS) and triethylsilane (Et_3_SiH) favored the completion of the transfer hydrogenation process in a fast and clean manner (Table 1, entries 12–18). In all cases, the desired amine (**1a**) was obtained, along with the corresponding silanols and/or silylethers as by-products as determined by TLC and ^1^H NMR analysis of the crude mixture [85], and was isolated in high yield after chromatographic purification. Especially when TMDS and Et_3_SiH were used as reducing agents, **1a** was observed in high yields within a short reaction time (Table 1, entries 12 and 18).

Hence, TMDS and Et_3_SiH were further used to evaluate the catalytic activity of Au catalysts. No reaction occurred in the presence of the gold salts AuCl_3_, AuCl and Ph_3_PAuNTf_2_, even after a prolonged reaction time (Table 1, entries 20–25). In contrast, AuNPs supported on alumina, Au/Al_2_O_3_, showed good activity towards the reduction of **1**, leading to 75% and 77% isolated yields of **1a** using Et_3_SiH and TMDS, respectively (Table 1, entries 26 and 27), whereas Au/ZnO was found to be inactive as a catalyst (Table 1, entries 28 and 29) under the present conditions. 

In addition, the homogeneous (AuCl_3_, AuCl) and heterogeneous (Au/TiO_2_) reactions in the presence of Et_3_SiH were performed upon irradiation for 3 h with a 300 W Xenon Arc Lamp (λ > 320 nm) as the light source. During irradiation, the reaction mixtures were thermostated with a water bath (ca. 28 °C). In all cases, no significant changes were observed in the reaction conversion and selectivity. However, in the latter case (Au/TiO_2_/Et_3_SiH/hv), a mixture of the desired amine with unidentified degradation products was observed with the ^1^H NMR spectrum of the crude mixtures (results not shown). 

### 2.2. Catalytic Application for the Sythesis of Several Multifunctional Amines

**Scheme 4:**
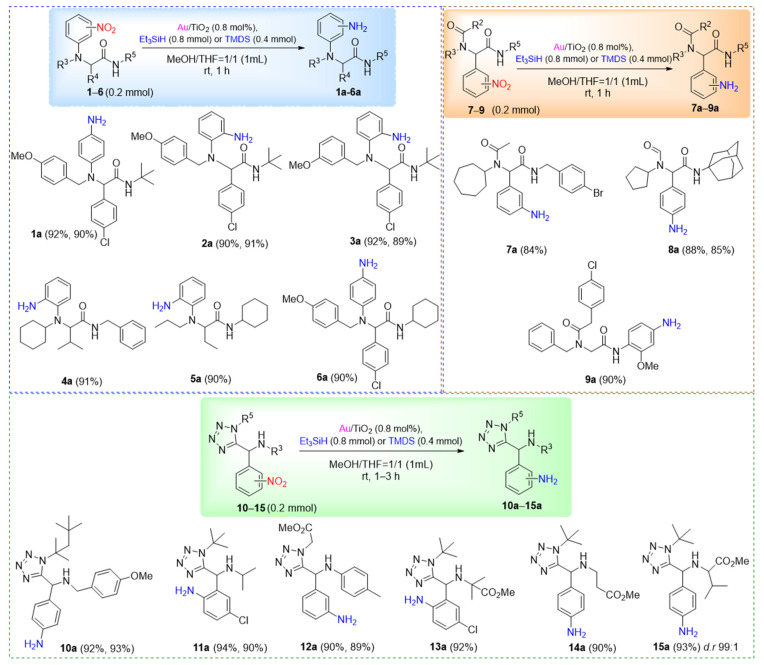
Au/TiO_2_-catalyzed chemoselective reduction of the multifunctional, aromatic nitro compounds **1**–**15** in the presence of Et_3_SiH or TMDS.

To explore the substrate broadness of the described catalytic reduction process, the synthesized multifunctional nitro compounds **1**–**15** were screened under the optimal catalytic conditions, using Au/TiO_2_-Et_3_SiH (4 eq.) (Scheme 4). To our delight, the corresponding amine derivatives **1a**–**15a** were selectively formed in high conversions and isolated yields (84–94%) in a relatively short reaction time (1–3 h), as shown in Scheme 4. Interestingly, easily reducible moieties, such as chloro, carbonyl and carboxyl groups, were well tolerated under the present conditions. Under the present mild conditions, the measured diastereoselectivity (*d.r. = 99/1*) of the amine **15a** corresponds to the initial ratio of the corresponding starting nitro compound **15**, as concluded by ^1^H NMR (SI). For comparison, TMDS (2 eq.) was also used in the reduction of **1**–**3**, **8** and **10**–**12** in the presence of Au/TiO_2_ and at ambient conditions. In all cases, high isolated yields (85–93%) for the amines **1a**–**3a**, **8a** and **10a**–**12a** were measured (second value in parentheses, Scheme 4) within a short reaction time of 1 h. These results confirm the high catalytic activity of Au/TiO_2_-Et_3_SiH or Au/TiO_2_-TMDS for the chemoselective reduction of such multifunctional, aromatic nitro compounds into the corresponding amines, and suggest that this approach can be used as a facile and useful strategy for multigram scale synthesis. To that end, 1 mmol of **1** and **14** were reduced by 5 mmol of Et_3_SiH (or 2.5 mmol of TMDS) in 8 mL of MeOH/THF (1/1), in the presence of 0.6 mol% of Au/TiO_2_ (1% wt) at rt. After reaction completion (3 h, according to the TLC), the reaction mixture was centrifuged to separate the solid catalyst, and the residue was purified by silica gel column chromatography, affording the desired amines **1a** (81% using Et_3_SiH and 83% using TMDS) and **14a** (82% using Et_3_SiH and 80% using TMDS). 

### 2.3. Synthetic Application towards the One-Pot Synthesis of 3,4-Dihydroquinoxalin-2-Ones

**Scheme 5:**
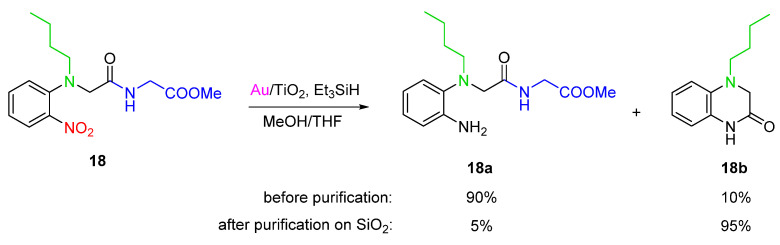
Reaction profile of the Au/TiO_2_-Et_3_SiH-catalyzed reduction of **18**.

The reduction of *ortho*-phenyl-substituted nitro compound **18** formed the corresponding amine **18a** in a 90% relative yield along with a small amount of the 3,4-dihydroquinoxalin-2-one **18b** (~10%), according to ^1^H NMR analysis of the crude reaction mixture. After chromatographic purification (with silica gel), the initial ratio of **18a**/**18b** changed from 90/10 to 5/95 (Figure S1), indicating that the heterocycle **18b** was undoubtedly formed from **18a** via a silica-acid-catalyzed one-pot ring-closing pathway involving an intramolecular C-N cyclization process. This pathway was observed when the starting material contains a methyl carbamate group (such as in substrate **18**); however, no cyclization products were formed when simple alkyl, cycloalkyl or benzyl moieties were present (i.e., substrates **3a**–**5a**) (Scheme 5 and Figure S1). This intramolecular transamidation probably occurred due to the rapid elimination of the glycine methyl ester as a good living group under the intramolecular nucleophilic pathway. 

**Scheme 6:**
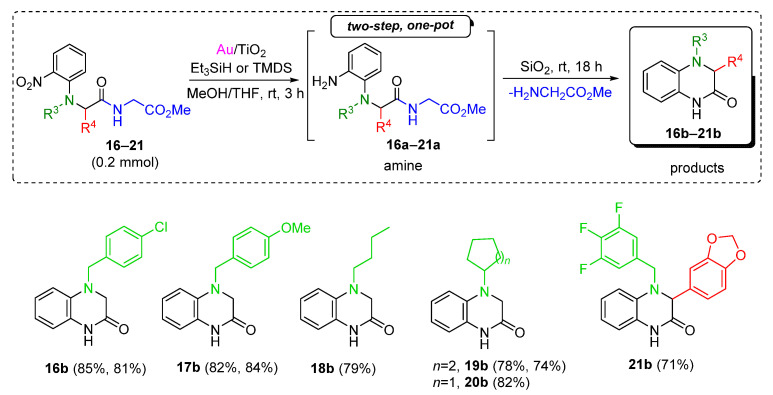
Synthesis of 3,4-dihydroquinoxalin-2-ones (**16b**–**21b**) in the presence of Au/TiO_2_-Et_3_SiH or Au/TiO_2_-TMDS.

The reaction profile of amine **18a** was studied in deuterated solvent (CDCl_3_) and monitored directly by ^1^H NMR after the addition of a small amount of silica gel. After 8 h, a mixture of the amine **18a**, the 3,4-dihydroquinoxalin-2-one **18b** and the glycine methyl ester **C** was observed (Figure S2). This observation prompted us to further investigate the plausible one-pot, two-step facile synthesis of a series of 3,4-dihydroquinoxalin-2-ones **16b**–**21b**, as shown in Scheme 6. Specifically, the corresponding nitro compounds **16**–**21** were selectively reduced under the present heterogeneous transfer hydrogenation process (Au/TiO_2_-Et_3_SiH), on a 0.2 mmol reaction scale. After the reduction completion (3 h, based on TLC analysis), a small amount of SiO_2_ (ca. 20 mg) was added and the mixture stirred for an additional 18 h (TLC monitoring). In all cases, good to high isolated yields (71–85%, first value in parentheses) were measured for the final desired cyclic products **21b**–**27b**, based on the amount of the initial nitro compound (Scheme 6). The corresponding amines **16a**–**21a** could also be isolated in high yields (89–96%) immediately after reduction completion (ca 3 h, results not shown), using neutralized silica for the purification procedure (see Materials and Methods). Furthermore, the Au/TiO_2_-TMDS was also used for the reduction of **16**, **17** and **19**. In these reductive/cyclization processes, good to high isolated yields were measured for the corresponding 3,4-dihydriquinoxalin-2-ones **16b**, **17b** and **19b** with the values of 81%, 84% and 74%, respectively. These results support the clean and mild efficacy of the present heterogeneous AuNP catalytic process. To the best of our knowledge, this is the first study based on the gold nanoparticle heterogeneous catalysis of the reductive/cyclization transformation of several MCR, multifunctional nitro compounds into 3,4-dihydroquinoxalin-2-ones. 

**Scheme 7:**
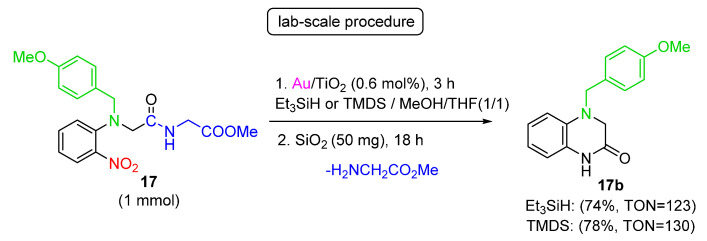
Au/TiO_2_-Et_3_SiH or Au/TiO_2_-TMDS catalytic system application to the lab-scale synthesis of **17b** from **17**.

Based on these encouraging results, the Au/TiO_2_-Et_3_SiH and Au/TiO_2_-TMDS catalytic systems towards dihydroquinoxalino-2-ones were further tested at a lab-scale. Thus, 1 mmol of the nitro derivative **17** was reduced in the presence of Au/TiO_2_ 1% wt (120 mg, 0.006 mmol) with 5 mmol of Et_3_SiH or 2.5 mmol of TMDS in 10 mL THF/MeOH (1/1). After the reduction was completed (3 h, based on TLC), 50 mg of silica gel was added in each reaction mixture and stirred for 18 h (monitored by TLC). The corresponding 3,4-dihydroquinoxalin-2-one **17b** was isolated after chromatographic purification (see Materials and Methods), in 74% and 78% yields (TON = 123 and 130, Scheme 7). When a lower catalyst amount (0.4 mmol) was used, no reaction completion was observed by TLC analysis. 

### 2.4. Library Analysis of the Dihydroquinoxalin-2-Ones

**Figure 1:**
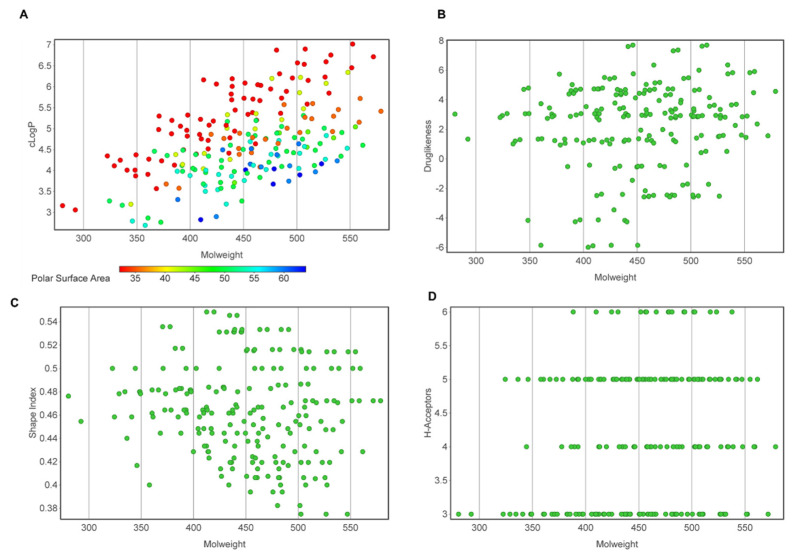
(**A**) Scatterplot of clogP vs. MW. The marker color is defined by the polar surface area values; (**B**) scatterplot of drug-likeness vs. MW; (**C**) scatterplot of shape index vs. MW; (**D**) scatterplot of HBA vs. MW.

Dihydroquinoxaline belongs to the so-called privileged structures. We created a random >200-member virtual library based on this scaffold by using the DataWarrior software (see SI) [86]. Next, we calculated certain physicochemical parameters of this library such as molecular weight (MW), lipophilicity (clogP), polar surface area (PSA), number of hydrogen bonds acceptors and donors (HBA, HBD), drug-likeness and shape index (Figure 1). clogP, MW and PSA are key parameters determining important drug properties such as oral bioavailability and metabolism. Our analysis showed that 60% of our virtual library obeyed the rule of five (Ro5). Moreover, there is a strong drug-like profile (positive values, Figure 1B) as 80% of our library fell into these constraints. Regarding the shape index, using the parameters of spherical <0.5 and linear >0.5, a more spherical type of shape was observed overall.

## 3. Conclusions

In conclusion, we showed that titania-supported gold nanoparticles can be applied to more complicated organic compounds and catalyze the reduction of a series of multifunctional, aromatic nitro compounds into the corresponding amine derivatives using Et_3_SiH or TMDS as reducing agents. The Au/TiO_2_-Et_3_SiH and Au/TiO_2_-TMDS catalytic systems were also found to be highly chemoselective, even for more complex molecules such as tetrazole and amino acid-substituted derivatives, serving as a rapid approach towards *N*-substituted 3,4-dihydroquinoxalin-2-ones. The proposed one-pot, two-step, acid-promoted ring-closing reaction pathway afforded the desired products in good to high isolated yields, even at the lab-scale. The key parameters clogP, MW and PSA were also calculated; thus, important related drug properties, such as oral bioavailability and metabolism, were also reported.

## 4. Materials and Methods

### 4.1. General and Aparatus 

All the reagents and solvents were purchased from Sigma-Aldrich, AK Scientific, Fluorochem, Abcr GmbH, Acros, and were used without further purification. Thin-layer chromatography was performed on Millipore precoated silica gel plates (0.20 mm thick, particle size 25 μm). Nuclear magnetic resonance spectra were recorded on a Bruker Avance 500 or 600 spectrometer and on Agilent 500 {^1^H NMR (500 MHz; 600 MHz), ^13^C{H} NMR (125 MHz; 150 MHz)}. Chemical shifts for ^1^H NMR were reported as *δ* values and coupling constants were in hertz (Hz). The following abbreviations were used for spin multiplicity: s = singlet, br s = broad singlet, d = doublet, t = triplet, q = quartet, quin = quintet, dd = double of doublets, ddd = double doublet of doublets, m = multiplet. Chemical shifts for ^13^C{H} NMR were reported in ppm relative to the solvent peak. Light Source: The reaction mixtures were irradiated for an appropriate time with a variac Cermax**^®^** 300W Xenon Arc Lamp (λ > 320 nm) as the light source (PE300BF CERMAX**^®^** XENON ARC LAMPS, 300W, Cat No: CE-303/540). Please refer to the homepage of PerkinElmer Optoelectronics (https://www.perkinelmer.com/, accessed on 8 June 2022) for further details. Flash chromatography was performed on a Reveleris**^®^** X2 Flash Chromatography System, using Grace**^®^** Reveleris Silica Flash Cartridges (12 g). Mass spectra were measured on a Waters Investigator Supercritical Fluid Chromatography System with a 3100 MS Detector (ESI) using a solvent system of methanol and CO_2_ on a Viridis silica gel column (4.6 × 250 mm, 5 µm particle size) or Viridis 2-ethylpyridine column (4.6 × 250 mm, 5 µm particle size). Mass spectra (HRMS) were determined through electrospray ionization mass spectrometry (ESI-MS) by using a Thermo Fisher Scientific (Bremen, Germany) model, the LTQ Orbitrap Discovery MS, at a flow rate of 10 μL/min using a syringe pump. The infusion experiments were run using a standard ESI source operating in a positive ionization mode. Source operating conditions were a 3.7 kV spray voltage and a 300 °C heated capillary temperature. 

### 4.2. Synthesis of Nitro Compounds via the Ugi-Smiles MCR 

A solution of the corresponding aldehyde (5.0 mmol), nitro-substituted phenol (5.0 mmol) and amine (5.0 mmol) in methanol (5 mL) was stirred at room temperature for 15 min. Subsequently, isocyanide (5.0 mmol) was added and the reaction was stirred at room temperature for approximately 20 h. After reaction completion, monitored by TLC, the reaction mixture was concentrated *in vacuo* and the residue was purified by column chromatography using silica and an eluent solvent mixture of hexane/ethyl acetate in a ratio from 5:1 to 1:3, affording the desired nitro compounds **1**–**6** and **16**–**21** in good to high yields [73,87].

### 4.3. Synthesis of Nitro Compounds via the Ugi-Tetrazole MCR 

A solution of the corresponding aldehyde or ketone (5.0 mmol) and amine or amino acid methylester (5.0 mmol) in methanol (5 mL) was stirred at room temperature for 15 min. Subsequently, isocyanide (5.0 mmol) and TMSN_3_ (5.0 mmol, 665 μL) were added and the reaction mixture was stirred at room temperature for approximately 20 h. After reaction completion, monitored by TLC, the reaction mixture was concentrated *in vacuo* and the residue was purified by column chromatography using silica and an eluent solvent mixture of hexane/ethyl acetate in a ratio from 5:1 to 1:5, affording the desired compounds **10**–**15** in high yields [87].

### 4.4. Synthesis of Nitro Compounds via the Ugi-Four MCR

A solution of the corresponding aldehyde (5.0 mmol), carboxylic acid (5.0 mmol) and amine (5.0 mmol) in methanol (5 mL) was stirred at room temperature for 15 min. Subsequently, isocyanide (5.0 mmol) was added and the reaction was stirred at room temperature for approximately 20 h. After reaction completion, monitored by TLC, the reaction mixture was concentrated *in vacuo* and the residue was purified by column chromatography using silica and an eluent solvent mixture of hexane/ethyl acetate in a ratio from 5:1 to 1:2, affording the desired compounds **7**–**9** in good to high yields [87].

### 4.5. Catalytic Reduction for Synthesis of the Corresponding Amines ***1a**–**21a***

To a sealed tube containing the corresponding nitro-substituted compounds (0.2 mmol) in 1 mL THF/MeOH/ (1/1), Et_3_SiH (0.8 mmol) or TMDS (0.4 mmol) and Au/TiO_2_, 1 % wt (0.8 mol%, 30 mg) was added. The reaction mixture was stirred at room temperature for the appropriate time. After reaction completion (monitored by TLC), the reaction mixture was centrifuged to separate the solid catalyst, and the cake was spray-washed with MeOH (~10 mL). The solvents were removed under vacuum and the residue was chromatographed on neutralized silica gel by using hexane/EtOAc mixtures from 10:1 to 1:1 as eluent, to give the corresponding amines **1a**–**21a** in pure form. It is worth noting that the amines in most cases were unstable during the chromatographic purification procedure; for this reason, neutralized silica (with the addition of a few drops of triethylamine in the eluent solvent mixture) was used for the column chromatography, and pre-treated CDCl_3_ with anhydrous K_2_CO_3_ was used as the NMR solvent.

### 4.6. Lab-Scale Reduction for Synthesis of ***1a*** and ***14a***

To a sealed tube containing the corresponding nitro-substituted compounds **1** or **14** (1 mmol) in 8 mL THF/MeOH (1/1), Et_3_SiH (5 mmol) or TMDS (2.5 mmol) and Au/TiO_2_, 1 % wt (0.6 mol%, 120 mg) was added. The reaction mixture was stirred at room temperature for the appropriate time (3 h). After reaction completion (monitored by TLC), the slurry was centrifuged to separate the catalyst, and the solid residue was washed with MeOH (~10 mL). The solvent was then removed under vacuum and the residue was purified by column chromatography on neutralized silica (with the addition of a few drops of triethylamine in the eluent solvent mixture) by using hexane/EtOAc mixtures from 5:1 to 1:5 as eluent, affording the corresponding amines **1a** and **14a** in pure forms, at 365 mg (81% yield) and 272 mg (82% yield), respectively.

### 4.7. One-Pot Process for Synthesis of the 3,4-Dihydroquinoxalin-2-Ones ***16b**–**21b***

To a sealed tube containing the corresponding nitro compounds (0.2 mmol) in 1 mL THF/MeOH (1/1), Et_3_SiH (0.8 mmol) or TMDS (0.4 mmol) and Au/TiO_2_, 1 % wt (0.8 mol%, 30 mg) was added. The reaction mixture was stirred at room temperature for the appropriate time and was monitored by TLC. After completion of the reduction, silica gel (20 mg) was added in the mixture, forming the corresponding cyclized adducts (18 h). After that, the slurry was filtered to remove the catalyst and the filter cake was washed with MeOH (~5 mL). The solvents of the filtrate were evaporated under vacuum and the resulting residues lead to the corresponding 3,4-dihydroquinoxalin-2-one derivatives **16b**–**21b** in pure forms, after chromatographic purification with hexane/ethyl acetate from 30:1 to 2:1 as eluent.

### 4.8. Lab-Scale Synthesis of Dihydroquinoxalin-2-One ***17b***

To a sealed tube containing the corresponding nitro compound **17** (1 mmol) in 10 mL THF/MeOH (1/1), Et_3_SiH (5 mmol) or TMDS (2.5 mmol) and Au/TiO_2_, 1 % wt (0.6 mol%, or 0.006 mmol, 120 mg) was added. The reaction mixture was stirred at room temperature for the appropriate time and was monitored by TLC (ca. 3 h). After completion of the reduction, silica gel (50 mg) was added in the mixture, forming the corresponding cyclized adduct (18 h). After that, the slurry was filtered to remove the catalyst and the silica, and the filter cake was washed with MeOH (~10 mL). The solvents of the filtrate were evaporated under vacuum and the resulting residue lead to the corresponding 3,4-dihydroquinoxalin-2-one **17b** in pure forms (Et_3_SiH: 74% yield, 198 mg and TMDS: 78% yield, 205 mg), after chromatographic purification as described above with hexane/ethyl acetate from 20:1 to 2:1 as eluent.

## Figures and Tables

**Table 1:** Evaluation of Au catalysts, reducing agents and solvents in the catalytic reduction of **1** into **1a**.

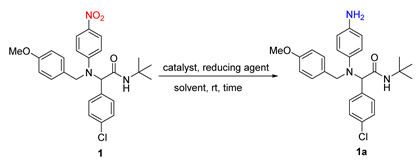					
Entry	Catalyst ^[a]^	Solvent ^[a]^	Reducing Agent (eq.) ^[a]^	Time (h)	1%/1a% ^[b]^
1	-	THF/MeOH	NaBH_4_ or LiBH_4_ (2)	18	100/0
2	-	THF/MeOH	NH_3_BH_3_ (2)	18	100/0
3 ^[c]^	-	THF	LiAlH_4_ (2)	18	43/0
4 ^[c]^	-	THF	NaH (3)	18	47/0
5	Au/TiO_2_	THF/MeOH	NH_3_BH_3_ (2)	18	0/>99 (78)
6	Au/TiO_2_	THF/MeOH	LiBH_4_ (2)	1	0/>99 (62)
7	Au/TiO_2_	THF/MeOH	NaBH_4_ (2)	1	0/>99 (81)
8	Au/TiO_2_	THF	NaBH_4_ (2)	24	0/>99 (79)
9	Au/TiO_2_	MeOH	NaBH_4_ (2)	18	0/>99 (84)
10	Au/TiO_2_	THF/MeOH	NaBH_4_ (1)	18	40/60 (48)
11	-	THF/MeOH	TMDS (2)	24	100/0
12	Au/TiO_2_	THF/MeOH	TMDS (2)	1	0/>99 (89)
13	Au/TiO_2_	THF/MeOH	TMDS (1)	1	37/63 (55)
14	Au/TiO_2_	MeOH	TMDS (2)	24	0/>99 (85)
15	Au/TiO_2_	THF/MeOH	DMPS (4)	1	0/>99 (81)
16	Au/TiO_2_	THF/MeOH	DPS (4)	1.5	0/>99 (85)
17	Au/TiO_2_	THF/MeOH	(EtO)_3_SiH (4)	24	100/0
18	Au/TiO_2_	THF/MeOH	Et_3_SiH (4)	1	0/>99 (92)
19	Au/TiO_2_	THF/MeOH	(TMS)_3_SiH (4)	2	27/73 (61)
20 ^[c,d]^	AuCl_3_	THF/MeOH	Et_3_SiH (4)	3	74/0
21 ^[c,d]^	AuCl_3_	THF/MeOH	TMDS (2)	1	71/0
22 ^[c,d]^	AuCl	THF/MeOH	Et_3_SiH (4)	3	74/0
23 ^[c,d]^	AuCl	THF/MeOH	TMDS (2)	1	79/0
24 ^[c,d]^	Ph_3_PAuNTf_2_	THF/MeOH	Et_3_SiH (4)	3	78/0
25 ^[c,d]^	Ph_3_PAuNTf_2_	THF/MeOH	TMDS (2)	1	82/0
26 ^[c]^	Au/Al_2_O_3_	THF/MeOH	Et_3_SiH (4)	2	0/80 (75)
27 ^[c]^	Au/Al_2_O_3_	THF/MeOH	TMDS (2)	2	0/83 (77)
28 ^[c]^	Au/ZnO	THF/MeOH	Et_3_SiH (4)	3	81/0
29	Au/ZnO	THF/MeOH	TMDS (2)	3	97/3

^[a]^ Conditions: 20 mg of Au/TiO_2_ (or Au/Al_2_O_3_ or Au/ZnO) 1 wt% (ca. 0.8 mol% based on **1**), 0.1 mmol of **1**, 1 mL of solvent (THF/MeOH = 1/1) and at rt. AuCl_3_, AuCl and Ph_3_PAuNTf_2_ were used in 20 mol% amount. ^[b]^ Relative yields of **1** and **1a** at appropriate time measured by ^1^H NMR of the crude reduction mixture. Values in parentheses correspond to the isolated yields. ^[c]^ Unidentified products missing either the tert-amide or the anisole group from its structure as determined by ^1^H NMR in some cases. ^[d]^ All the salts were used in 20 mmol%.

## Data Availability

Not applicable.

## Supplementary Materials

The following supporting information can be downloaded at: https://www.mdpi.com/article/10.3390/molecules27144395/s1, Figures S1–S3: NMR data and spectra of nitro compounds, amines and 3,4-dihydroquinoxalin-2-ones.

## Sample Availability

Not available.
