# Supplementary material for: Supported Gold Nanoparticle-Catalyzed Selective Reduction of Multifunctional, Aromatic Nitro Precursors into Amines and Synthesis of 3,4-Dihydroquinoxalin-2-Ones
Source: Molecules. 2022 Jul 8;27(14):4395. doi: 10.3390/molecules27144395 (PMC9323044; doi:10.3390/molecules27144395)

Supplementary Materials

Supported Gold Nanoparticle Catalyzed Selective Reduction of Multi-functional Aromatic Nitro Precursors into Amines and Synthesis of 3,4-Dihydroquinoxalin-2-ones

Domna Iordanidou[1,5], Michael G. Kallitsakis[1], Marina A. Tzani[1], Dimitris I. Ioannou[1], Tryfon Zarganes-Tzitzikas[2], Constantinos G. Neochoritis[3], Alexander Dömling[4], Michael A. Terzidis[5,*] and Ioannis N. Lykakis[1,*]

[1] Department of Chemistry, Aristotle University of Thessaloniki, University Campus 54124, Thessaloniki, Greece; E-mail: D.I.: domy-jo@hotmail.com; M.G.K.: kallitsos29@gmail.com; M.A.Tz.: marina_tzani@hotmail.com; D.I.I.: ioannou.dim1996@gmail.com; I.N.L.: lykakis@chem.auth.gr

[2] Alzheimer's Research UK Oxford Drug Discovery Institute, University of Oxford, Oxford, UK; Target Discovery Institute, NDM Research Building, University of Oxford, Old Road Campus, Roosevelt Drive, Oxford;.E-mail: tryfon.zarganis-tzitzikas@ndm.ox.ac.uk

[3] Department of Chemistry, University of Crete, Voutes Heraklion, Crete, Greece; E-mail: C.G.N.: kneochor@uoc.gr

[4] Department of Pharmacy, Drug Design group, University of Groningen, A. Deusinglaan 1, Groningen 9700 AV, The Netherlands; E-mail: a.s.s.domling@rug.nl

[5] Department of Nutritional Sciences and Dietetics, International Hellenic University, Sindos Campus 57400, Thessaloniki, Greece; E-mail: mterzidis@ihu.gr (M.A.T.: mterzidis@ihu.gr)

* Correspondence: Ioannis N. Lykakis – Department of Chemistry, Aristotle University of Thessaloniki, 54124 Thessaloniki, Greece; https://orcid.org/0000-0001-8165-5604; Email: lykakis@chem.auth.gr

**Table of contents**	Page
**Figure S1.** Study on the amines transformation into the corresponding 3,4-dehydroquinoxalinones on SiO_2	2
**Figure S2.** ^1H NMR spectra of the crude mixture of the *in situ* C-N cyclization of amine **18a** into the corresponding 3,4-dihydroquinoxalin-2-one **18b**, in $CDCl_3$ (t=1 and 8h)	3
**Figure S3.** Library analysis	4
^1H, $^{13}C\{H\}$ and ^{19}F NMR data of nitro compounds **1-21**	5-9
^1H and $^{13}C\{H\}$ NMR data of amines **1a-21a**	10-14
^1H and $^{13}C\{H\}$ NMR data of 3,4-dihydroquinoxalin-2-ones **16b-21b**	15-16
Copies of ^1H, $^{13}C\{H\}$ and ^{19}F NMR spectra of nitro compounds **1-21**	17-38
Copies of ^1H and $^{13}C\{H\}$ NMR spectra of amines **1a-21a**	39-59
Copies of ^1H and $^{13}C\{H\}$ NMR spectra of 3,4-dihydroquinoxalin-2-ones **16b-21b**	60-65

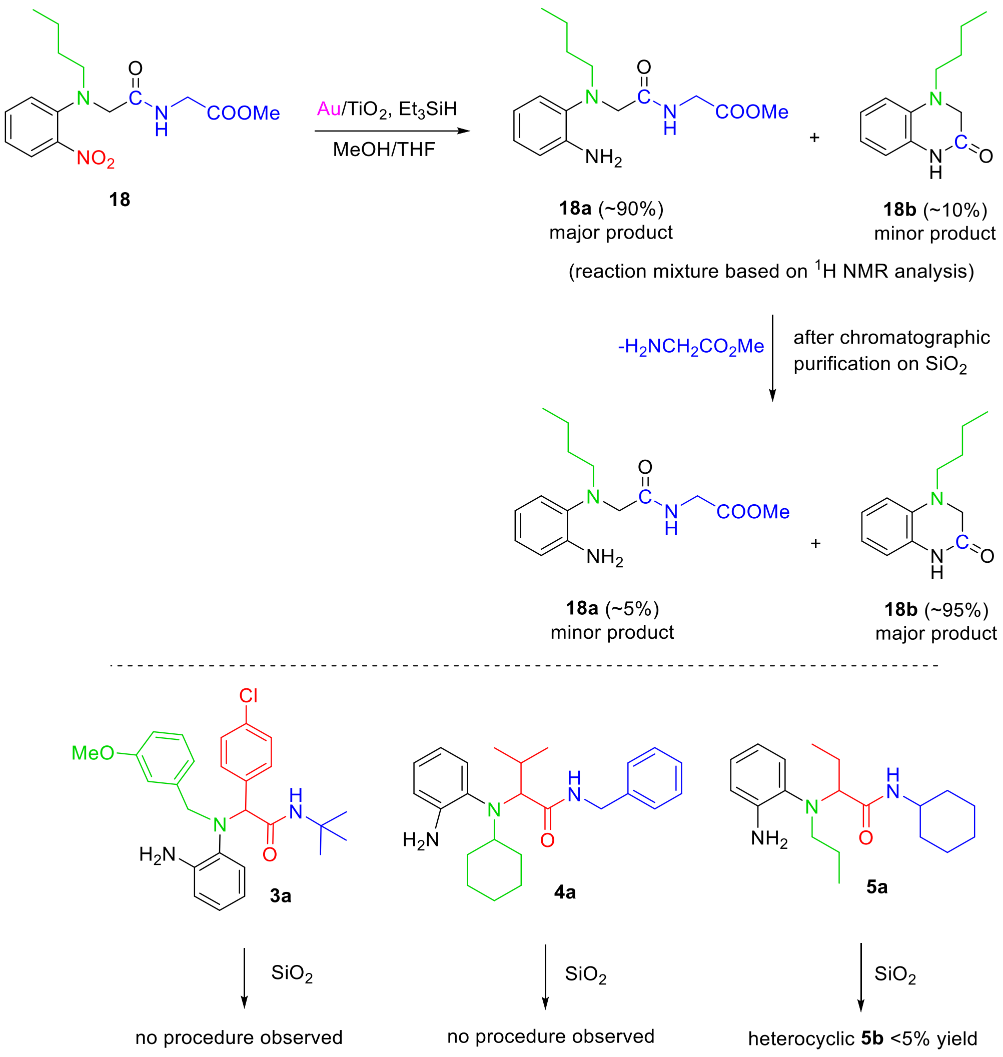

Figure S1. Study on the amines transformation into the corresponding 3,4-dehydroquinoxalinones on SiO_2

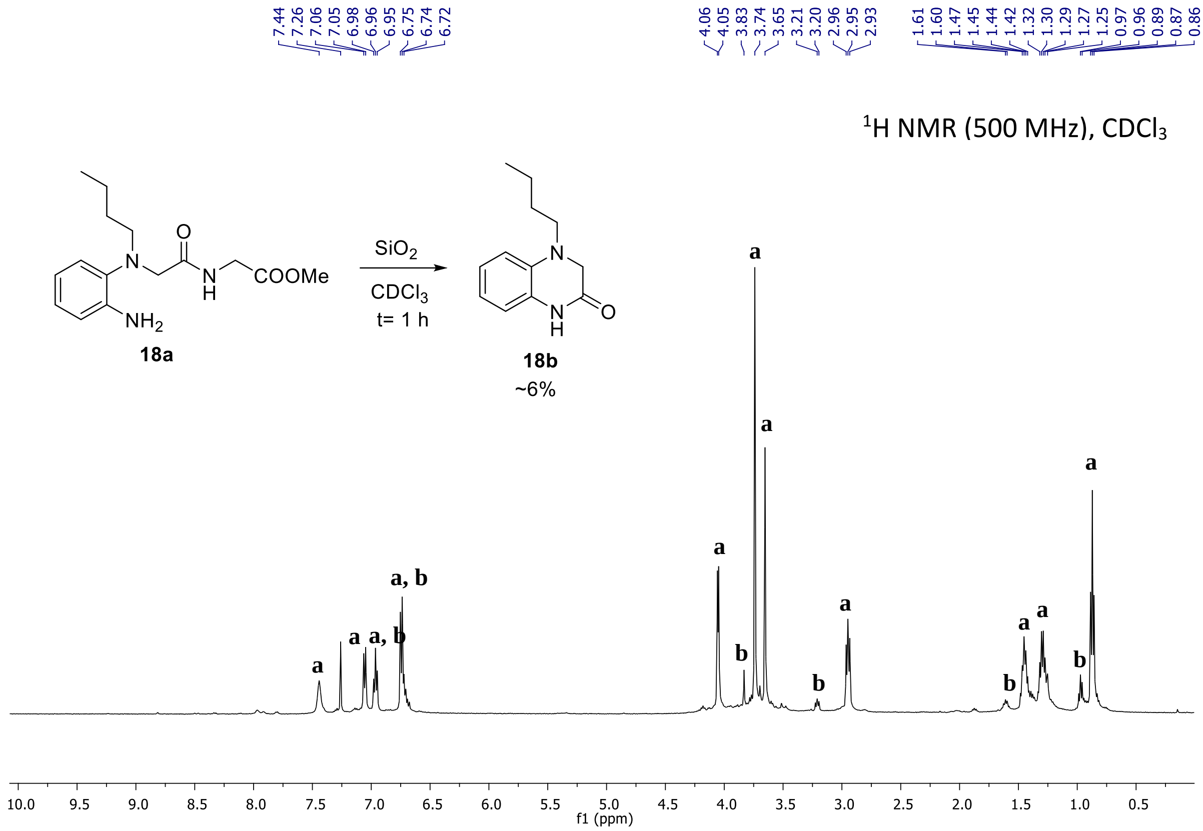

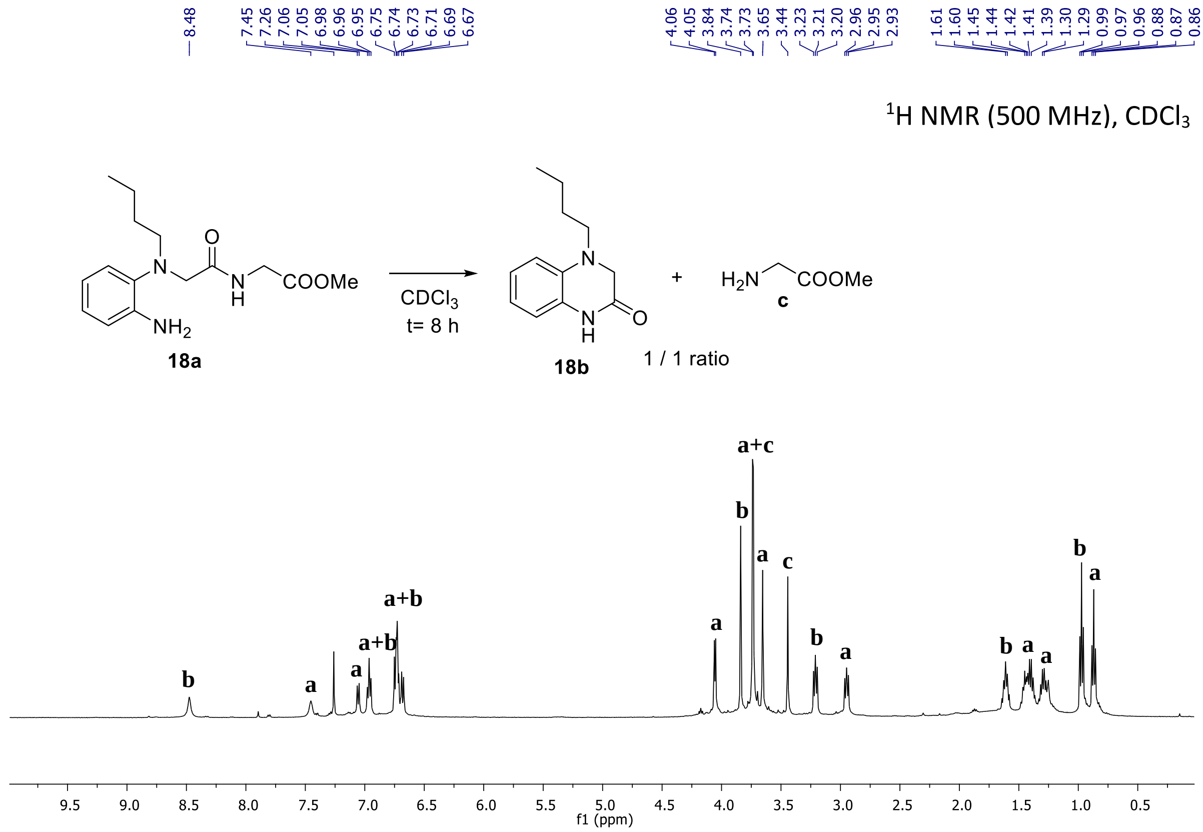

Figure S2. ^{1}H NMR spectra of the crude mixture of the *in situ* C-N cyclization process of amine **18a** into the corresponding dihydroquinoxalinone **18b**, in $CDCl_3$ (t=1 and 8h)

Via the software DataWarrior, we enumerate a combinatorial library of 210 dihydroquinoxalines. We employed 6, 6, 8 different, commercially available phenols, amines and aldehydes (Enamine, > 80 USD), respectively.

Phenols

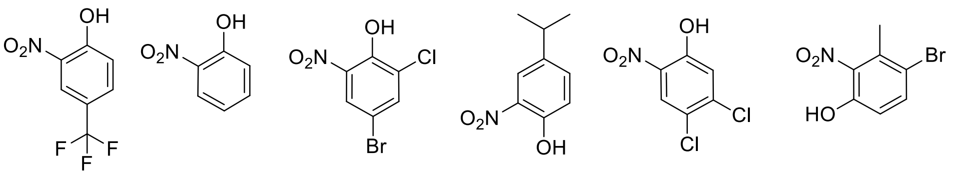

Amines

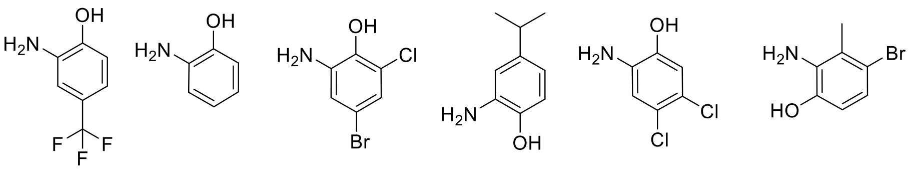

Aldehydes

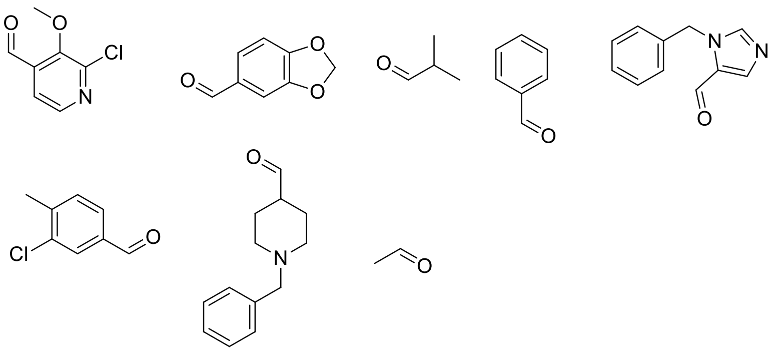

Figure S3. Library analysis

^{1}H, ^{13}C{H} and ^{19}F NMR data for nitro compounds 1-21

N-(tert-butyl)-2-(4-chlorophenyl)-2-((4-methoxybenzyl)(4-nitrophenyl)amino)acetamide (**1**): Yellow solid, 1515 mg, yield 63%; ^{1}H NMR (500 MHz, $CDCl_3$): 8.07 (d, 2H, J = 9.3 Hz), 7.28 (d, 2H, J = 8.5 Hz), 7.20 (d, 2H, J = 8.6 Hz), 7.00 (d, 2H, J = 8.6 Hz), 6.80 (dd, 4H, J_1 = 9.1 Hz, J_2 = 2.5 Hz), 5.80 (s, 1H), 5.38 (s, 1H), 4.69 (d, 1H, J = 17.2 Hz), 4.49 (d, 1H, J = 17.2 Hz), 3.77 (s, 3H), 1.25 (s, 9H); ^{13}C{H} NMR (125 MHz, $CDCl_3$): 168.1, 159.1, 153.7, 139.2, 134.8, 133.1, 130.7 (2C), 129.2, 129.1 (2C), 127.8 (2C), 125.9 (2C), 114.4 (2C), 113.2 (2C), 67.8, 55.5, 52.5, 52.2, 28.6 (3C); HRMS (ESI) *m/z*: $[M+H]^+$ calcd for $C_{26}H_{29}ClN_3O_4$ 482.1847, found 482.1838.	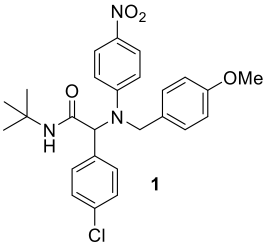
N-(tert-butyl)-2-(4-chlorophenyl)-2-((4-methoxybenzyl)(2-nitrophenyl)amino)acetamide (**2**): Orange oil, 1611 mg, yield 67%; ^{1}H NMR (500 MHz, $CDCl_3$): 7.64 (d, 1H, J = 8.0 Hz), 7.41 (d, 2H, J = 8.3 Hz), 7.31 (d, 3H, J = 8.3 Hz), 7.19 (t, 1H, J = 7.7 Hz), 7.14 (br s, 1H), 6.91 (d, 1H, J = 8.0 Hz), 6.68 (d, 2H, J = 8.4 Hz), 6.62 (d, 2H, J = 8.4 Hz), 4.68 (s, 1H), 3.93 (s, 2H), 3.74 (s, 3H), 1.12 (s, 9H); ^{13}C{H} NMR (125 MHz, $CDCl_3$): 169.2, 159.4, 147.6, 142.0, 135.2, 134.2, 132.5, 130.9 (2C), 130.0 (2C), 129.0 (2C), 127.6, 126.2, 125.6, 124.5, 113.7 (2C), 71.7, 57.1, 55.3, 51.1, 28.4 (3C); HRMS (ESI) *m/z*: $[M+H]^+$ calcd for $C_{26}H_{29}ClN_3O_4$ 482.1847, found 482.1838.	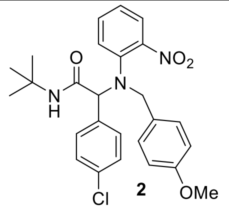
*N-(tert-butyl)-2-(4-chlorophenyl)-2-((3-methoxybenzyl)(2-nitrophenyl)amino)acetamide (**3**):* Yellow solid, 1660 mg, yield 69%; ^{1}H NMR (500 MHz, $CDCl_3$): 7.66 (dd, 1H, J_1 = 8.0 Hz, J_2 = 1.3 Hz), 7.40 (d, 2H, J = 8.4 Hz), 7.32-7.35 (m, 3H), 7.19 (t, 1H, J = 7.7 Hz), 7.12 (br s, 1H), 7.07 (t, 1H, J = 7.9 Hz), 6.95 (d, 1H, J = 8.1 Hz), 6.75 (dd, 1H, J_1 = 8.3 Hz, J_2 = 2.3 Hz), 6.34 (d, 1H, J = 7.5 Hz), 6.28 (br t, 1H), 4.73 (s, 1H), 3.98 (q, 2H, J = 13.5 Hz), 3.66 (s, 3H), 1.14 (s, 9H); ^{13}C{H} NMR (125 MHz, $CDCl_3$): 169.0, 159.6, 147.4, 142.0, 135.9, 134.8, 134.3, 132.6, 130.2 (2C), 129.4, 129.1 (2C), 127.3, 125.6, 124.7, 121.8, 114.7, 113.9, 71.9, 57.2, 55.2, 51.2, 28.4 (3C); HRMS (ESI) *m/z*: $[M+H]^+$ calcd for $C_{26}H_{29}ClN_3O_4$ 482.1847, found 482.1840.	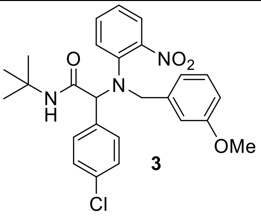
*N-benzyl-2-(cyclohexyl(2-nitrophenyl)amino)-3-methylbutanamide (**4**):* Orange oil, 961 mg, yield 47%; ^{1}H NMR (500 MHz, $CDCl_3$): 7.74-7.77 (m, 1H), 7.48 (d, 1H, J = 8.0 Hz), 7.44 (t, 1H, J = 7.7 Hz), 7.31 (d, 1H, J = 8.0 Hz), 7.19 (t, 1H, J = 7.7 Hz), 7.14-7.16 (m, 3H), 7.01-7.03 (m, 1H), 4.47-4.51 (m, 1H), 4.10 (dd, 1H, J_1 = 14.4 Hz, J_2 = 4.8 Hz), 3.97 (d, 1H, J = 4.3 Hz), 2.78-2.84 (m, 1H), 2.16-2.22 (m, 1H), 1.75 (dt, 1H, J_1 = 11.9 Hz, J_2 = 3.2 Hz), 1.57-1.66 (m, 3H), 1.43 (dt, 1H, J_1 = 13.1 Hz, J_2 = 3.1 Hz), 1.08-1.15 (m, 1H), 1.05 (d, 3H, J = 6.9 Hz), 0.96-1.02 (m, 1H), 0.89 (d, 3H, J = 6.7 Hz), 0.65-0.78 (m, 3H); ^{13}C{H} NMR (125 MHz, $CDCl_3$): 170.6, 149.8, 139.1, 138.3, 131.8, 131.1, 128.5 (2C), 127.9 (2C), 127.0, 126.3, 124.9, 69.9, 60.8, 42.9, 29.4, 28.4, 27.6, 26.1, 25.8, 25.5, 20.6, 16.4; HRMS (ESI) *m/z*: $[M+H]^+$ calcd for $C_{24}H_{32}N_3O_3$ 410.2444, found 410.2442.	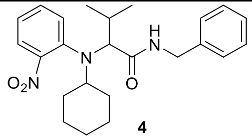

*N-cyclohexyl-2-((2-nitrophenyl)(propyl)amino)butanamide (**5**):* Red- orange oil, 902 mg, yield 52%; ^{1}H NMR (500 MHz, $CDCl_3$): 7.59 (d, 1H, *J* = 8.0 Hz), 7.43 (t, 1H, *J* = 7.6 Hz), 7.25 (d, 1H, *J* = 8.3 Hz), 7.11 (t, 1H, *J* = 7.6 Hz), 6.83 (d, 1H, *J* = 8.7 Hz), 3.62-3.69 (m, 1H), 3.50-3.54 (m, 1H), 2.92-2.99 (m, 1H), 2.71-2.78 (m, 1H), 1.74-1.83 (m, 2H), 1.57-1.66 (m, 5H), 0.97-1.32 (m, 7H), 0.82 (t, 3H, *J* = 7.3 Hz), 0.70 (t, 3H, *J* = 7.3 Hz); ^{13}C{H} NMR (125 MHz, $CDCl_3$): 170.8, 147.0, 142.8, 132.7, 125.5, 124.9, 124.0, 69.0, 52.7, 47.7, 33.0, 32.6, 25.5, 24.72, 24.68, 22.9, 20.2, 11.4, 11.1; HRMS (ESI) *m/z*: $[M+H]^+$ calcd for $C_{19}H_{30}N_3O_3$ 348.2287, found 348.2283.	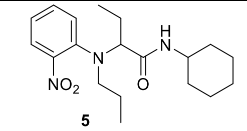 **5**
*2-(4-chlorophenyl)-N-cyclohexyl-2-((4-methoxybenzyl)(4-nitrophenyl)amino)acetamide (**6**):* Yellow solid, 1724 mg, yield 68%; ^{1}H NMR (500 MHz, $CDCl_3$): 8.06 (d, 2H, *J* = 9.3 Hz), 7.27 (d, 2H, *J* = 8.6 Hz), 7.20 (d, 2H, *J* = 8.4 Hz), 7.00 (d, 2H, *J* = 8.5 Hz), 6.79 (d, 4H, *J* = 8.9 Hz), 5.93 (d, 1H, *J* = 8.0 Hz), 5.46 (s, 1H), 4.66 (d, 1H, *J* = 17.1 Hz), 4.50 (d, 1H, *J* = 17.1 Hz), 3.76 (s, 3H), 1.73-1.80 (m, 1H), 1.54-1.63 (m, 2H), 1.43 (s, 2H), 1.20-1.35 (m, 3H), 1.04-1.12 (m, 1H), 0.92-1.00 (m, 2H); ^{13}C{H} NMR (125 MHz, $CDCl_3$): 167.9, 159.1, 153.7, 139.1, 134.8, 133.2, 130.7 (2C), 129.1 (2C), 127.8 (2C), 125.9 (2C), 125.6, 114.4 (2C), 113.2 (2C), 67.4, 55.4, 52.3, 48.8, 32.9, 32.8, 30.5, 25.5, 24.8; HRMS (ESI) *m/z*: $[M+Na]^+$ calcd for $C_{28}H_{30}ClN_3O_4Na$ 530.1817, found 530.1801.	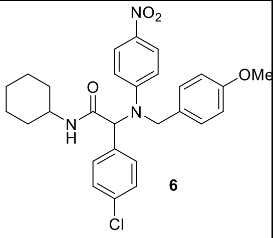 **6**
*N-(4-bromobenzyl)-2-(N-cycloheptylacetamido)-2-(3-nitrophenyl)acetamide (**7**):* Dark brown oil, 1979 mg, yield 79%; ^{1}H NMR (500 MHz, $CDCl_3$): 8.10-8.13 (m, 2H), 7.60 (d, 1H, *J* = 7.7 Hz), 7.48 (t, 1H, *J* = 8.0 Hz), 7.43 (d, 2H, *J* = 8.3 Hz), 7.14 (d, 2H, *J* = 8.3 Hz), 4.41 (d, 2H, *J* = 6.0 Hz), 3.83-3.9 (m, 1H), 2.24 (s, 3H), 1.44-1.97 (m, 12H); ^{13}C{H} NMR (125 MHz, $CDCl_3$): 171.5, 170.3, 148.5, 138.8, 137.2, 133.2, 131.9 (2C), 129.7, 129.5 (2C), 122.8, 122.1, 121.4, 62.4, 53.6, 43.3, 34.1, 33.9, 27.3, 27.2, 25.4, 25.1, 23.1; HRMS (ESI) *m/z*: $[M+H]^+$ calcd for $C_{24}H_{29}BrN_3O_4$ 502.1341, found 502.1335.	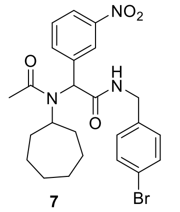 **7**
N-((1s,3s)-adamantan-1-yl)-2-(N-cyclopentylformamido)-2-(4-nitrophenyl)acetamide (8): Pale brown solid, 1594 mg, yield 75%; ^{1}H NMR (500 MHz, $CDCl_3$): 8.37 (s, 1H), 8.19 (d, 2H, *J* = 8.7 Hz), 7.51 (d, 2H, *J* = 8.7 Hz), 6.38 (s, 1H), 5.43 (s, 1H), 3.92-3.98 (m, 1H), 1.99-2.12 (m, 10H), 1.46-1.79 (m, 14H); ^{13}C{H} NMR (125 MHz, $CDCl_3$): 167.7, 163.2, 147.5, 143.1, 128.7 (2C), 123.8 (2C), 61.3, 59.6, 52.7, 41.4 (3C), 36.4 (3C), 32.5, 32.3, 29.5 (3C), 24.2, 24.1; HRMS (ESI) *m/z*: $[M+H]^+$ calcd for $C_{24}H_{32}N_3O_4$ 426.2393, found 426.2385.	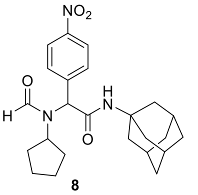 **8**
*N-benzyl-2-(4-chlorophenyl)-N-(2-((2-methoxy-4-nitrophenyl)amino)-2-oxoethyl)acetamide (**9**):* Yellow solid, 1728 mg, yield 74%; ^{1}H NMR (500 MHz, $CDCl_3$): 8.87 (s, 1H), 8.45 (d, 1H, *J* = 8.6 Hz), 7.84 (d, 1H, *J* = 8.6 Hz), 7.69 (s, 1H), 7.34-7.39 (m, 3H), 7.29 (d, 2H, *J* = 8.0 Hz), 7.22 (d, 2H, *J* = 7.3 Hz), 7.17 (d, 2H, *J* = 6.8 Hz), 4.73 (s, 2H), 4.19 (s, 2H), 3.91 (s, 3H), 3.85 (s, 2H); ^{13}C{H} NMR (125 MHz, $CDCl_3$): 172.4, 167.4, 147.5, 143.1, 135.2, 133.5, 133.0, 132.7, 130.3 (2C), 129.1 (2C), 128.8 (2C), 128.2, 126.7 (2C), 118.4, 117.4, 105.2, 56.3,	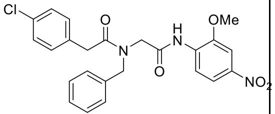 **9**

52.9, 51.5, 39.5; HRMS (ESI) *m/z*: $[M+H]^+$ calcd for $C_{24}H_{23}ClN_3O_5$ 468.1326,found 468.1318.	
*N-(4-methoxybenzyl)-1-(4-nitrophenyl)-1-(1-(2,4,4-trimethylpentan-2-yl)-1H-tetrazol-5-yl)methanamine (**10**):* Pale brown solid, 1943 mg, yield 86%; ^{1}H NMR (500 MHz, $CDCl_3$): 8.22 (d, 2H, *J* = 8.7 Hz), 7.57 (d, 2H, *J* = 8.7 Hz), 7.22 (d, 2H, *J* = 8.6 Hz), 6.87 (d, 2H, *J* = 8.6 Hz), 5.35 (s, 1H), 3.80 (s, 3H), 3.69 (d, 2H, *J* = 6.8 Hz), 1.80 (d, 2H, *J* = 4.4 Hz), 1.66 (s, 3H), 1.63 (s, 3H), 0.64 (s, 9H); ^{13}C{H} NMR (125 MHz, $CDCl_3$): 159.3, 154.9, 147.9, 130.2, 129.72 (2C), 129.65 (2C), 124.3 (2C), 114.2 (2C), 65.4, 56.6, 55.5, 54.0, 50.7, 31.7, 30.7 (3C), 30.5, 30.3; HRMS (ESI) *m/z*: $[M+H]^+$ calcd for $C_{24}H_{33}N_6O_3$ 453.2614, found 453.2607.	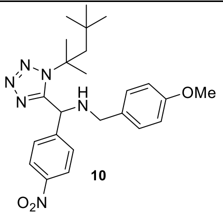 **10**
*N-((1-(tert-butyl)-1H-tetrazol-5-yl)(5-chloro-2-nitrophenyl)methyl)propan-2-amine (**11**):* Pale white solid, 1619 mg, yield 92%; ^{1}H NMR (500 MHz, $CDCl_3$): 7.91 (d, 1H, *J* = 8.6 Hz), 7.44 (dd, 1H, J_1 = 8.6 Hz, J_2 = 1.8 Hz), 7.20 (s, 1H), 6.34 (s, 1H), 2.60-2.67 (m, 1H), 1.74 (s, 9H), 1.04 (d, 3H, *J* = 6.2 Hz), 1.00 (d, 3H, *J* = 6.2 Hz); ^{13}C{H} NMR (125 MHz, $CDCl_3$): 154.4, 147.4, 139.9, 136.3, 129.4, 129.3, 126.9, 62.2, 50.8, 47.0, 30.0 (3C), 22.9, 22.1; HRMS (ESI) *m/z*: $[M+H]^+$ calcd for $C_{15}H_{22}ClN_6O_2$ 353.1493, found 353.1488.	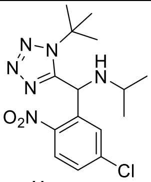 **11**
*Methyl 2-(5-((3-nitrophenyl)(p-tolylamino)methyl)-1H-tetrazol-1-yl)acetate (**12**):* Dark brown oil, 1318 mg, yield 69%; ^{1}H NMR (500 MHz, $CDCl_3$): 8.29 (br t, 1H), 8.14 (d, 1H, *J* = 8.0 Hz), 7.77 (d, 1H, *J* = 7.7 Hz), 7.52 (t, 1H, *J* = 8.0 Hz), 6.95 (d, 2H, *J* = 8.0 Hz),6.55 (d, 2H, *J* = 8.3 Hz), 6.09 (d, 1H, *J* = 7.0 Hz), 5.25 (s, 2H), 3.66 (s, 3H), 2.20 (s, 3H); ^{13}C{H} NMR (125 MHz, $CDCl_3$): 166.1, 155.5, 148.5, 142.4, 139.3, 133.8, 130.2, 130.1 (2C), 129.5, 123.7, 122.6, 114.4 (2C), 53.3, 53.1, 48.9, 20.4; HRMS (ESI) *m/z*: $[M+H]^+$ calcd for $C_{18}H_{19}N_6O_4$ 383.1468, found 383.1463.	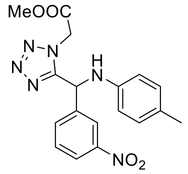 **12**
*Methyl 2-(((1-(tert-butyl)-1H-tetrazol-5-yl)(5-chloro-2-nitrophenyl)methyl) amino)-2-methylpropanoate (**13**):* Orange- brown solid, 1804 mg, yield 88%; ^{1}H NMR (500 MHz, $CDCl_3$): 7.84 (d, 1H, *J* = 8.6 Hz), 7.41 (dd, 1H, J_1 = 8.6 Hz, J_2 = 2.2 Hz), 7.05 (d, 1H, *J* = 2.1 Hz), 6.52 (s, 1H), 3.40 (s, 3H), 1.71 (s, 9H), 1.29 (s, 3H), 1.24 (s, 3H); ^{13}C{H} NMR (125 MHz, $CDCl_3$): 174.9, 154.4, 147.3 139.7, 136.4, 129.7, 129.3, 126.8, 62.2, 57.7, 52.1, 48.2, 30.0 (3C), 26.3, 24.4; HRMS (ESI) *m/z*: $[M+Na]^+$ calcd for $C_{17}H_{23}ClN_6O_4Na$ 403.1620, found 403.1624.	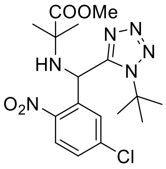 **13**
methyl 3-(((1-(tert-butyl)-1H-tetrazol-5-yl) (4-nitrophenyl) methyl) amino) propanoate (**14**): Yellow- brown solid, 1375 mg, yield 76%; ^{1}H NMR (500 MHz, $CDCl_3$): 8.18 (d, 2H, *J* = 8.6 Hz), 7.53 (d, 2H, *J* = 8.6 Hz), 5.46 (s, 1H), 3.65 (s, 3H), 2.76-2.85 (m, 2H), 2.50 (t, 2H, *J* = 6.3 Hz), 1.68 (s, 9H); ^{13}C{H} NMR (125 MHz, $CDCl_3$): 172.8, 154.7, 147.9, 145.6, 129.2 (2C), 124.2 (2C), 61.8, 58.4, 51.9, 43.4, 34.8, 30.2 (3C); HRMS *m/z* (ESI) calcd for $C_{16}H_{23}N_6O_4$ $(M+H)^+$ 363.1781, found 363.1775.	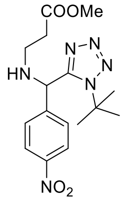 **14**

*Methyl 2-(((1-(tert-butyl)-1H-tetrazol-5-yl)(4-nitrophenyl)methyl)amino)-3-methylbutanoate (**15**):* Yellow oil, 1657 mg, yield 85%; Mainly the one diastereomer in ratio *d.r.* =99/1: ^{1}H NMR (500 MHz, $CDCl_3$): 8.14 (d, 2H, *J* = 8.7 Hz), 7.60 (d, 2H, *J* = 8.7 Hz,), 5.47 (s, 1H), 3.59 (s, 3H), 2.87 (d, 1H, *J* = 5.8 Hz), 1.89-1.93 (m, 1H), 1.67 (s, 9H), 0.86 (d, 3H, *J* = 6.7 Hz), 0.83 (d, 3H, *J*= 6.7 Hz); ^{13}C{H} NMR (125 MHz, $CDCl_3$): 173.9, 154.8, 147.9, 145.4, 129.6 (2C), 123.9 (2C), 65.0, 61.7, 56.6, 51.8, 31.5, 30.1 (3C), 19.2, 18.2; HRMS (ESI) *m/z*: $[M+Na]^+$ calcd for $C_{18}H_{27}N_6O_4Na$ 413.1908, found 413.1897.	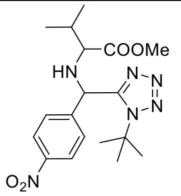 **15** (d.r 99:1)
*Methyl 2-(2-((4-chlorobenzyl)(2-nitrophenyl)amino)acetamido)acetate (**16**):* Brown oil, 1388 mg, yield 71%; ^{1}H NMR (500 MHz, $CDCl_3$): 7.77 (d, 1H, *J* = 8.1 Hz), 7.72 (br t, 1H), 7.48 (t, 1H, *J* = 7.8 Hz), 7.23 (d, 2H, *J* = 8.3 Hz), 7.14-7.19 (m, 2H), 7.03 (d, 2H, *J* = 8.3 Hz), 4.14 (s, 2H), 4.04 (d, 2H, *J* = 5.8 Hz), 3.78 (s, 2H), 3.70 (s, 3H); ^{13}C{H} NMR (125 MHz, $CDCl_3$): 169.9, 169.7, 145.0, 143.6, 134.1, 133.9, 133.7, 130.3 (2C), 128.9 (2C), 125.9, 124.3, 124.2, 59.0, 55.5, 52.4, 41.0; HRMS (ESI) *m/z*: $[M+H]^+$ calcd for $C_{18}H_{19}ClN_3O_5$ 392.1013, found 392.1008.	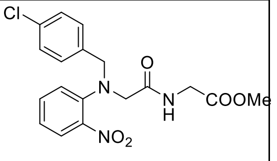 **16**
*Methyl 2-(2-((4-methoxybenzyl)(2-nitrophenyl)amino)acetamido)acetate (**17**):* Orange oil, 1528 mg, yield 79%; ^{1}H NMR (500 MHz, $CDCl_3$): 7.83 (br t, 1H), 7.78 (dd, 1H, J_1 = 8.1 Hz, J_2 = 1.4 Hz), 7.47 (t, 1H, *J* = 7.8 Hz), 7.16 (t, 1H, *J* = 7.8 Hz), 7.13 (d, 1H, *J* = 8.2 Hz), 6.99 (d, 2H, *J* = 8.6 Hz), 6.78 (d, 2H, *J* = 8.6 Hz), 4.09 (s, 2H), 4.04 (d, 2H, *J* = 5.8 Hz), 3.79 (s, 2H), 3.76 (s, 3H), 3.71 (s, 3H); ^{13}C{H} NMR (125 MHz, $CDCl_3$): 170.1, 169.9, 159.5, 144.9, 144.0, 133.7, 130.3 (2C), 127.3, 125.9, 124.2, 124.0, 114.1 (2C), 59.5, 55.3, 54.9, 52.4, 41.1; HRMS (ESI) *m/z*: $[M+H]^+$ calcd for $C_{19}H_{21}N_3O_6$ 388.1509, found 388.1502.	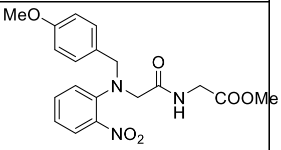 **17**
*Methyl 2-(2-(butyl(2-nitrophenyl)amino)acetamido)acetate (**18**):* Orange oil, 1065 mg, yield 66%; ^{1}H NMR (500 MHz, $CDCl_3$): 7.79 (br s, 1H), 7.74 (d, 1H, *J* = 8.1 Hz), 7.50 (t, 1H, *J* = 7.8 Hz), 7.26 (d, 1H, *J* = 7.1 Hz), 7.15 (t, 1H, *J* = 7.7 Hz), 4.06 (d, 2H, *J* = 5.8 Hz), 3.88 (s, 2H), 3.70 (s, 3H), 3.01 (t, 2H, *J* = 7.7 Hz), 1.37-1.43 (m, 2H), 1.16-1.24 (m, 2H), 0.82 (t, 3H, *J* = 7.3 Hz); ^{13}C{H} NMR (125 MHz, $CDCl_3$): 170.4, 169.9, 145.0, 144.0, 133.7, 125.8, 123.7 (2C), 56.4, 55.6, 52.4, 41.1, 29.1, 20.1, 13.9; HRMS (ESI) *m/z*: $[M+H]^+$ calcd for $C_{15}H_{22}N_3O_5$ 324.1559, found 324.1548.	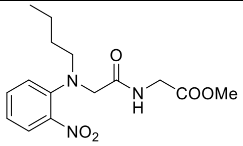 **18**
*Methyl 2-(2-(cyclohexyl(2-nitrophenyl)amino)acetamido)acetate (**19**):* Red oil, 1361 mg, yield 78%; ^{1}H NMR (500 MHz, $CDCl_3$): 8.11 (br s, 1H), 7.73 (d, 1H, *J* = 8.0 Hz), 7.50 (t, 1H, *J* = 7.8 Hz), 7.28 (d, 1H, *J* = 8.2 Hz), 7.18 (t, 1H, *J* = 7.7 Hz), 4.01 (d, 2H, *J* = 5.7 Hz), 3.93 (s, 2H), 3.66 (s, 3H), 2.78-2.83 (m, 1H), 1.83 (d, 2H, *J* = 11.3 Hz), 1.76 (d, 2H, *J* = 12.6 Hz), 1.59 (d, 1H, *J* = 12.9 Hz), 1.13-1.31 (m, 4H), 1.01-1.06 (m, 1H); ^{13}C{H} NMR (125 MHz, $CDCl_3$): 171.4, 169.8, 145.9, 143.6, 133.3, 125.7, 124.6, 124.0, 66.1, 52.2, 50.4, 41.0, 29.7 (2C), 25.8 (2C), 25.5; HRMS (ESI) *m/z*: $[M+H]^+$ calcd for $C_{17}H_{24}N_3O_5$ 350.1716, found 350.1708.	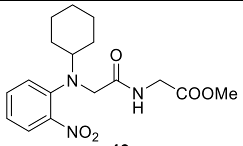 **19**

Methyl 2-(2-(cyclopentyl(2-nitrophenyl)amino)acetamido)acetate (***20***)*:* Orange oil, 1373 mg, yield 82%; ^{1}H NMR (500 MHz, $CDCl_3$): 8.02 (br s, 1H), 7.73 (d, 1H, *J* = 8.0 Hz), 7.50 (t, 1H, *J* = 7.3 Hz), 7.29 (d, 1H, *J* = 8.2 Hz), 7.17 (t, 1H, *J* = 7.7 Hz), 4.01 (d, 2H, *J* = 5.8 Hz), 3.88 (s, 2H), 3.67 (s, 3H), 3.38-3.45 (m, 1H), 1.74-1.77 (m, 2H), 1.62-1.63 (m, 2H), 1.42-1.48 (m, 4H); ^{13}C{H} NMR (125 MHz, $CDCl_3$): 171.2, 169.8, 145.8, 144.6, 133.6, 125.7, 124.6, 124.2, 68.3, 52.3, 52.0, 41.0, 28.9 (2C), 23.5 (2C); HRMS (ESI) *m/z*: $[M+H]^+$ calcd for $C_{16}H_{22}Cl_2N_3O_5$ 336.1559, found 336.1550.	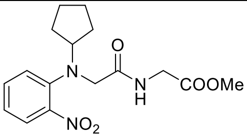 **20**
Methyl 2-(2-(benzo[d][1,3]dioxol-5-yl)-2-((2-nitrophenyl) (3,4,5-trifluorobenzyl)amino) acetamido) acetate (***21***)*:* Orange oil, 1566 mg, yield 59%; ^{1}H NMR (500 MHz, $CDCl_3$): 7.65 (d, 1H, *J*= 8.0 Hz), 7.47 (br s, 1H), 7.38 (t, 1H, *J* = 7.7 Hz), 7.20 (t, 1H, *J* = 7.8 Hz), 7.03 (d, 1H, *J* = 8.1 Hz), 6.93 (s, 1H), 6.83 (d, 1H, *J* = 8.0 Hz), 6.72 (d, 1H, *J* = 8.0 Hz), 6.59 (t, 2H, *J* = 7.1 Hz), 5.93 (s, 2H), 4.86 (s, 1H), 4.24 (d, 1H, *J* = 14.4 Hz), 4.03-4.10 (m, 2H), 3.94 (dd, 1H, J_1 = 18.1 Hz, J_2 = 5.3 Hz), 3.69 (s, 3H); ^{13}C{H} NMR (125 MHz, $CDCl_3$): 170.8, 169.9, 152.0 (ddd, J_1 = 250.3 Hz, J_2 = 10.5 Hz, J_3 = 3.8 Hz), 148.2, 148.1, 147.6, 140.3 (dt, J_1 = 251.8 Hz, J_2 = 15.5 Hz), 132.9, 132.0 (dd, J_1 = 16.7 Hz, J_2 = 6.0 Hz), 128.4, 127.3, 126.1, 125.1, 123.3, 113.5 (dd, J_1 = 16.4 Hz, J_2 = 5.4 Hz), 109.2, 108.5, 101.5, 71.5, 55.5, 52.5, 41.2; ^{19}F (188 MHz, $CDCl_3$): -133.98 (m, 2F), -161.16 (m, 1F); HRMS (ESI) *m/z*: $[M+Na]^+$ calcd for $C_{25}H_{20}F_3N_3O_7Na$ 554.1146, found 554.1132.	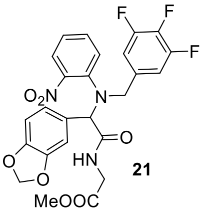 **21**

^{1}H and ^{13}C NMR data for amines 1-21

*2-((4-aminophenyl)(4-methoxybenzyl)amino)-N-(tert-butyl)-2-(4-chlorophenyl) acetamide (**1a**):* Yellow oil, 83 mg, yield 92%; ^{1}H NMR (500 MHz, $CDCl_3$): 7.24-7.28 (m, 4H), 6.88 (d, 2H, *J* = 8.4 Hz), 6.74 (d, 2H, *J* = 8.4 Hz), 6.70 (d, 2H, *J* = 8.5 Hz), 6.53 (d, 2H, *J* = 8.5 Hz), 4.76 (s, 1H), 4.10 (d, 1H, *J* = 14.6 Hz), 3.94 (d, 1H, *J* = 14.6 Hz), 3.76 (s, 3H), 1.20 (s, 9H); ^{13}C{H} NMR (125 MHz, $CDCl_3$): 170.2, 158.8, 141.9, 140.6, 135.4, 133.6, 130.5 (2C), 129.8 (2C), 129.6, 128.6 (2C), 123.7 (2C), 115.9 (2C), 113.6 (2C), 72.0, 55.8, 55.3, 51.0, 28.6 (3C); HRMS (ESI) *m/z*: $[M+H]^+$ calcd for $C_{26}H_{31}ClN_3O_2$ 452.2105, found 452.2100.	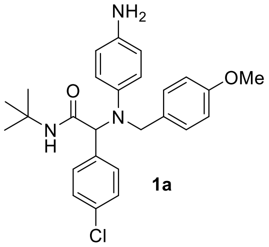 1a
*2-((2-aminophenyl)(4-methoxybenzyl)amino)-N-(tert-butyl)-2-(4-chlorophenyl)acetamide (**2a**):* Yellow oil, 81 mg, yield 90%; ^{1}H NMR (500 MHz, $CDCl_3$): 7.53 (d, 2H, *J* = 7.4 Hz), 7.37 (d, 2H, *J* = 7.6 Hz), 6.99 (d, 1H, *J* = 7.6 Hz), 6.94 (t, 1H, *J* = 7.6 Hz), 6.74 (d, 2H, *J* = 7.8 Hz), 6.68 (d, 3H, *J* = 8.5 Hz), 6.59 (d, 1H, *J* = 7.8 Hz), 6.46 (s, 1H), 4.63 (s, 1H), 3.90 (d, 1H, *J* = 12.3 Hz), 3.77 (d, 1H, *J* = 12.3 Hz), 3.73 (s, 3H),1.02 (s, 9H); ^{13}C{H} NMR (125 MHz, $CDCl_3$): 169.8, 159.1, 143.6, 137.0, 134.8, 133.9, 131.1 (2C), 129.7 (2C), 129.0 (2C), 128.5, 126.6, 125.1, 119.3, 115.6, 113.5 (2C), 73.6, 56.1, 55.3, 51.1, 28.3 (3C); HRMS (ESI) *m/z*: $[M+H]^+$ calcd for $C_{26}H_{31}ClN_3O_2$ 452.2403, found 452.2093.	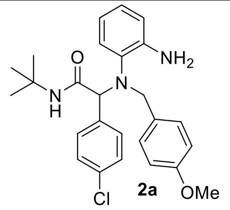 2a
*2-((2-aminophenyl)(3-methoxybenzyl)amino)-N-(tert-butyl)-2-(4-chlorophenyl)acetamide (**3a**):* Yellow oil, 83 mg, yield 92%; ^{1}H NMR (500 MHz, $CDCl_3$): 7.52 (d, 2H, *J* = 8.1 Hz), 7.37 (d, 2H, *J* = 8.1 Hz), 7.08 (t, 1H, *J* = 7.9 Hz), 7.00 (d, 1H, *J* = 7.6 Hz), 6.94 (t, 1H, *J* = 7.8 Hz), 6.74 (dd, 1H, J_1 = 8.2 Hz, J_2 = 2.4 Hz), 6.69 (t, 1H, *J* = 7.5 Hz), 6.60 (d, 1H, *J* = 7.8 Hz), 6.48 (m, 2H), 6.31 (br s, 1H), 4.65 (s, 1H), 3.92 (d, 1H, *J* = 12.8 Hz), 3.71 (d, 1H, *J* = 14.3 Hz), 3.61 (s, 3H), 1.04 (s, 9H); ^{13}C{H} NMR (125 MHz, $CDCl_3$): 169.8, 159.3, 143.5, 138.0, 136.8, 134.8, 134.0, 129.8 (2C), 129.1, 129.0 (2C), 126.7, 125.0, 122.1, 119.2, 115.6, 115.2, 113.4, 56.5, 55.1, 51.1, 50.8, 28.3 (3C); HRMS (ESI) *m/z*: $[M+Na]^+$ calcd for $C_{26}H_{30}ClN_3O_2Na$ 474.1924, found 474.1920.	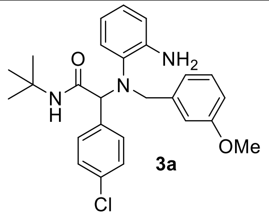 3a
*N-benzyl-2-(cyclohexyl(2-nitrophenyl)amino)-3-methylbutanamide (**4a**):* Yellow oil, 69 mg, yield 91%; ^{1}H NMR (500 MHz, $CDCl_3$): 7.20-7.24 (m, 2H), 7.13 (m, 2H), 7.02 (t, 1H, *J* = 6.3 Hz), 6.87 (t, 1H, *J* = 7.6 Hz), 6.82 (d, 1H, *J* = 7.5 Hz), 6.74 (t, 1H, *J* = 7.5 Hz), 6.62 (d, 1H, *J* = 7.8 Hz), 4.51 (dd, 1H, J_1 = 14.9 Hz, J_2 = 6.3 Hz), 4.32 (dd, 1H, J_1 = 14.9 Hz, J_2 = 5.6 Hz), 3.64 (d, 1H, *J* = 4.7 Hz), 2.33-2.40 (m, 1H), 1.06 (m, 6H); ^{13}C{H} NMR (125 MHz, $CDCl_3$): 172.9, 138.4, 137.7, 128.7 (2C), 127.8 (2C), 127.4, 122.8, 120.0, 118.7, 113.5, 65.2, 43.2, 31.6, 19.9, 18.2; HRMS (ESI) *m/z*: $[M+H]^+$ calcd for $C_{24}H_{34}N_3O$ 380.2702, found 380.2695.	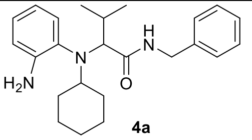 4a
*2-((2-aminophenyl)(propyl)amino)-N-cyclohexylbutanamide (**5a**):* Yellow oil, 57 mg, yield 90%; ^{1}H NMR (500 MHz, $CDCl_3$): 7.05 (d, 1H, *J* = 7.8 Hz), 6.95 (t, 1H, *J* = 7.5 Hz), 6.70-6.74 (m, 2H), 5.95 (br s, 1H), 4.12 (br s, 2H), 3.75 (m, 1H), 3.34 (br t, 1H), 2.96 (q, 1H, *J* = 7.2 Hz), 1.85 (d, 1H, *J* = 11.0 Hz), 1.56-1.76 (m, 6H), 1.33 (m, 4H, *J* = 12.0 Hz), 1.00-1.14 (m, 4H), 0.89 (t, 3H, *J* = 7.4 Hz), 0.80 (t, 3H, *J*= 7.3 H); ^{13}C{H} NMR (125 MHz, $CDCl_3$): 171.3, 143.6, 135.1, 125.8,	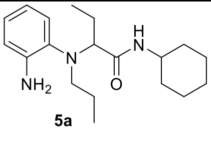 5a

124.5, 118.7, 115.8, 68.8, 52.1, 47.8, 33.4, 33.0, 25.6, 24.95, 24.92, 23.3, 20.5, 11.9, 10.6; HRMS (ESI) *m/z*: $[M+H]^+$ calcd for $C_{19}H_{32}N_3O$ 318.2545, found 318.2538.	
*2-((4-aminophenyl)(4-methoxybenzyl)amino)-2-(4-chlorophenyl)-N-cyclohexylacetamide (**6a**):* Yellow oil, 88 mg, yield 92%; ^1H NMR (500 MHz, $CDCl_3$): 6.86 (d, 3H, *J* = 8.0 Hz), 6.69-6.77 (m, 7H), 6.53 (d, 2H, *J* = 7.9 Hz), 4.83 (s, 1H), 4.09 (d, 1H, *J* = 14.5 Hz), 3.95 (d, 1H, *J* = 14.5 Hz), 3.76 (s, 3H), 3.65-3.69 (m, 1H), 1.53-1.76 (m, 4H), 1.44 (s, 1H), 1.22-1.34 (m, 3H), 1.12-1.17 (m, 1H), 0.92-1.06 (m, 1H); $^{13}C\{H\}$ NMR (125 MHz, $CDCl_3$): 170.1, 158.8, 142.0, 140.5, 135.4, 133.7, 130.5 (2C), 129.9 (2C), 129.4, 128.6 (2C), 123.7 (2C), 115.9 (2C), 113.6 (2C), 71.2, 55.9, 55.3, 50.9, 47.8, 33.0, 30.4, 25.6, 24.7; HRMS (ESI) *m/z*: $[M+H]^+$ calcd for $C_{28}H_{33}ClN_3O_2$ 478.2261, found 478.2254.	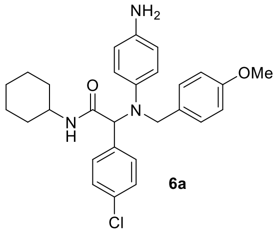**6a**
*2-(3-aminophenyl)-N-(4-bromobenzyl)-2-(N-cycloheptylacetamido)acetamide (**7a**):* Yellow oil, 78 mg, yield 84%; ^1H NMR (500 MHz, $CDCl_3$): 7.39 (d, 2H, *J* = 8.2 Hz), 7.13 (d, 2H, *J* = 8.2 Hz), 7.09 (t, 1H, *J* = 7.8 Hz), 6.72 (s, 1H), 6.68 (d, 1H, *J* = 7.5 Hz), 6.59 (d, 1H, *J* = 7.8 Hz), 6.46 (t, 1H, *J* = 6.4 Hz), 4.71 (br s, 2H), 4.40 (dd, 1H, J_1 = 15.3 Hz, J_2 = 6.2 Hz), 4.30 (dd, 1H, J_1 = 15.3 Hz, J_2 = 5.6 Hz), 3.79 (br t, 1H, *J* = 9.9 Hz), 2.21 (s, 3H), 2.10-2.16 (m, 1H), 1.91-1.93 (m, 1H), 1.74-1.76 (m, 1H), 1.45-1.63 (m, 9H), 1.32-1.34 (m, 1H); $^{13}C\{H\}$ NMR (125 MHz, $CDCl_3$): 170.72, 170.65, 170.57, 147.1, 138.5, 137.7, 137.7, 131.6 (2C), 129.8, 129.3 (2C), 125.6, 121.0, 118.5, 114.9, 114.6, 63.5, 61.8, 43.1, 43.0, 33.9, 33.7, 30.4, 29.8, 27.6, 27.5, 25.6, 25.5, 22.9; HRMS (ESI) *m/z*: $[M+H]^+$ calcd for $C_{24}H_{31}BrN_3O_2$ 472.1600, found 472.1593.	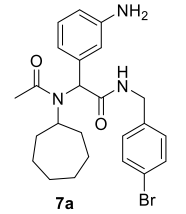**7a**
*N-((1s,3s)-adamantan-1-yl)-2-(4-aminophenyl)-2-(N-cyclopentylformamido)acetamide (**8a**):* Yellow oil, 69 mg, yield 88%; Mixture of topoisomers a and b in ratio of a/b=3/1: ^1H NMR (500 MHz, $CDCl_3$): 8.34 (s, 1H, a), 8.06 (s, 1H, b), 7.13 (d, 2H, *J* = 8.1 Hz, a), 7.03 (d, 2H, *J* = 8.1 Hz, b), 6.65 (d, 4H, *J* = 8.1 Hz, a and b), 5.68 (s, 1H, a), 5.52 (s, 1H, b), 5.43 (s, 1H, a), 4.81 (s, 1H, b), 4.47 (q, 1H, *J*= 8.3 Hz, b), 3.82 (q, 1H, *J* = 8.4 Hz, a), 3.46 (s, 1H, a), 2.21 (s, 1H, b), 2.10-2.15 (m, 1H, a and b), 2.05 (d, 8H, *J* = 17.8 Hz, b), 1.98 (d, 8H, *J* = 12.0 Hz, a), 1.75-1.81 (m, 4H, a and b), 1.65 (d, 16H, *J* = 12.2 Hz, a and b), 1.50-1.58 (m, 4H, a and b), 1.40-1.44 (m, 2H, a and b), 1.24-1.32 (m, 4H, a and b); $^{13}C\{H\}$ NMR (125 MHz, $CDCl_3$): 168.9 (b), 168.8 (a), 164.1 (b), 162.7 (a), 146.8 (b), 146.5 (a), 129.9 (2C, a), 129.5 (2C, b), 125.7 (b), 125.5 (a), 115.4 (2C, b), 115.2 (2C, a), 62.9 (a and b), 60.5 (a), 58.5 (a), 54.8 (b), 52.7 (b), 52.3 (a), 41.5 (3C, b), 41.4 (3C, a), 36.4 (3C, a), 33.1 (a), 32.7 (a), 29.5 (3C, a and b), 24.3 (a), 36.3 (3C, b), 30.8 (b), 30.4 (b), 29.1 (b), 24.2 (2C, a and b), 24.1 (b); HRMS (ESI) *m/z*: $[M+H]^+$ calcd for $C_{24}H_{34}N_3O_2$ 396.2651, found 396.2647.	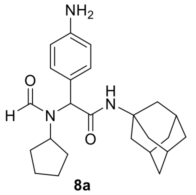**8a**
*N-(2-((4-amino-2-methoxyphenyl)amino)-2-oxoethyl)-N-benzyl-2-(4-chlorophenyl) acetamide (**9a**):* Yellow oil, 79 mg, yield 90%; ^1H NMR (500 MHz, $CDCl_3$): 8.17 (s, 1H), 7.98 (d, 1H, *J* = 8.2 Hz), 7.35 (t, 2H, *J* = 8.2 7.4 Hz), 7.24-7.27 (m, 3H), 7.19 (d, 2H, *J* = 8.2 Hz), 7.14 (d, 2H, *J* = 7.2 Hz), 6.31 (d, 2H, *J* = 10.0 Hz), 4.69 (s, 2H), 3.99 (br s, 1H), 3.78 (s, 2H), 3.73-3.75 (m, 5H); $^{13}C\{H\}$ NMR (125 MHz, $CDCl_3$): 172.1, 171.8, 166.1, 149.6, 143.5, 135.7, 133.1, 130.4	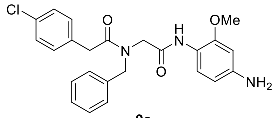**9a**

(2C), 129.2 (2C), 129.0 (2C), 128.1, 126.7 (2C), 121.7, 119.1, 107.0, 98.5, 55.7, 52.7, 51.1, 39.8; HRMS (ESI) *m/z*: $[M+H]^+$ calcd for $C_{24}H_{25}ClN_3O_3$ 438.1584, found 438.1580.	
*4-(((4-methoxybenzyl)amino)(1-(2,4,4-trimethylpentan-2-yl)-1H-tetrazol-5-yl)methyl) aniline (**10a**):* Yellow oil, 78 mg, yield 92%; ^1H NMR (500 MHz, $CDCl_3$): 7.24 (d, 2H, *J* = 8.3 Hz), 7.10 (d, 2H, *J* = 8.2 Hz), 6.85 (d, 2H, *J* = 8.3 Hz), 6.63 (d, 2H, *J* = 8.2 Hz), 5.14 (s, 1H), 3.79 (s, 3H), 3.68 (d, 2H, *J* = 5.5 Hz), 3.30 (br s, 2H), 1.79 (d, 1H, *J* = 15.0 Hz), 1.72 (d, 1H, *J* = 15.1 Hz), 1.62 (s, 3H), 1.59 (s, 3H), 0.61 (s, 9H); $^{13}C\{H\}$ NMR (125 MHz, $CDCl_3$): 159.0, 156.1, 146.7, 131.2, 129.7 (4C), 128.1, 115.4 (2C), 114.4 , 114.0 (2C), 65.1, 56.9, 55.4, 53.5, 50.4, 31.5, 30.6 (3C), 30.5, 30.0; HRMS (ESI) *m/z*: $[M+H]^+$ calcd for $C_{24}H_{36}N_6O$ 423.2872, found 423.2868.	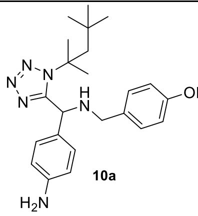 10a
*2-((1-(tert-butyl)-1H-tetrazol-5-yl)(isopropylamino)methyl)-4-chloroaniline (**11a**):* Light yellow solid, 60 mg, yield 94%; ^1H NMR (500 MHz, $CDCl_3$): 7.05 (dd, 1H, J_1 = 8.4 Hz, J_2 = 2.5 Hz), 6.68 (d, 1H, *J* = 8.4 Hz), 6.21 (d, 1H, *J* = 2.6 Hz), 5.45 (s, 1H), 2.62-2.70 (m, 1H), 1.59 (s, 9H), 1.13 (d, 3H, *J* = 6.1 Hz), 1.07 (d, 3H, *J* = 6.2 Hz); $^{13}C\{H\}$ NMR (125 MHz, $CDCl_3$): 154.7, 144.2, 129.1, 127.4, 126.2, 123.5, 118.4, 61.7, 52.8, 46.7, 30.0 (3C), 23.8, 21.6; HRMS (ESI) *m/z*: $[M+H]^+$ calcd for $C_{15}H_{24}ClN_6$ 323.1751, found 323.1745.	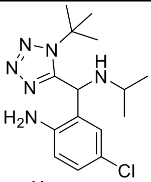 11a
*Methyl 2-(5-((3-aminophenyl)(p-tolylamino)methyl)-1H-tetrazol-1-yl)acetate (**12a**):* Yellow oil, 62 mg, yield 90%; ^1H NMR (500 MHz, $CDCl_3$): 7.10 (t, 1H, *J* = 7.7 Hz), 6.95 (d, 2H, *J* = 7.5 Hz), 6.67 (d, 1H, *J* = 7.5 Hz), 6.60 (d, 2H, *J* = 11.0 Hz), 6.54 (d, 2H, *J* = 8.1 Hz), 5.84 (s, 1H), 5.12 (d, 1H, *J* = 17.6 Hz), 5.03 (d, 1H, *J* = 17.6 Hz), 3.56 (s, 3H), 2.20 (s, 3H); $^{13}C\{H\}$ NMR (125 MHz, $CDCl_3$): 166.0, 156.2, 147.4, 143.6, 138.0, 130.3, 129.9, 128.8, 117.0, 115.7, 114.2, 113.5, 54.5, 53.1, 48.7, 20.5; HRMS (ESI) *m/z*: $[M+H]^+$ calcd for $C_{18}H_{21}N_6O_2$ 353.1726, found 353.1719.	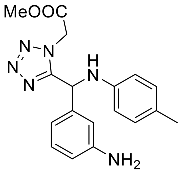 12a
*Methyl 2-(((2-amino-5-chlorophenyl)(1-(tert-butyl)-1H-tetrazol-5-yl)methyl) amino)-2-methylpropanoate (**13a**):* Light yellow solid, 71 mg, yield 92%; ^1H NMR (500 MHz, $CDCl_3$): 7.35 (d, 1H, *J* = 8.5 Hz), 7.24 (d, 1H, *J* = 8.6 Hz), 6.41 (s, 1H), 5.60 (s, 1H), 3.58 (s, 3H), 1.58 (s, 9H), 1.44 (s, 3H), 1.20 (s, 3H); $^{13}C\{H\}$ NMR (125 MHz, $CDCl_3$): 176.2, 155.3, 146.5, 129.4, 127.3, 126.6, 126.4, 116.1, 61.9, 58.5, 52.5, 49.6, 29.9 (3C), 26.14, 26.08; HRMS (ESI) *m/z*: $[M+Na]^+$ calcd for $C_{17}H_{25}ClN_6O_2Na$ 403.1620, found 403.1624.	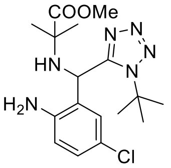 13a
*Methyl 3-(((4-aminophenyl)(1-(tert-butyl)-1H-tetrazol-5-yl)methyl)amino) propanoate (**14a**):* Light yellow solid, 59 mg, yield 90%; ^1H NMR (500 MHz, $CDCl_3$): 6.98 (d, 2H, *J* = 8.2 Hz), 6.56 (d, 2H, *J* = 8.3 Hz), 5.17 (s, 1H), 3.60 (s, 3H), 2.71-2.81 (m, 2H), 2.45 (t, 2H, *J* = 6.7 Hz), 1.57 (s, 9H); $^{13}C\{H\}$ NMR (125 MHz, $CDCl_3$): 172.8, 155.9, 146.7, 129.1 (2C), 127.9, 115.2 (2C), 61.3, 58.6, 51.6, 43.1, 34.8, 29.9 (3C); HRMS (ESI) *m/z*: $[M+Na]^+$ calcd for $C_{16}H_{24}N_6O_2Na$ 355.1853, found 355.1852.	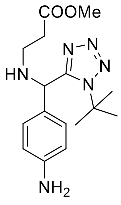 14a

*Methyl 2-(((4-aminophenyl)(1-(tert-butyl)-1H-tetrazol-5-yl)methyl)amino)-3-methylbutanoate (**15a**):* Light yellow solid, 67 mg, yield 93%; Mainly the one diastereomer in ratio *d.r.* =99/1; ^{1}H NMR (500 MHz, $CDCl_3$): 7.11 (d, 2H, *J* = 8.3 Hz), 6.62 (d, 2H, *J* = 8.2 Hz), 5.25 (s, 1H), 3.66 (s, 3H), 2.97 (d, 1H, *J* = 5.9 Hz), 1.91-1.98 (m, 1H), 1.63 (s, 9H), 0.91 (d, 3H, *J* = 6.8 Hz), 0.87 (d, 3H, *J* = 6.8 Hz); ^{13}C{H} NMR (125 MHz, $CDCl_3$): 174.5, 156.2, 146.6, 129.9 (2C), 127.6, 115.3 (2C), 64.58, 61.41, 56.93, 51.71, 31.6, 30.1 (3C), 19.3, 18.6; HRMS (ESI) *m/z*: $[M+Na]^+$ calcd for $C_{18}H_{28}N_6O_2Na$ 383.2166, found 383.2166.	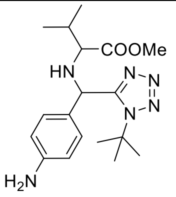<br>**15a** (d.r 99:1)
*Methyl 2-(2-((2-aminophenyl)(4-chlorobenzyl)amino)acetamido)acetate (**16a**):* Pale red oil, 66 mg, yield 95%; ^{1}H NMR (500 MHz, $CDCl_3$): 7.24 (d, 2H, *J* = 8.2 Hz), 7.11 (d, 2H, *J* = 8.1 Hz), 6.96 (t, 1H, *J* = 7.3 Hz), 6.92 (d, 1H, *J* = 7.8 Hz), 6.68-6.73 (m, 2H), 4.08 (s, 2H), 3.98 (d, 2H, *J* = 5.2 Hz), 3.73 (s, 3H), 3.67 (s, 2H); ^{13}C{H} NMR (125 MHz, $CDCl_3$): 170.49, 170.47, 141.9, 135.9, 135.1, 133.6, 130.7 (2C), 128.7 (2C), 126.2, 123.0, 119.1, 116.2, 58.1, 56.9, 52.5, 41.0; HRMS (ESI) *m/z*: $[M+H]^+$ calcd for $C_{18}H_{21}ClN_3O_3$ 362.1271, found 362.1268.	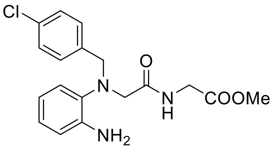<br>**16a**
*Methyl 2-(2-((2-aminophenyl)(4-methoxybenzyl)amino)acetamido)acetate (**17a**):* Pale red oil, 68 mg, yield 96%; ^{1}H NMR (500 MHz, $CDCl_3$): 7.31 (br t, 1H), 7.10 (d, 2H, *J* = 8.2 Hz), 6.94- 6.97 (m, 2H), 6.81 (d, 2H, *J* = 8.1 Hz), 6.70- 6.74 (m, 2H), 4.02 (s, 2H), 3.96 (d, 2H, *J* = 5.1 Hz), 3.78 (s, 3H), 3.73 (s, 3H), 3.69 (s, 2H); ^{13}C{H} NMR (125 MHz, $CDCl_3$): 170.7, 170.5, 159.2, 142.1, 136.5, 130.7 (2C), 128.8, 126.1, 123.2, 119.1, 116.1, 113.9 (2C), 58.8, 56.9, 55.4, 52.5, 41.1; HRMS (ESI) *m/z*: $[M+H]^+$ calcd for $C_{19}H_{24}N_3O_4$ 358.1767, found 358.0937.	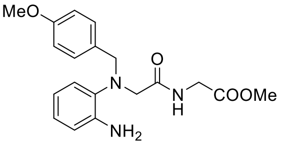<br>**17a**
*Methyl 2-(2-((2-aminophenyl)(butyl)amino)acetamido)acetate (**18a**):* Pale red oil, 55 mg, yield 93%; ^{1}H NMR (500 MHz, $CDCl_3$): 7.44 (br s, 1H), 7.06 (d, 1H, *J* = 8.2 Hz), 6.96 (t, 1H, *J* = 7.6 Hz), 6.71- 6.75 (m, 2H), 4.06 (d, 2H, *J* = 5.3 Hz), 3.74 (s, 2H), 3.65 (s, 3H), 2.95 (t, 2H, *J* = 7.7 Hz), 1.42-1.47 (m, 2H), 1.27-1.37 (m, 2H), 0.87 (t, 3H, *J*= 7.3 Hz); ^{13}C{H} NMR (125 MHz, $CDCl_3$): 171.2, 170.6, 142.4, 136.6, 125.9, 122.7, 119.0, 116.1, 58.5, 55.1, 52.4, 41.0, 29.5, 20.5, 14.1; HRMS (ESI) *m/z*: $[M+Na]^+$ calcd for $C_{15}H_{24}N_3O_3Na$ 316.1632, found 316.1615.	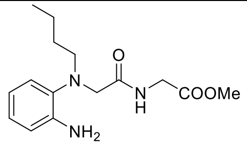<br>**18a**
*Methyl 2-(2-((2-aminophenyl)(cyclohexyl)amino)acetamido)acetate (**19a**):* Pale red oil, 60 mg, yield 95%; ^{1}H NMR (500 MHz, $CDCl_3$): 7.53 (br s, 1H), 7.04 (d, 1H, *J* = 7.8 Hz), 6.96 (t, 1H, *J* = 7.6 Hz), 6.71- 6.76 (m, 2H), 3.97 (d, 2H, *J* = 5.1 Hz), 3.78 (s, 2H), 3.70 (s, 3H), 2.73-2.78 (m, 1H), 1.90 (d, 2H, *J* = 11.4 Hz), 1.77 (d, 2H, *J* = 12.9 Hz), 1.60 (d, 1H, *J* = 12.6 Hz),1.15-1.35 (m, 4H), 1.04-1.09 (m, 1H); ^{13}C{H} NMR (125 MHz, $CDCl_3$): 172.0, 170.5, 142.9, 136.1, 125.9, 125.0, 118.9, 116.2, 62.4, 53.1, 52.4, 41.1, 30.2 (2C), 25.9 (3C); HRMS (ESI) *m/z*: $[M+Na]^+$ calcd for $C_{17}H_{26}N_3O_3Na$ 342.1788, found 342.1771.	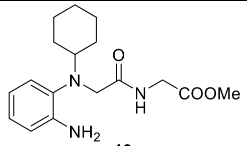<br>**19a**
*Methyl 2-(2-((2-aminophenyl)(cyclopentyl)amino)acetamido)acetate (**20a**):* Pale red oil, 57 mg, yield 94%; ^{1}H NMR (500 MHz, $CDCl_3$): 7.57 (br s, 1H), 7.10 (d, 1H, *J* = 8.1 Hz), 6.97 (t, 1H, *J* = 7.5 Hz), 6.73- 6.74 (m, 2H), 3.98 (d, 2H, *J* = 5.2 Hz), 3.75 (s, 2H), 3.71 (s, 3H), 3.43-3.47 (m, 1H), 1.73-1.77 (m, 2H), 1.62-1.65 (m, 2H), 1.45-1.53 (m, 4H); ^{13}C{H} NMR (125 MHz, $CDCl_3$): 171.7, 170.5,	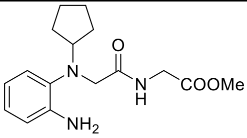<br>**20a**<br>Exact Mass: 305.17

143.3, 137.2, 126.3, 124.9, 119.3, 116.1, 66.3, 56.4, 52.4, 41.1, 29.8 (2C), 23.8 (2C); HRMS (ESI) *m/z*: $[M+Na]^+$ calcd for $C_{16}H_{24}N_3O_3$ 328.1632, found 328.1614.	
*Methyl 2-(2-((2-aminophenyl)(3,4,5-trifluorobenzyl)amino)-2-(benzo[d][1,3]dioxol-5-yl)acetamido)acetate (**21a**):* Pale red oil, 90 mg, yield 90%; ^{1}H NMR (500 MHz, $CDCl_3$): 6.89-6.93 (m, 2H), 6.84 (d, 1H, *J* = 7.8 Hz), 6.75-6.79 (m, 3H), 6.65-6.70 (m, 3H), 6.60 (t, 1H, *J* = 7.5 Hz), 5.97 (s, 2H), 4.74 (s, 1H), 4.16 (d, 1H, *J* = 13.6 Hz), 3.93-4.00 (m, 2H), 3.87 (d, 1H, *J* = 13.7 Hz), 3.71 (s, 3H); ^{13}C{H} NMR (125 MHz, $CDCl_3$): 171.4, 170.3, 151.8 (ddd, J_1 = 250.0 Hz, J_2 = 10.2 Hz, J_3 = 2.8 Hz), 148.3, 148.1, 142.9, 140.0 (dt, J_1 = 250.2 Hz, J_2 = 14.5 Hz), 134.0 (dd, J_1 = 17.2 Hz, J_2 = 5.6 Hz), 133.5, 130.5, 129.5, 126.4, 124.4, 119.7, 113.5 (dd, J_1 = 16.7 Hz, J_2 = 4.5 Hz), 109.1, 108.5, 101.4, 70.8, 66.2, 52.6, 41.3; HRMS (ESI) *m/z*: $[M+Na]^+$ calcd for $C_{25}H_{23}F_3N_3O_5Na$ 524.1404, found 524.1374.	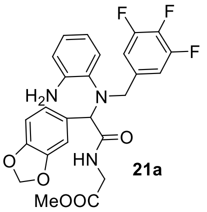 **21a**

^{1}H and ^{13}C NMR data for dihydroquinoxalin-2-ones 16b-21b

*4-(4-chlorobenzyl)-3,4-dihydroquinoxalin-2(1H)-one (**16b**):* Yellow oil, 48 mg, yield 89%; ^{1}H NMR (500 MHz, $CDCl_3$): 8.57 (br s, 1H), 7.32 (d, 2H, *J* = 8.3 Hz,), 7.25 (d, 2H, *J* = 8.3 Hz), 6.92-6.95 (m, 1H), 6.79 (d, 2H, *J* = 4.1 Hz), 6.70, (d, 1H, *J* = 8.0 Hz), 4.37 (s, 2H), 3.80 (s, 2H); ^{13}C{H} NMR (125 MHz, $CDCl_3$): 166.9, 135.1, 135.0, 133.5, 129.2 (2C), 129.0 (2C), 126.3, 124.4, 119.5, 115.8, 112.5, 53.2, 52.5; HRMS (ESI) *m/z*: $[M+H]^+$ calcd for $C_{15}H_{14}ClN_2O$ 273.0795, found 273.0788.	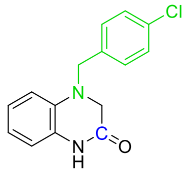 **16b**
*4-(4-methoxybenzyl)-3,4-dihydroquinoxalin-2(1H)-one (**17b**):* Yellow oil, 48 mg, yield 90%; ^{1}H NMR (500 MHz, $CDCl_3$): 9.11 (br s, 1H), 7.23 (d, 2H, *J* = 8.2 Hz), 6.95 (t, 1H, *J* = 7.6 Hz), 6.88 (d, 2H, *J* = 8.2 Hz), 6.74-6.80 (m, 3H), 4.34 (s, 2H), 3.80 (s, 3H), 3.76 (s, 2H); ^{13}C{H} NMR (125 MHz, $CDCl_3$): 167.5, 159.2, 135.5, 129.1 (2C), 128.2, 126.3, 124.3, 119.1, 115.8, 114.3 (2C), 112.3, 55.4, 53.0, 51.9; HRMS (ESI) *m/z*: $[M+H]^+$ calcd for $C_{16}H_{17}N_2O_2$ 269.1290, found 269.1284.	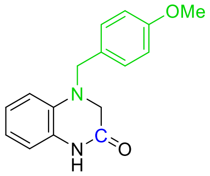 **17b**
*4-butyl-3,4-dihydroquinoxalin-2(1H)-one (**18b**):* Yellow oil, 35 mg, yield 85%; ^{1}H NMR (500 MHz, $CDCl_3$): 9.18 (br s, 1H), 6.97 (t, 1H, *J* = 7.5 Hz), 6.78 (d, 1H, *J* = 7.5 Hz), 6.72 (t, 1H, *J* = 7.5 Hz), 6.69 (d, 1H, *J* = 8.2 Hz), 3.86 (s, 2H), 3.22 (t, 2H, *J*= 7.4 Hz), 1.59-1.65 (m, 2H), 1.37-1.45 (m, 2H), 0.98 (t, 3H, *J* = 7.4 Hz); ^{13}C{H} NMR (125 MHz, $CDCl_3$): 167.4, 135.2, 126.2, 124.3, 118.4, 115.8, 111.7, 52.3, 49.5, 27.3, 20.4, 14.0; HRMS (ESI) *m/z*: $[M+Na]^+$ calcd for $C_{12}H_{16}N_2ONa$ 227.1155, found 227.1147.	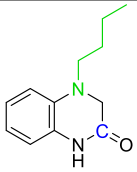 **18b**
*4-cyclohexyl-3,4-dihydroquinoxalin-2(1H)-one (**19b**):* Yellow oil, 41 mg, yield 91%; ^{1}H NMR (500 MHz, $CDCl_3$): 7.37 (d, 1H, *J* = 7.4 Hz), 7.02 (t, 1H, *J* = 7.7 Hz), 6.84 (t, 1H, *J* = 7.3 Hz), 6.72 (d, 1H,*J* = 8.0 Hz), 3.89 (s, 2H), 3.46 (t, 1H, *J* = 9.4 Hz), 1.88 (d, 2H, *J* = 11.4 Hz), 1.82 (d, 2H, *J* = 10.2 Hz), 1.72 (d, 1H, *J* = 13.1 Hz), 1.35-1.47 (m, 4H), 1.11-1.18 (m, 1H); ^{13}C{H} NMR (125 MHz, $CDCl_3$): 161.4, 135.3, 127.4, 124.9, 118.7, 113.8, 112.1, 56.3, 46.4, 28.8 (2C), 26.0 (2C), 25.9; HRMS (ESI) *m/z*: $[M+H]^+$ calcd for $C_{14}H_{19}N_2O$ 231.1497, found 231.1492.	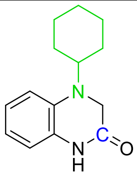 **19b**
*4-cyclopentyl-3,4-dihydroquinoxalin-2(1H)-one (**20b**):* Yellow oil, 38 mg, yield 88%; ^{1}H NMR (500 MHz, $CDCl_3$): 8.40 (br s, 1H), 6.98-7.01 (m, 1H), 6.83 (d, 1H, *J* = 8.0 Hz), 6.75-6.79 (m, 2H), 4.09-4.15 (m, 1H), 3.72 (s, 2H), 1.95-1.97 (m, 2H), 1.73-1.75 (m, 2H), 1.60-1.69 (m, 4H); ^{13}C{H} NMR (125 MHz, $CDCl_3$): 168.1, 136.2, 127.1, 124.2, 119.2, 115.7, 113.5, 58.4, 46.7, 27.8 (2C), 24.8 (2C); HRMS (ESI) *m/z*: $[M+H]^+$ calcd for $C_{13}H_{17}N_2O$ 217.1341, found 217.1334.	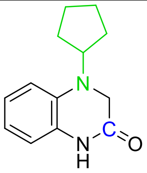 **20b**

*3-(benzo[d][1,3]dioxol-5-yl)-4-(3,4,5-trifluorobenzyl)-3,4-dihydroquinoxalin-2(1H)-one (**21b**):* Yellow oil, 69 mg, yield 84%; ^{1}H NMR (500 MHz, $CDCl_3$): 8.67 (br s, 1H), 6.89-6.96 (m, 3H), 6.80-6.81 (m, 2H), 6.71 (d, 1H, *J* = 7.6 Hz), 6.64-6.67 (m, 2H), 6.56 (d, 1H, *J* = 8.0 Hz), 5.91 (s, 2H), 4.86 (s, 1H), 4.51 (d, 1H, *J* = 16.0 Hz), 4.10 (d, 1H, *J* = 16.0 Hz); ^{13}C{H} NMR (125 MHz, $CDCl_3$): 166.5, 152.7 (ddd, J_1 = 251.0 Hz, J_2 = 10.0 Hz, J_3 = 3.9 Hz), 148.4, 148.2, 140.2 (dt, J_1 = 251.3 Hz, J_2 = 15.2 Hz), 133.3 (dd, J_1 = 6.4 Hz, J_2 = 2.3 Hz), 133.2, 130.5, 125.4, 124.8, 121.0, 119.8, 115.9, 112.5, 111.3 (dd, J_1 = 16.5 Hz, J_2 = 5.1 Hz), 108.7, 107.4, 101.4, 66.1, 51.2; HRMS (ESI) *m/z*: $[M+H]^+$ calcd for $C_{22}H_{16}F_3N_2O_3$ 413.1113, found 413.1107.	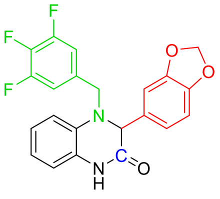 **21b**

Copies of ^{1}H and ^{13}C NMR spectra of nitro compounds 1-21

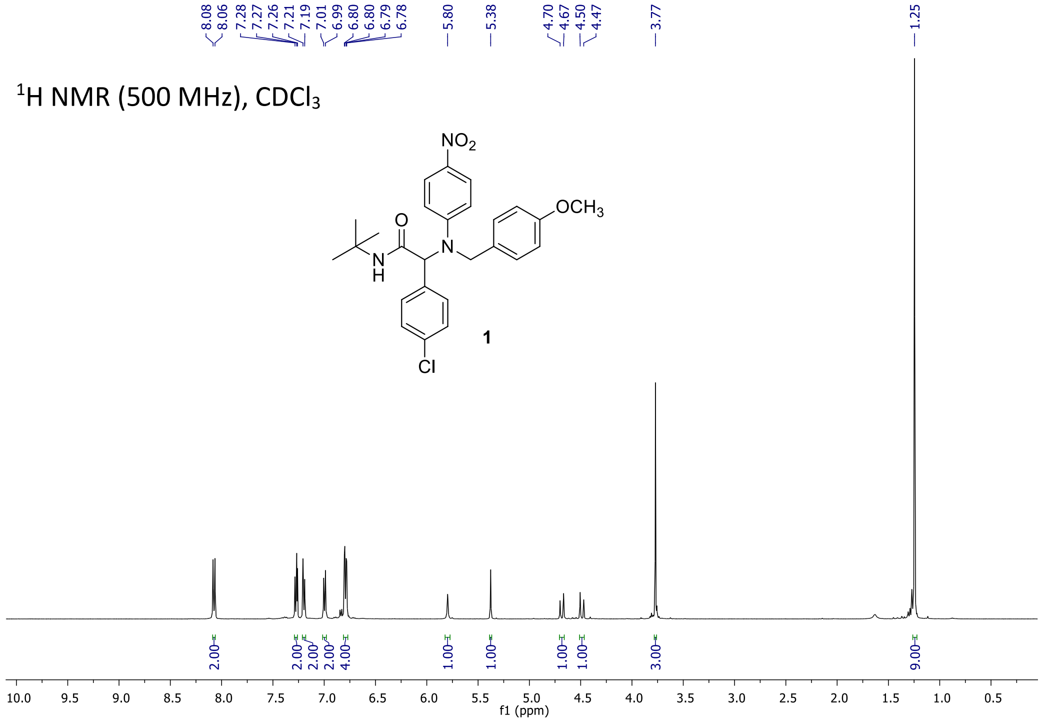

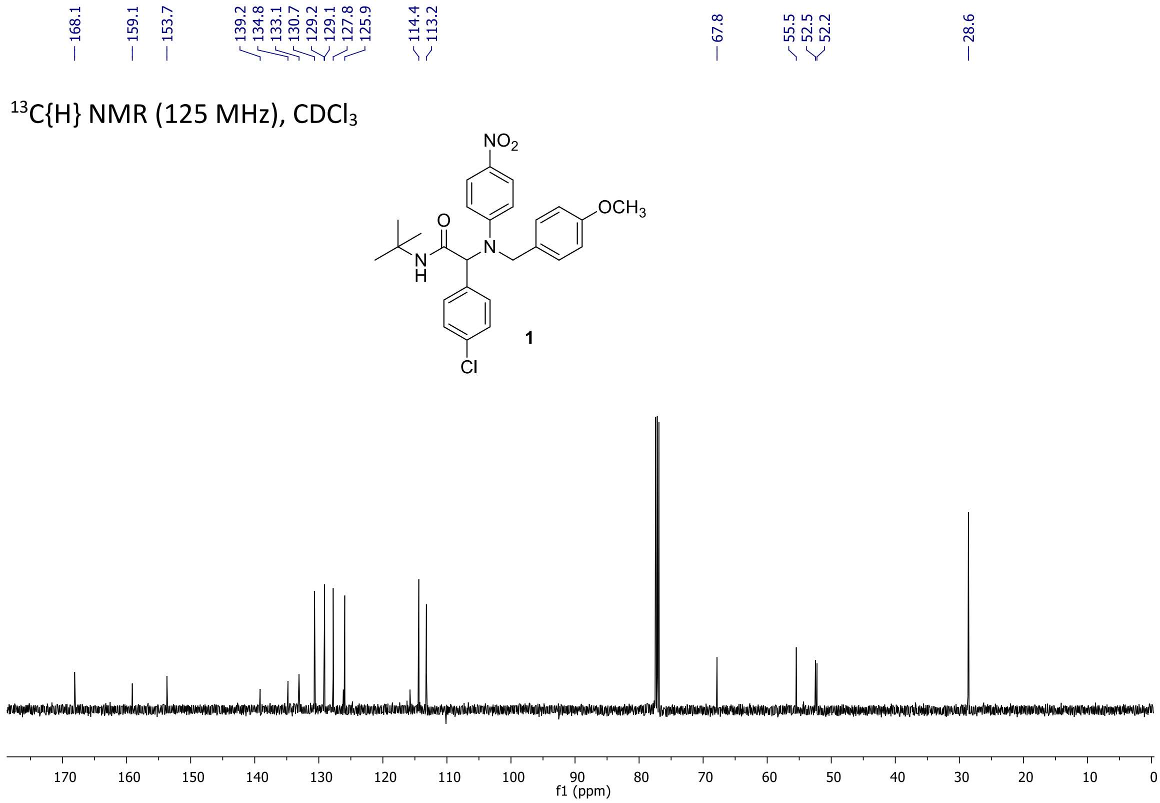

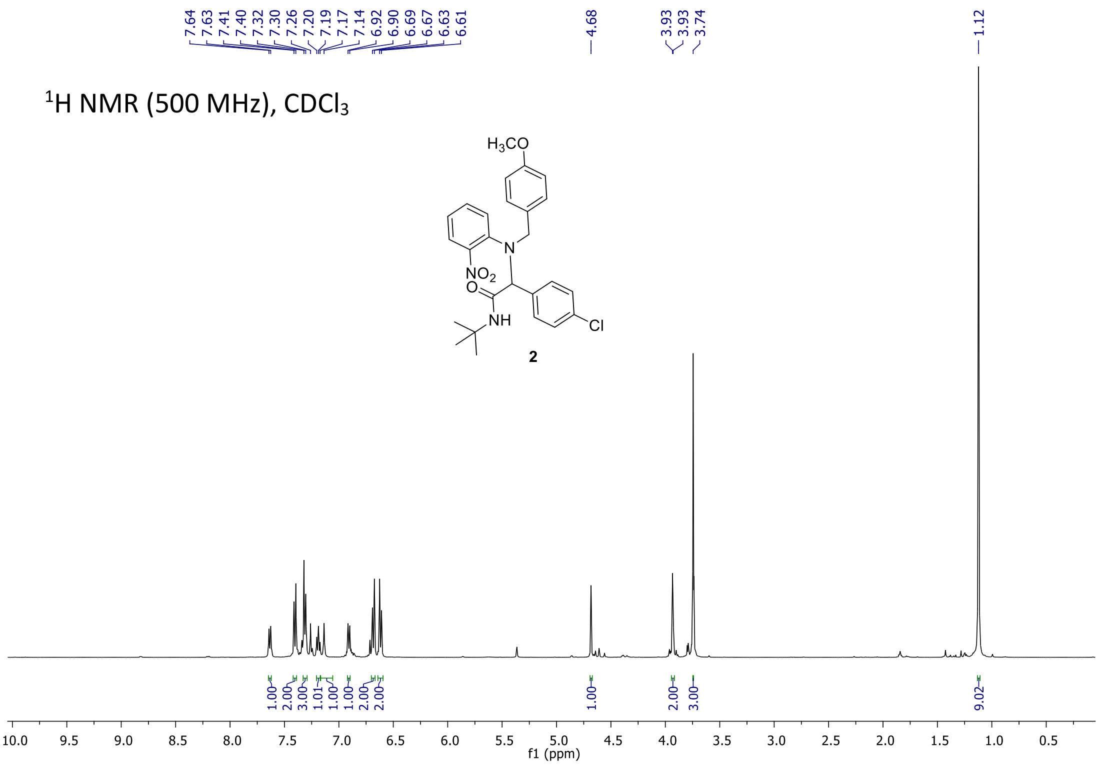
7.64
7.63
7.41
7.40
7.32
7.30
7.26
7.20
7.19
7.17
7.14
6.92
6.90
6.69
6.67
6.63
6.61
4.68
3.93
3.93
3.74
1.12
^{1}H NMR (500 MHz), $CDCl_3$
H_3CO
N
NO_2
O
NH
Cl
2
1.00
2.00
3.00
1.01
1.00
1.00
2.00
2.00
1.00
2.00
3.00
9.02
10.0 9.5 9.0 8.5 8.0 7.5 7.0 6.5 6.0 5.5 5.0 4.5 4.0 3.5 3.0 2.5 2.0 1.5 1.0 0.5
f1 (ppm)

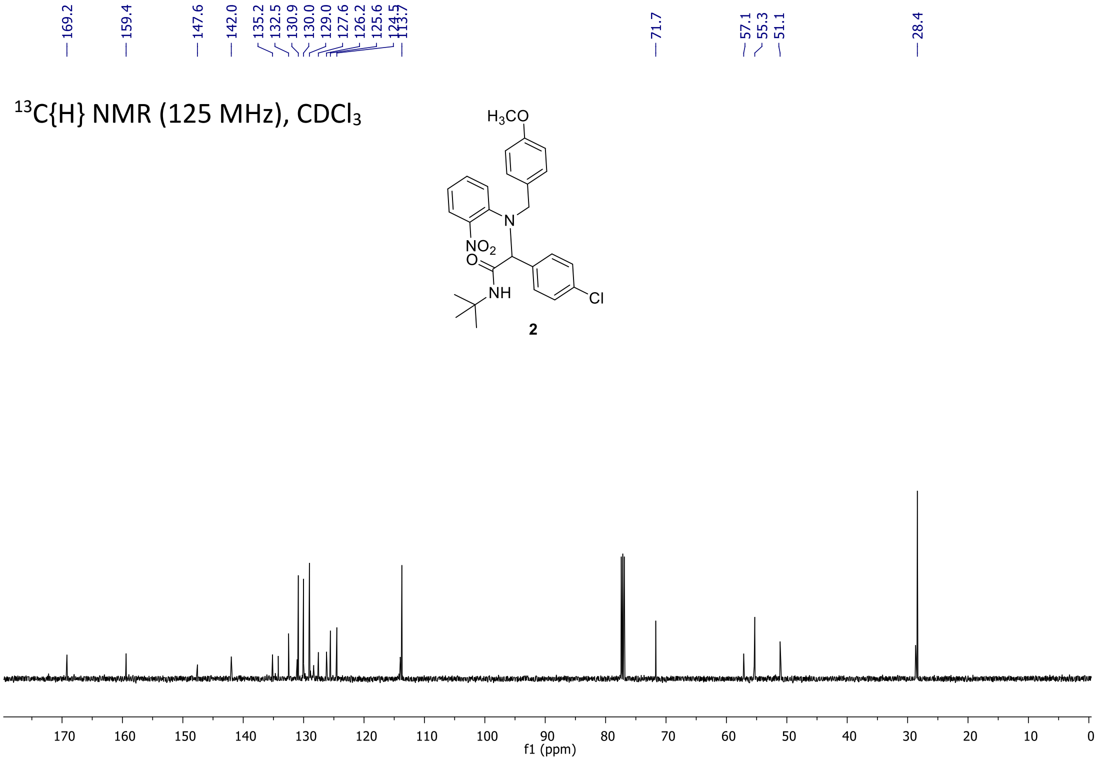
169.2
159.4
147.6
142.0
135.2
132.5
130.9
130.0
129.0
127.6
126.2
125.6
124.5
113.7
71.7
57.1
55.3
51.1
28.4
^{13}C{H} NMR (125 MHz), $CDCl_3$
H_3CO
N
NO_2
O
NH
Cl
2
170 160 150 140 130 120 110 100 90 80 70 60 50 40 30 20 10 0
f1 (ppm)

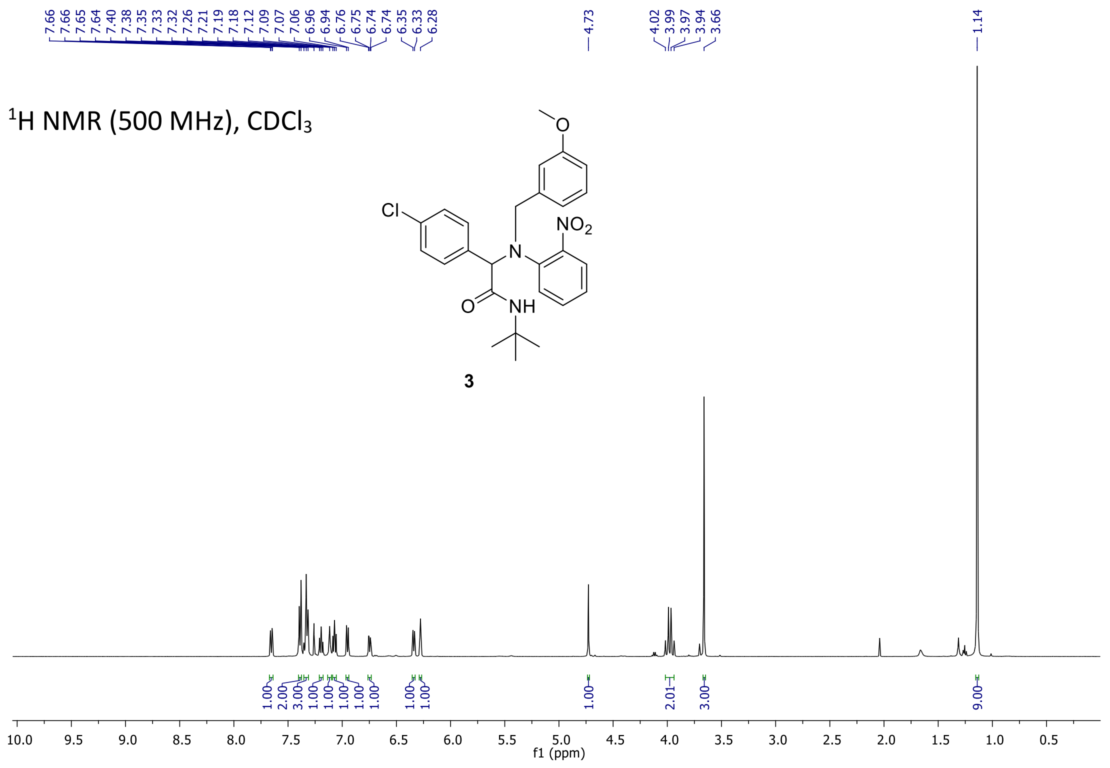
^{1}H NMR (500 MHz), CDCl$_3$
3
f1 (ppm)

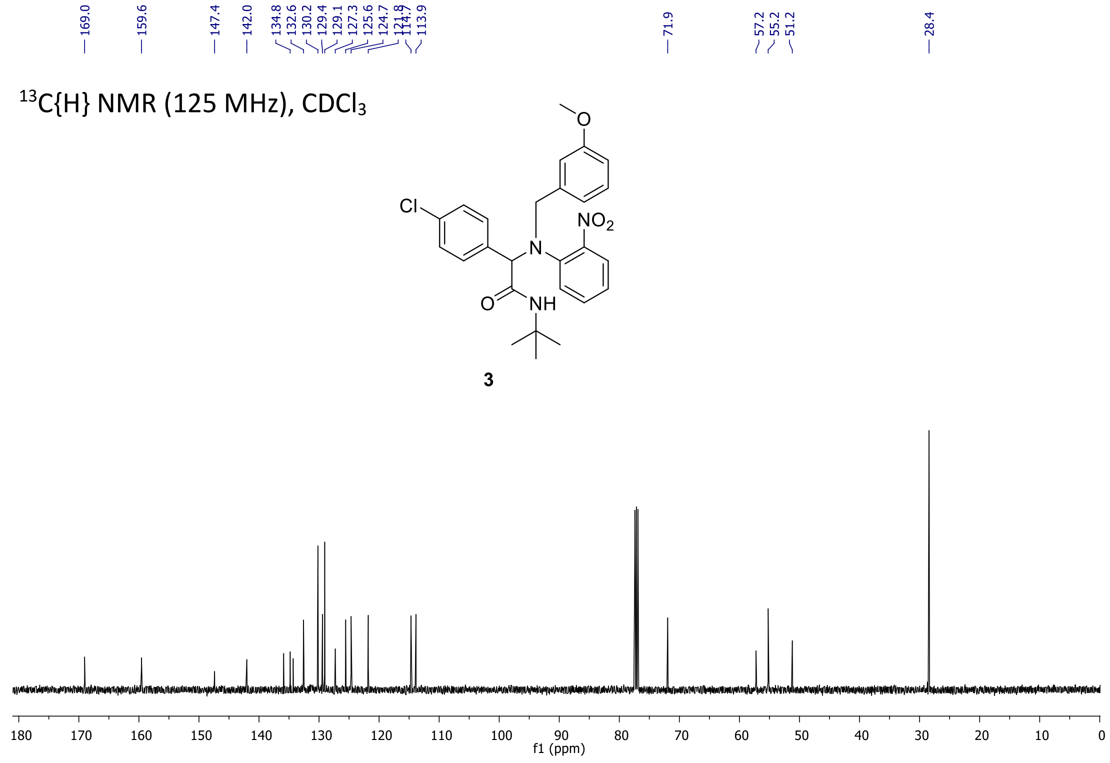
^{13}C{H} NMR (125 MHz), CDCl$_3$
3
f1 (ppm)

^1H NMR (500 MHz), $CDCl_3$

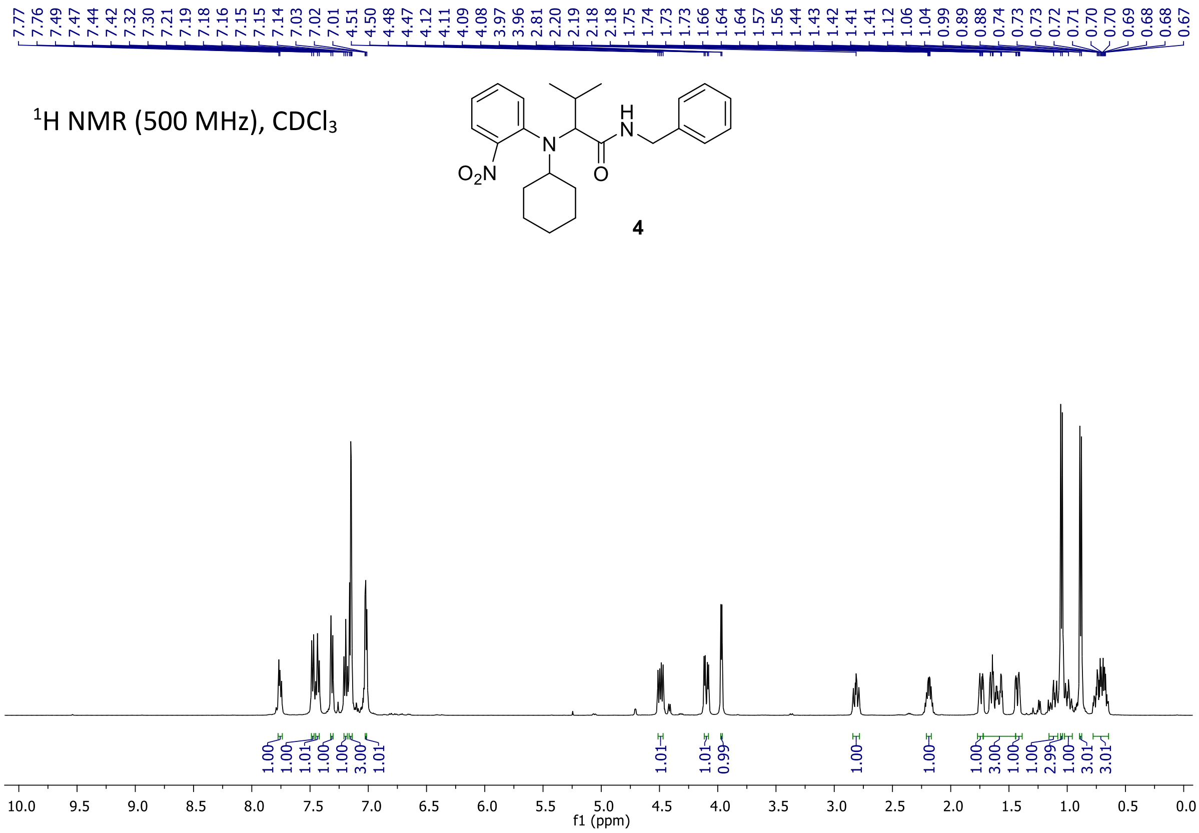

$^{13}C\{H\}$ NMR (125 MHz), $CDCl_3$

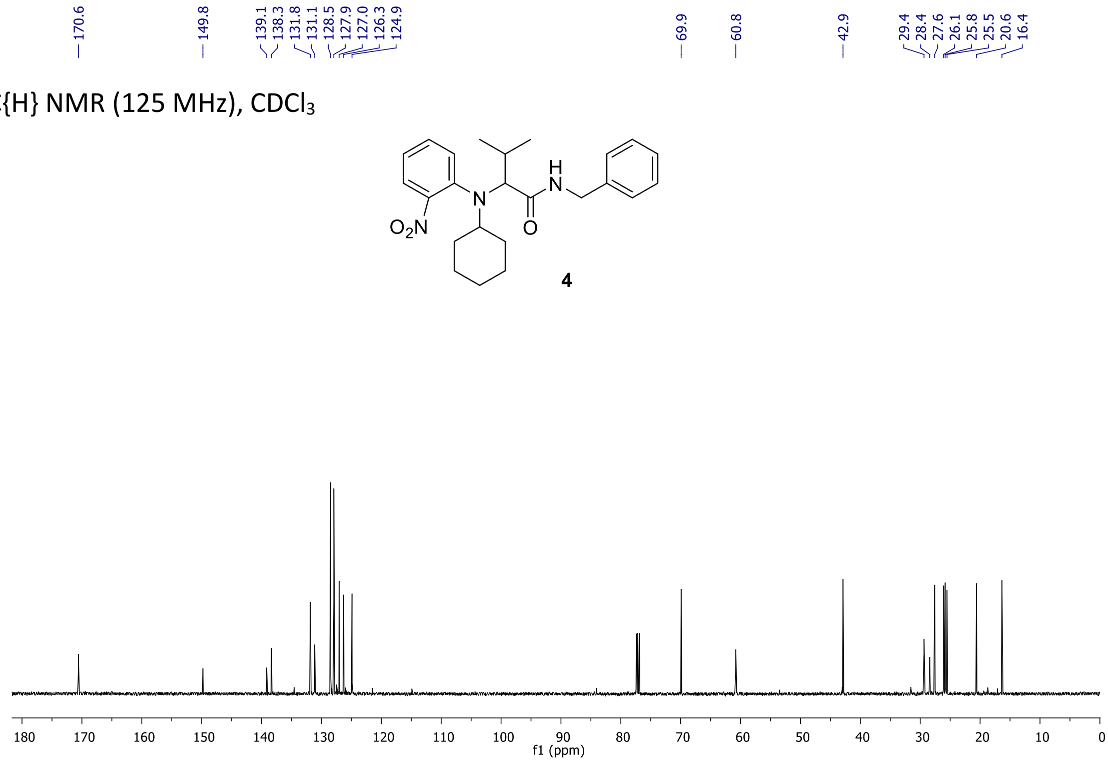

^{1}H NMR (500 MHz), CDCl$_3$

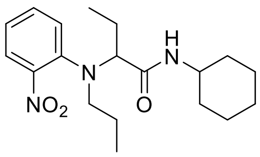

5

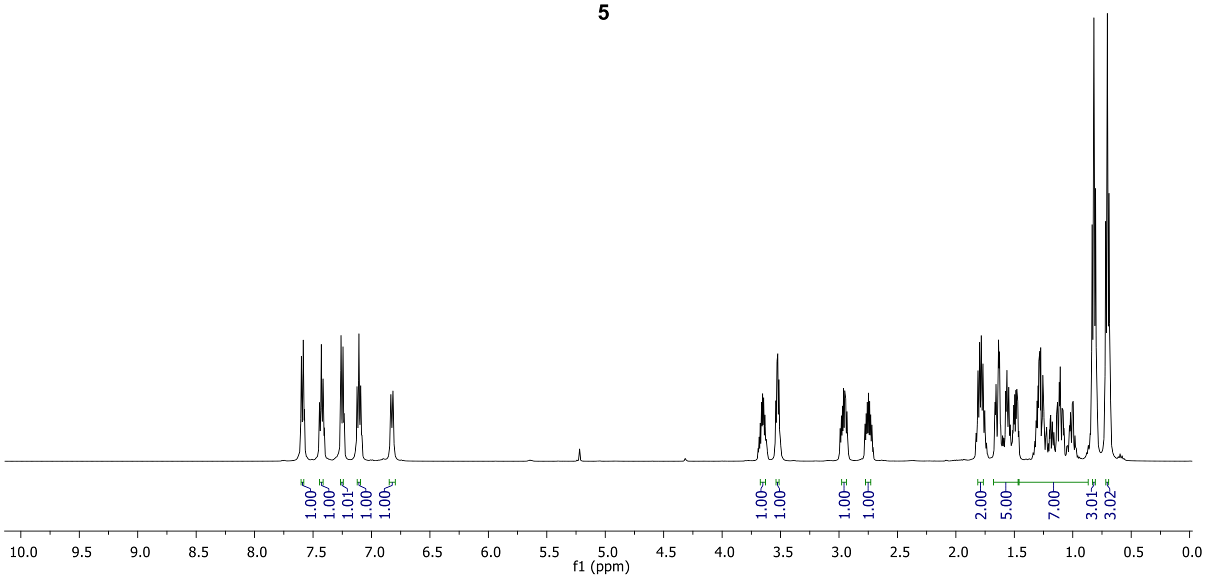

^{13}C{H} NMR (125 MHz), CDCl$_3$

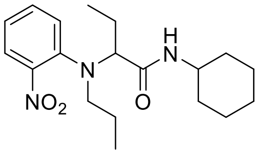

5

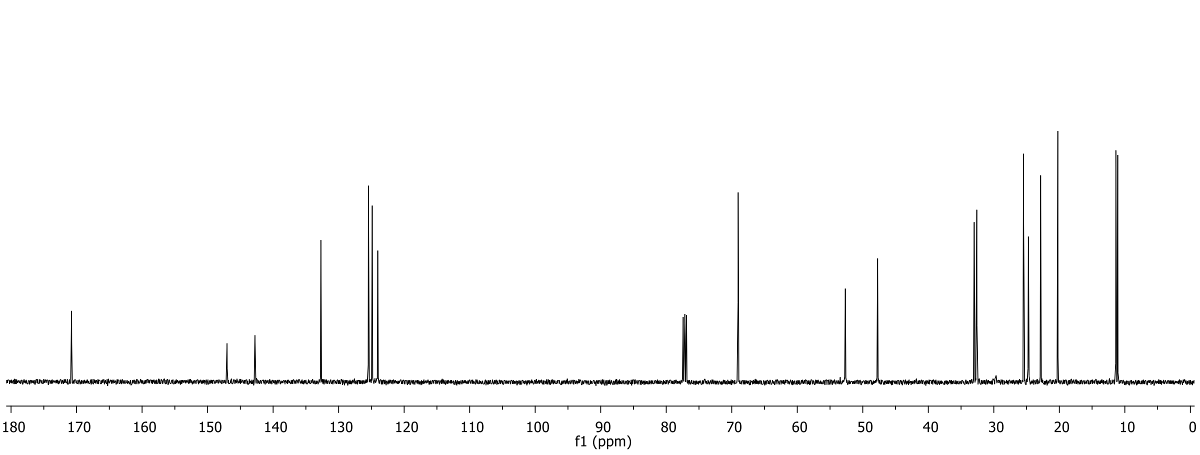

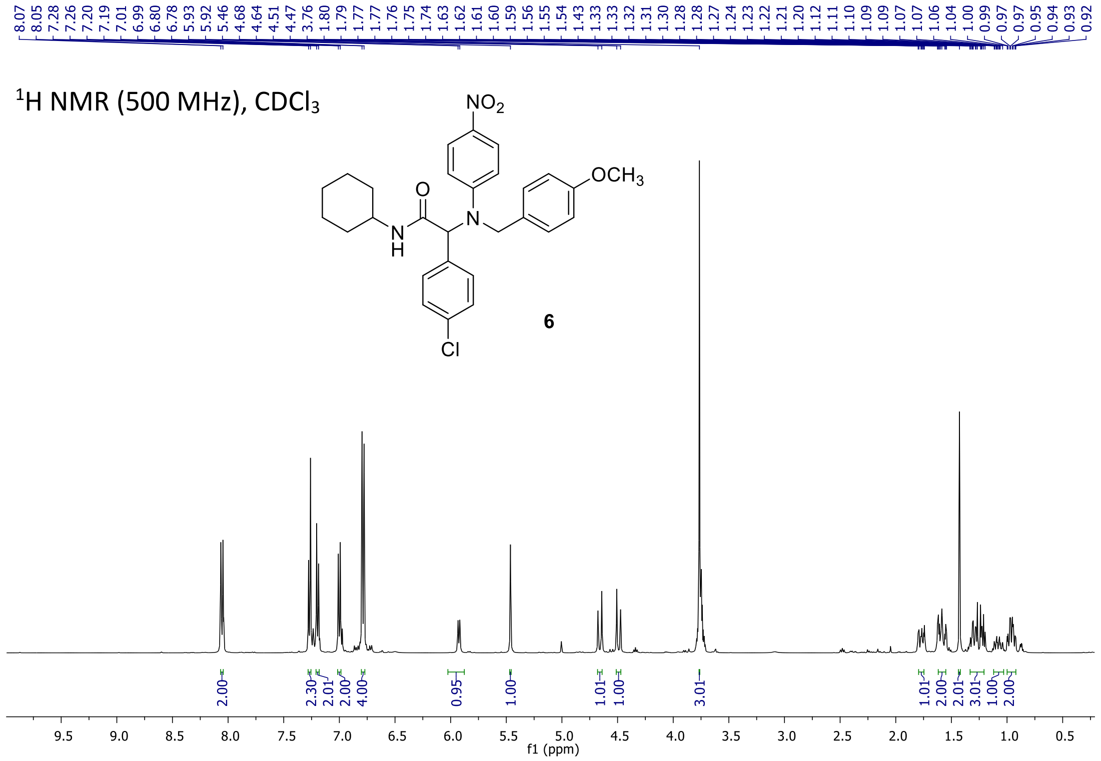
8.07 8.05 7.28 7.26 7.20 7.19 7.01 6.99 6.80 6.78 5.93 5.92 5.46 4.68 4.64 4.51 4.47 3.76 1.80 1.79 1.77 1.77 1.76 1.75 1.74 1.63 1.62 1.61 1.60 1.59 1.56 1.55 1.54 1.43 1.33 1.33 1.32 1.31 1.30 1.28 1.28 1.27 1.24 1.23 1.22 1.21 1.20 1.12 1.11 1.10 1.09 1.09 1.07 1.07 1.06 1.04 1.00 0.99 0.97 0.97 0.95 0.94 0.93 0.92
^{1}H NMR (500 MHz), $CDCl_3$
NO_2
OCH_3
O
N
N
H
Cl
6
2.00 2.30 2.01 2.00 4.00 0.95 1.00 1.01 1.00 3.01 1.01 2.00 2.01 3.01 1.00 2.00
f1 (ppm)

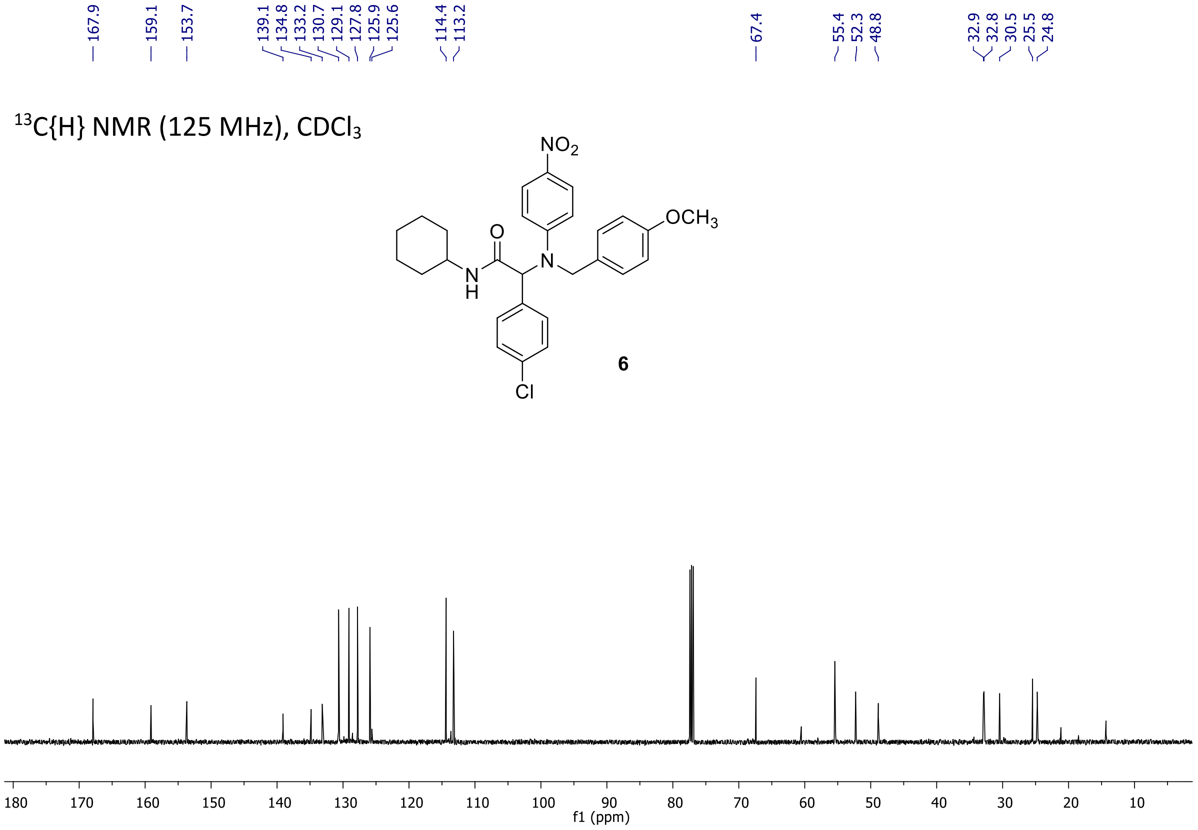
167.9 159.1 153.7 139.1 134.8 133.2 130.7 129.1 127.8 125.9 125.6 114.4 113.2 67.4 55.4 52.3 48.8 32.9 32.8 30.5 25.5 24.8
^{13}C{H} NMR (125 MHz), $CDCl_3$
NO_2
OCH_3
O
N
N
H
Cl
6
f1 (ppm)

^1H NMR (500 MHz), $CDCl_3$

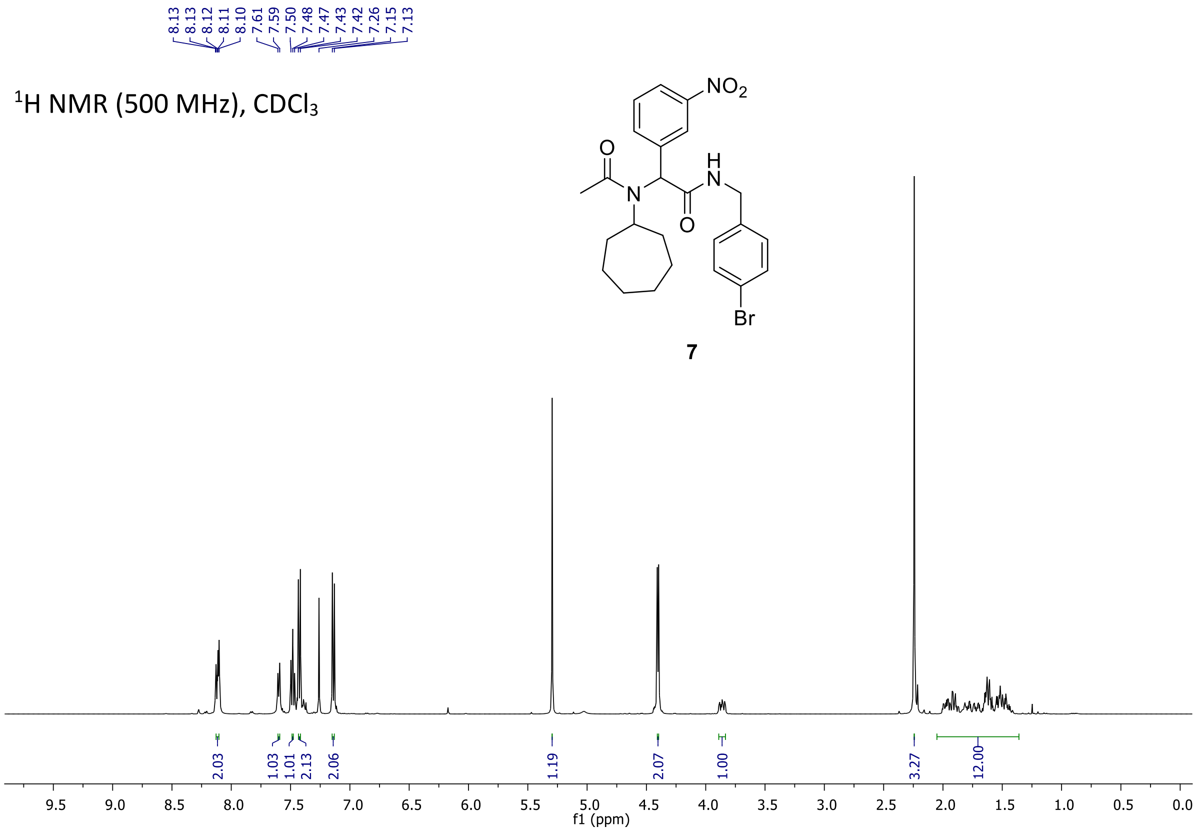

$^{13}C\{H\}$ NMR (125 MHz), $CDCl_3$

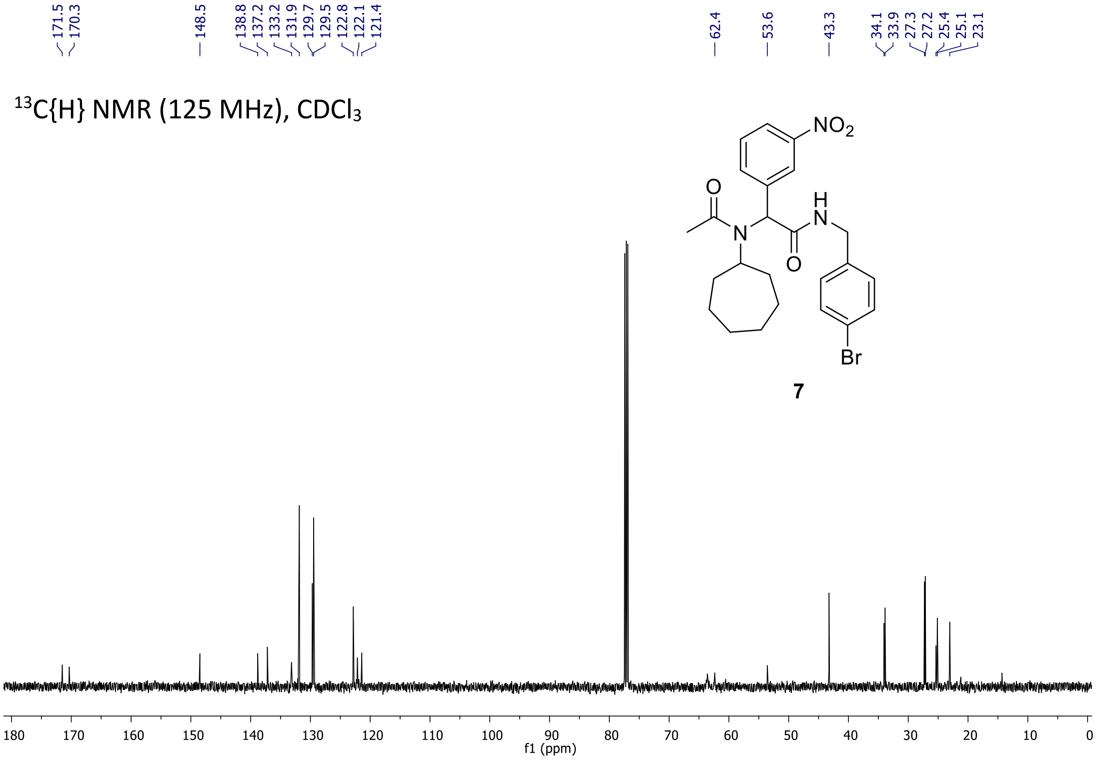

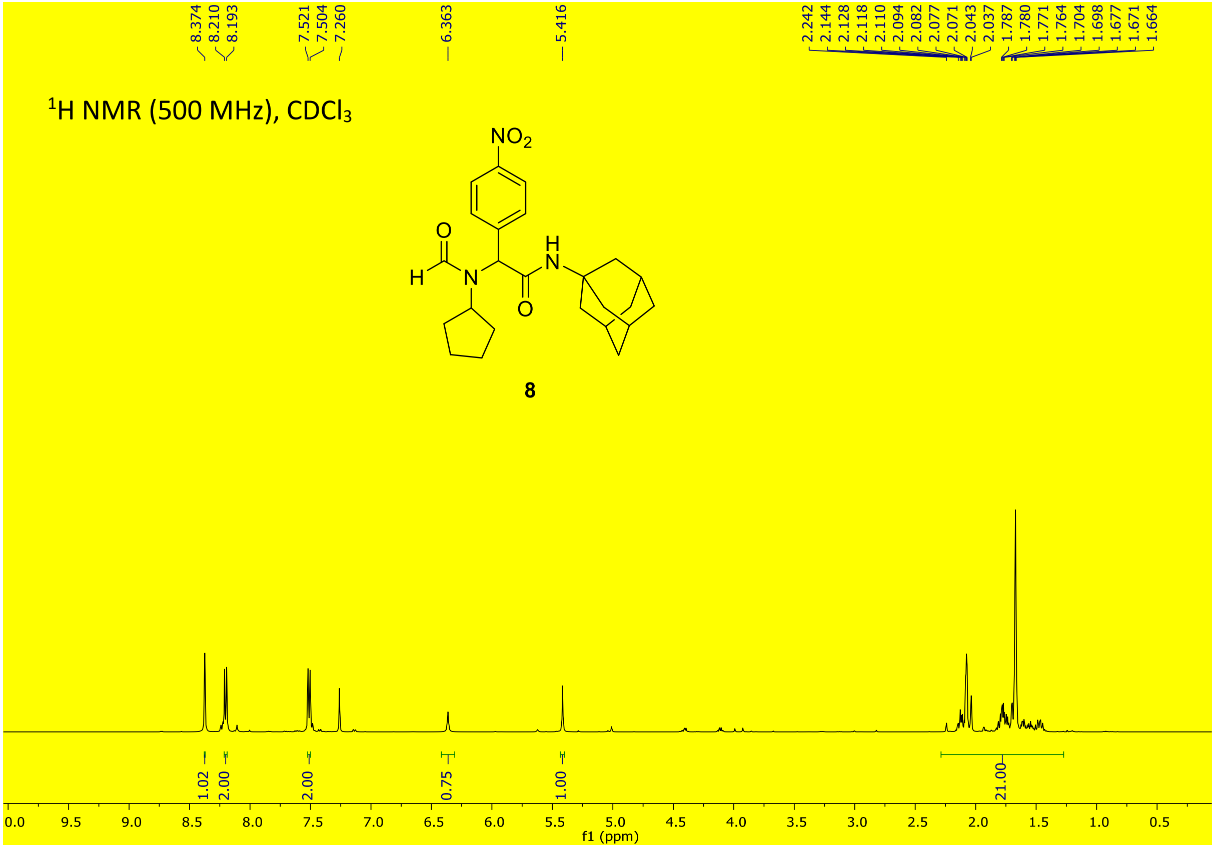
8.374
8.210
8.193
7.521
7.504
7.260
6.363
5.416
2.242
2.144
2.128
2.118
2.110
2.094
2.082
2.077
2.071
2.043
2.037
1.787
1.780
1.771
1.764
1.704
1.698
1.677
1.671
1.664
^{1}H NMR (500 MHz), CDCl$_3$
NO$_2$
O
H
N
H
N
O
8
1.02
2.00
2.00
0.75
1.00
21.00
f1 (ppm)

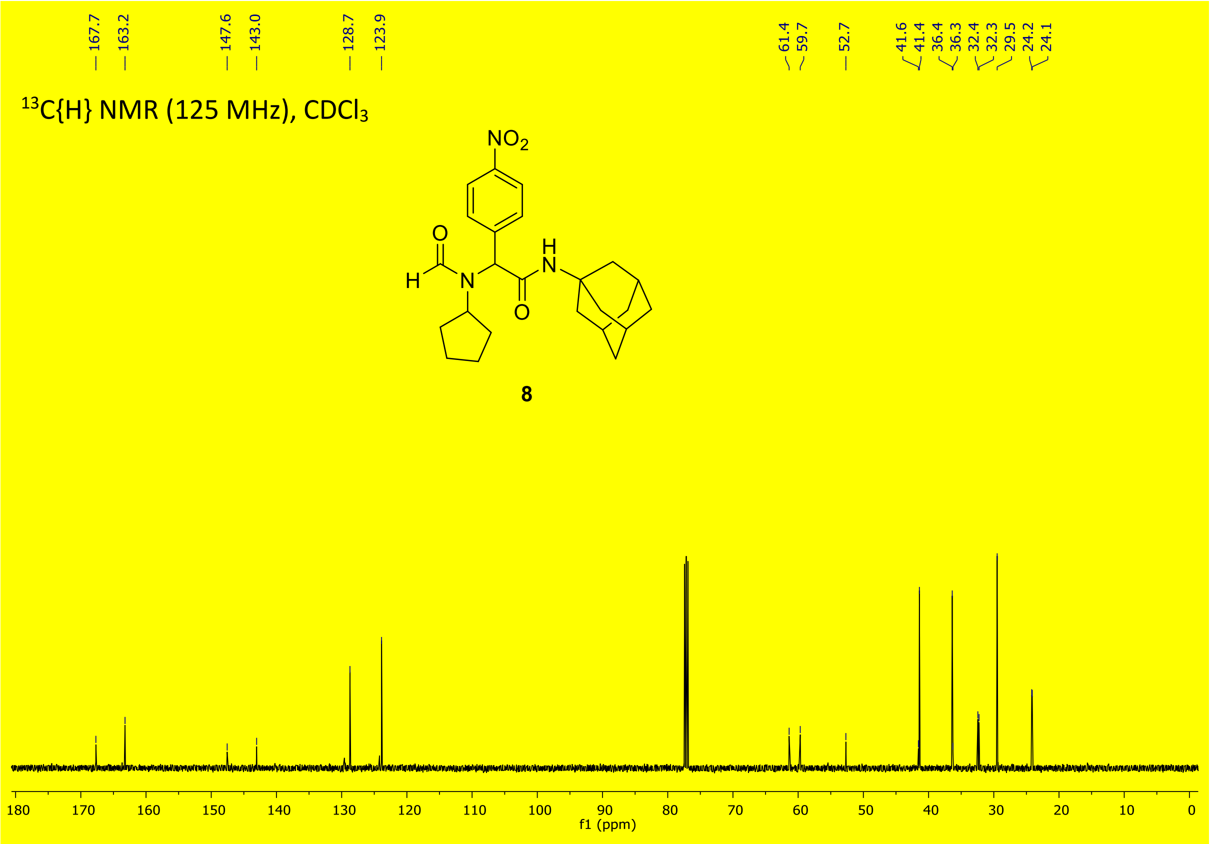
167.7
163.2
147.6
143.0
128.7
123.9
61.4
59.7
52.7
41.6
41.4
36.4
36.3
32.4
32.3
29.5
24.2
24.1
^{13}C{H} NMR (125 MHz), CDCl$_3$
NO$_2$
O
H
N
H
N
O
8
f1 (ppm)

^{1}H NMR (500 MHz), $CDCl_3$

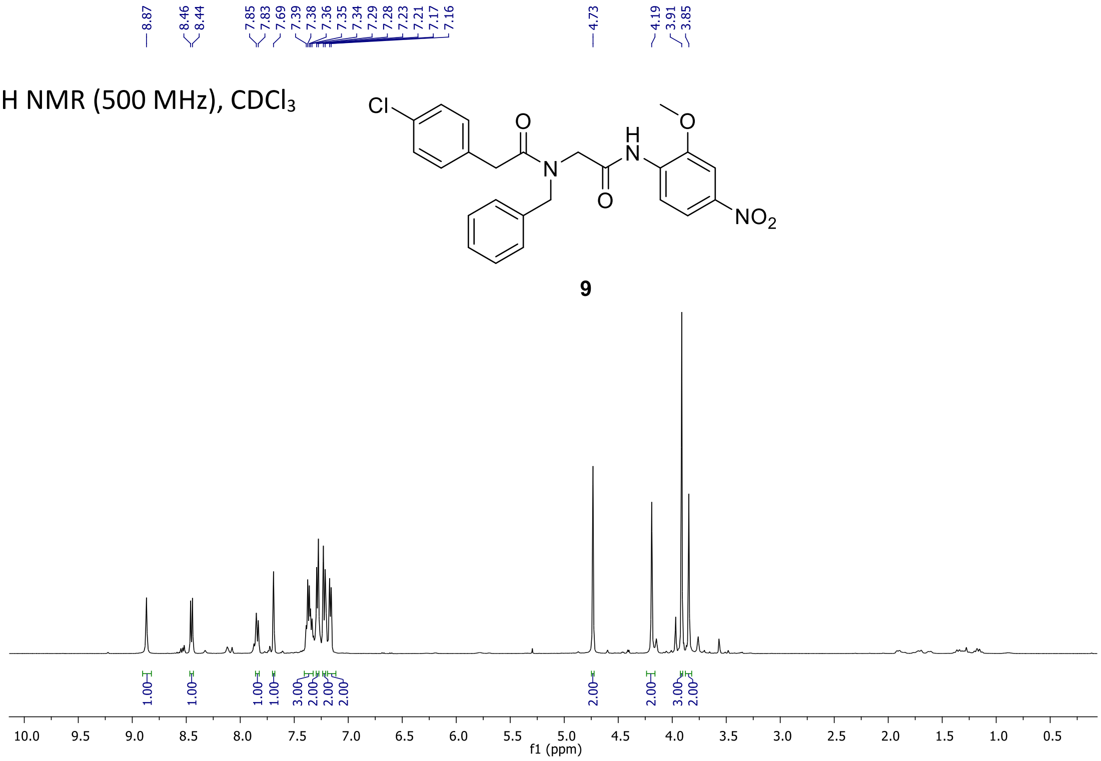

9

^{13}C{H} NMR (125 MHz), $CDCl_3$

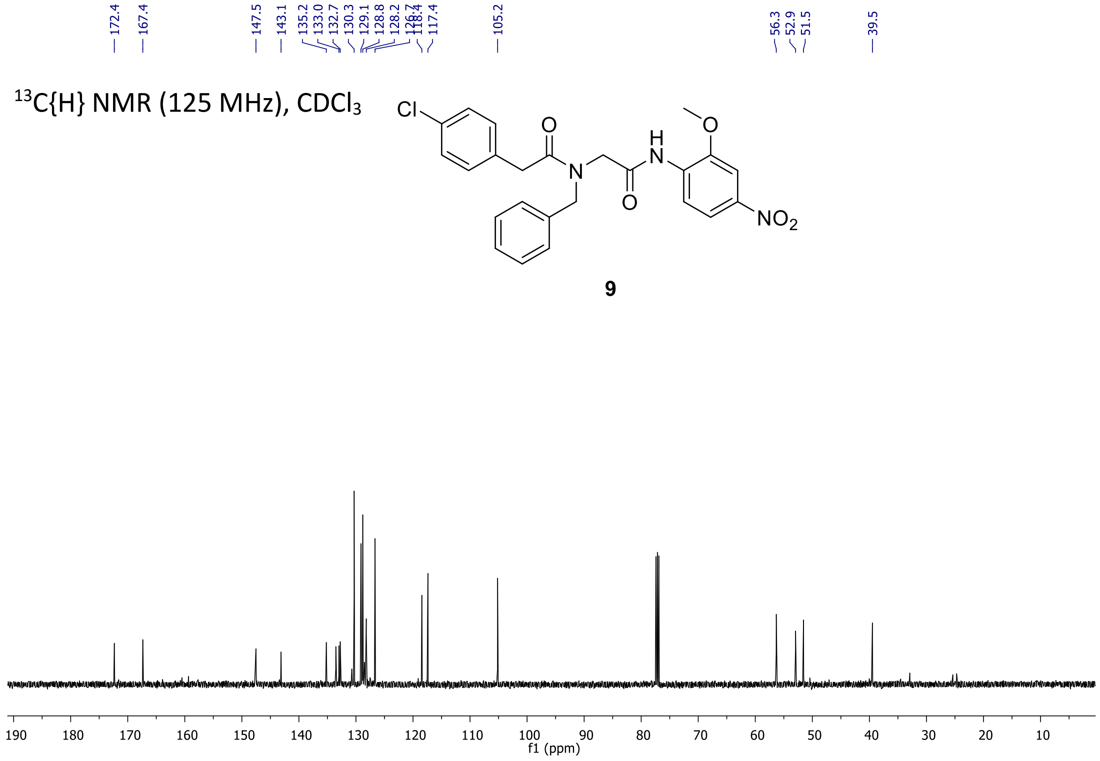

9

^{1}H NMR (500 MHz), $CDCl_3$

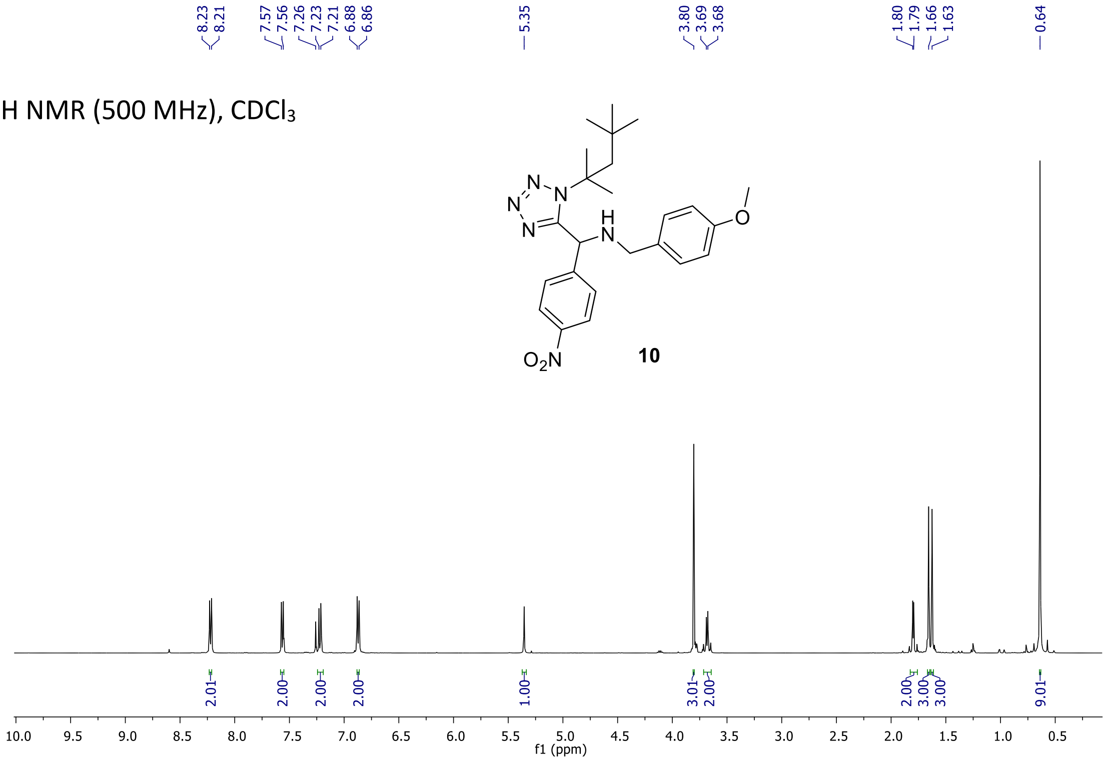

$^{13}C\{H\}$ NMR (125 MHz), $CDCl_3$

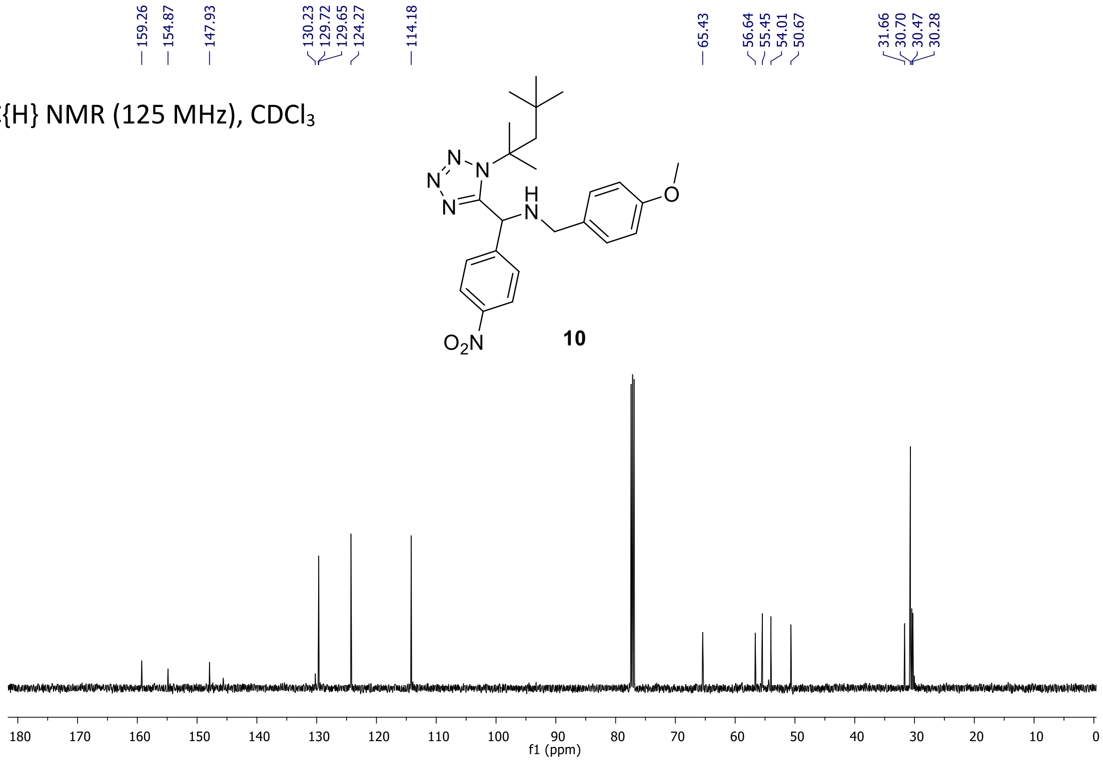

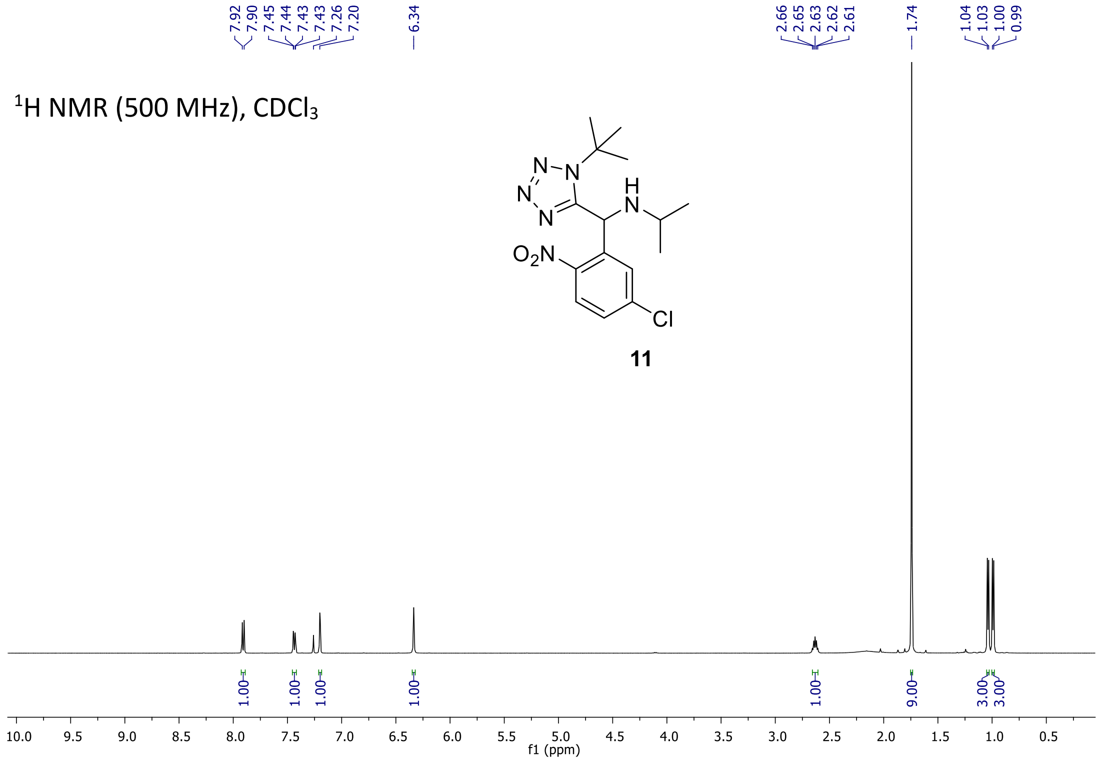
7.92
7.90
7.45
7.44
7.43
7.43
7.26
7.20
6.34
2.66
2.65
2.63
2.62
2.61
1.74
1.04
1.03
1.00
0.99
^{1}H NMR (500 MHz), CDCl$_3$
N
N
N
N
H
N
O_2N
Cl
11
1.00
1.00
1.00
1.00
1.00
9.00
3.00
3.00
10.0 9.5 9.0 8.5 8.0 7.5 7.0 6.5 6.0 5.5 5.0 4.5 4.0 3.5 3.0 2.5 2.0 1.5 1.0 0.5
f1 (ppm)

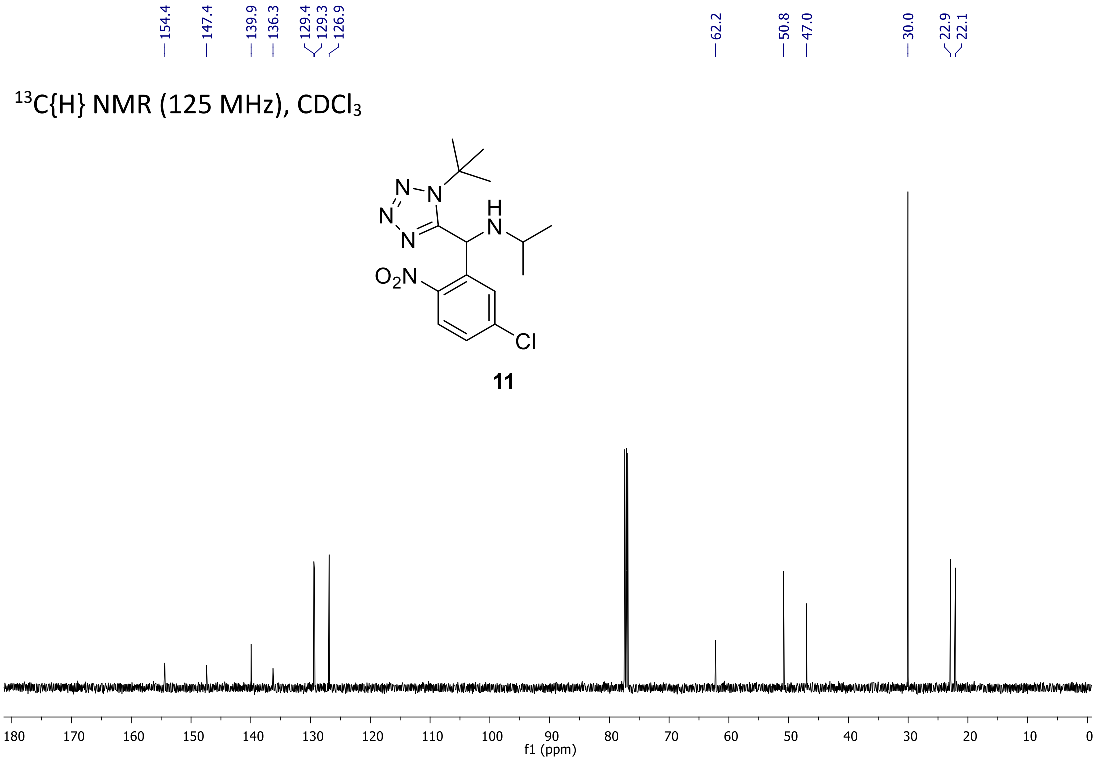
154.4
147.4
139.9
136.3
129.4
129.3
126.9
62.2
50.8
47.0
30.0
22.9
22.1
^{13}C{H} NMR (125 MHz), CDCl$_3$
N
N
N
N
H
N
O_2N
Cl
11
180 170 160 150 140 130 120 110 100 90 80 70 60 50 40 30 20 10 0
f1 (ppm)

^{1}H NMR (500 MHz), $CDCl_3$

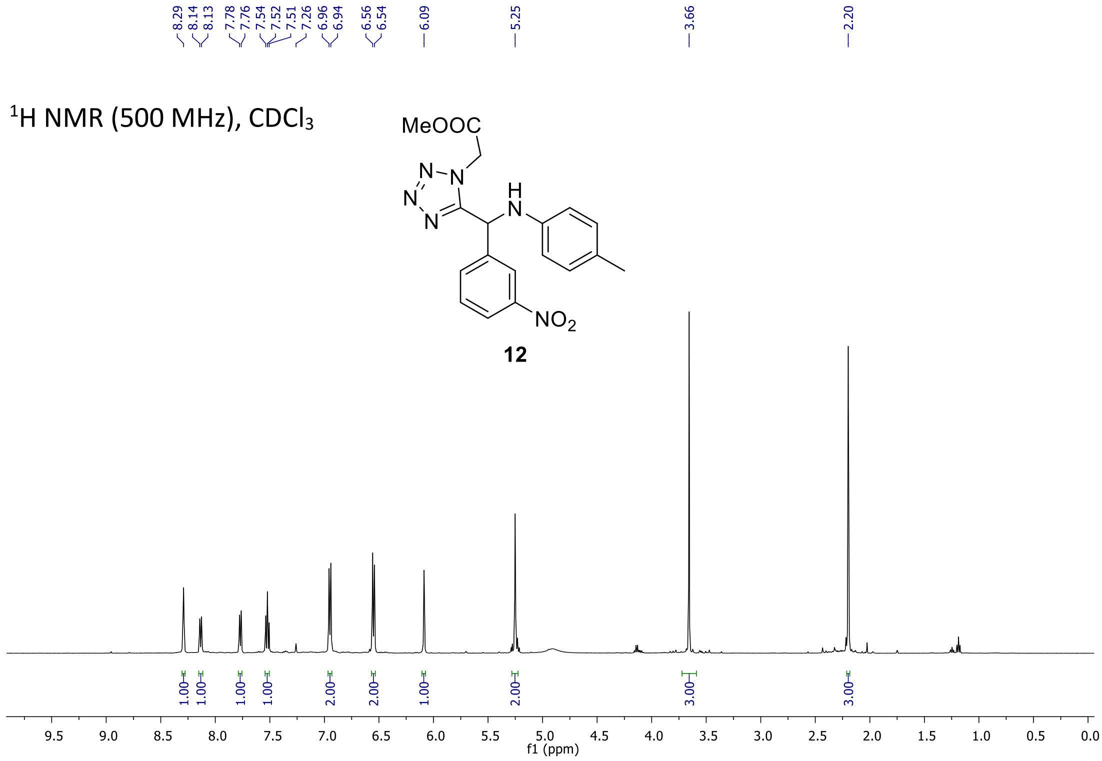

^{13}C{H} NMR (125 MHz), $CDCl_3$

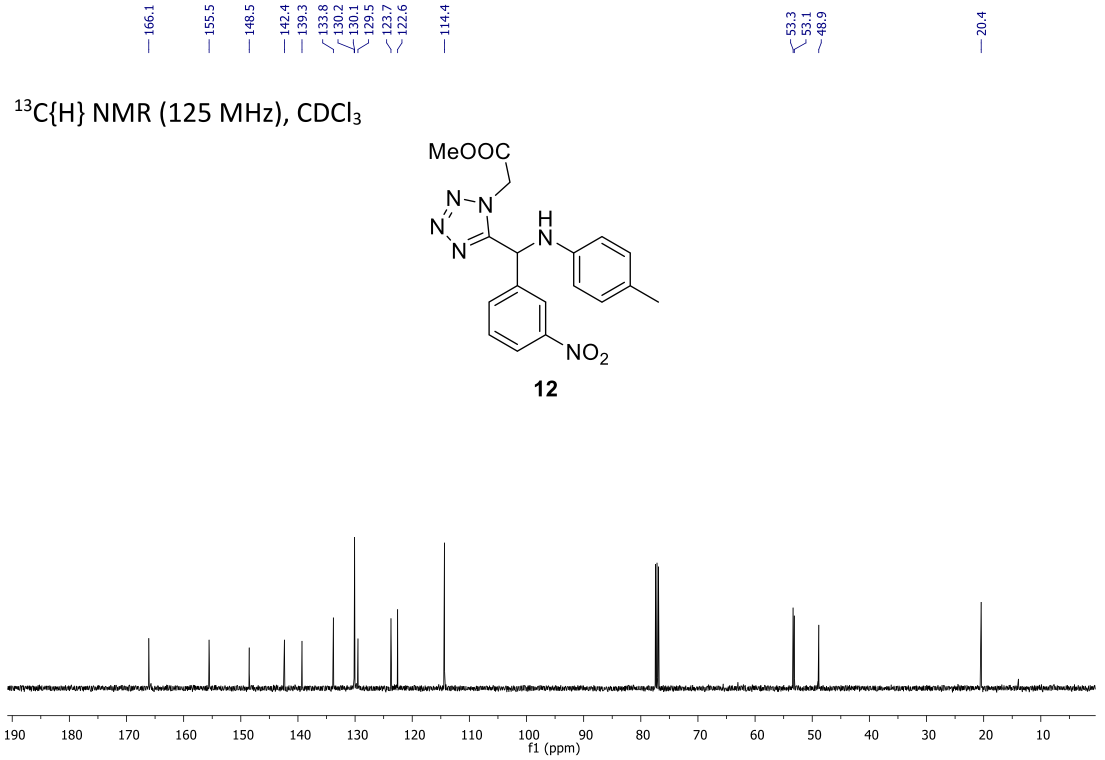

^{1}H NMR (500 MHz), $CDCl_3$

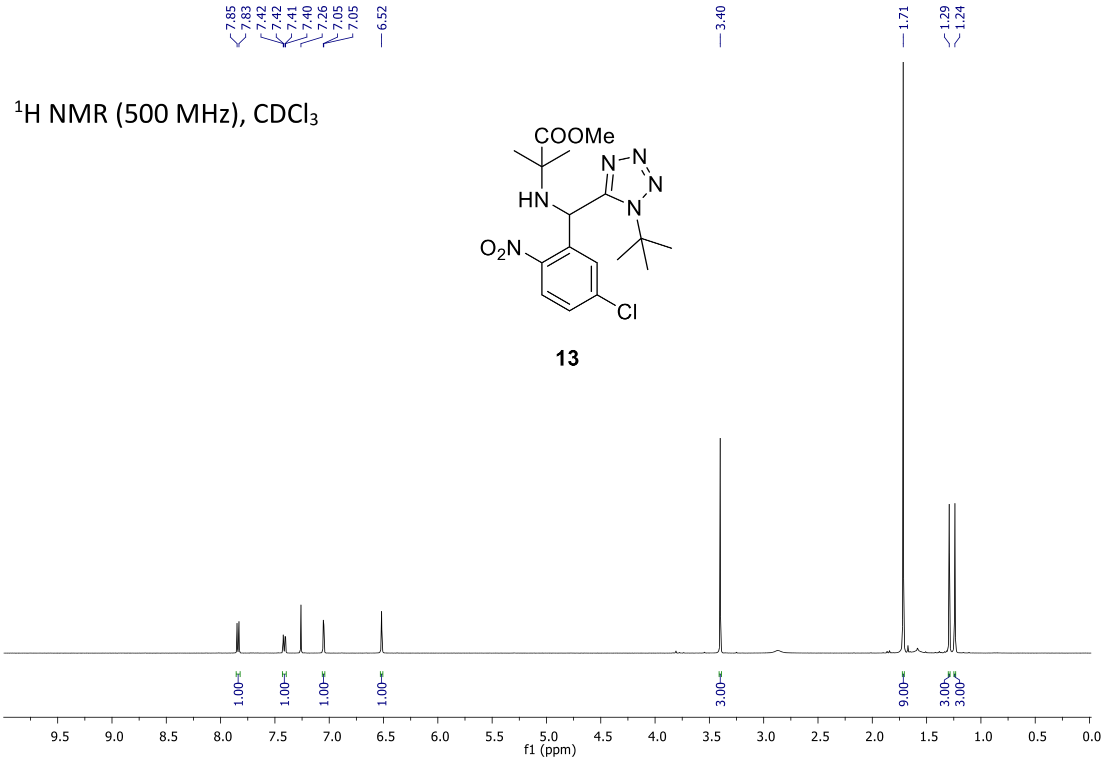

^{13}C{H} NMR (125 MHz), $CDCl_3$

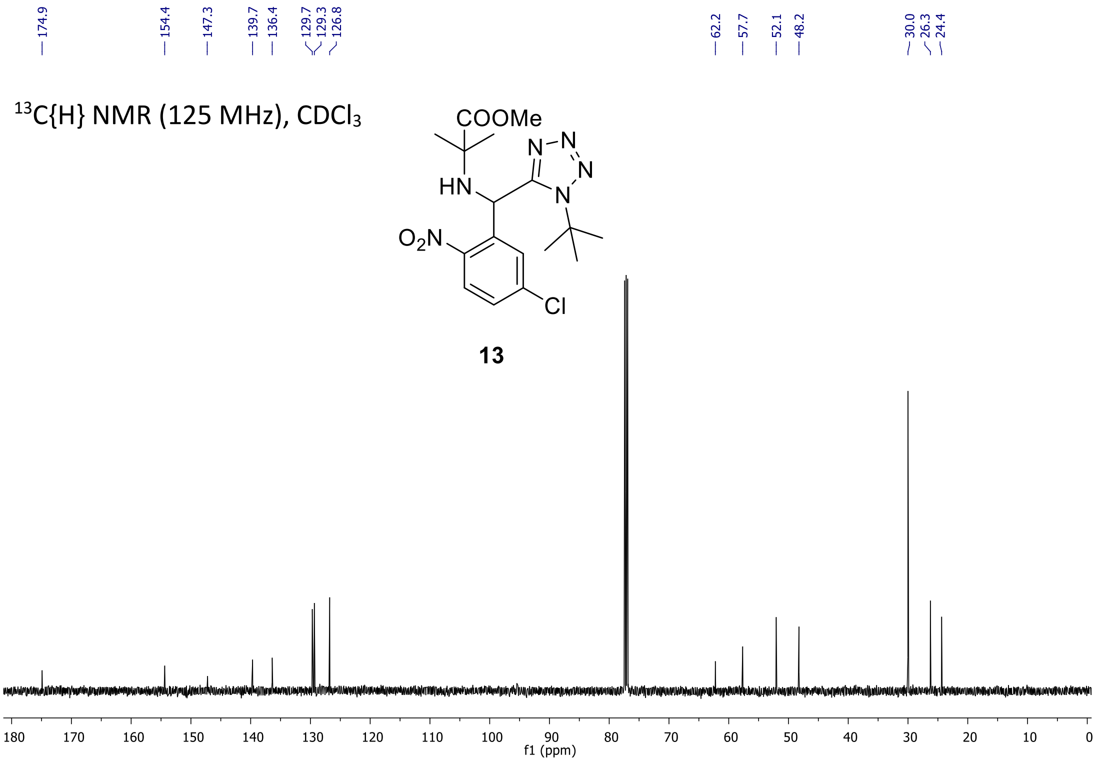

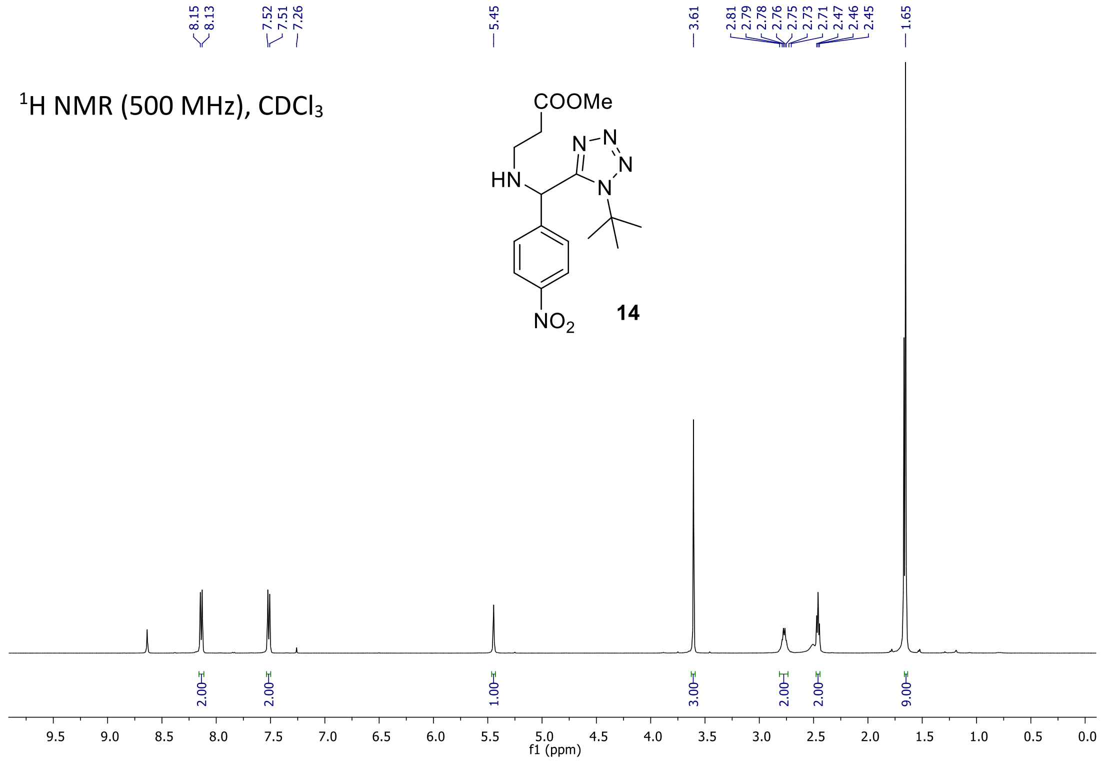

8.15
8.13
7.52
7.51
7.26
5.45
3.61
2.81
2.79
2.78
2.76
2.75
2.73
2.71
2.47
2.46
2.45
1.65
¹H NMR (500 MHz), CDCl₃
COOMe
HN
N
14
2.00
2.00
1.00
3.00
2.00
2.00
9.00
f1 (ppm)

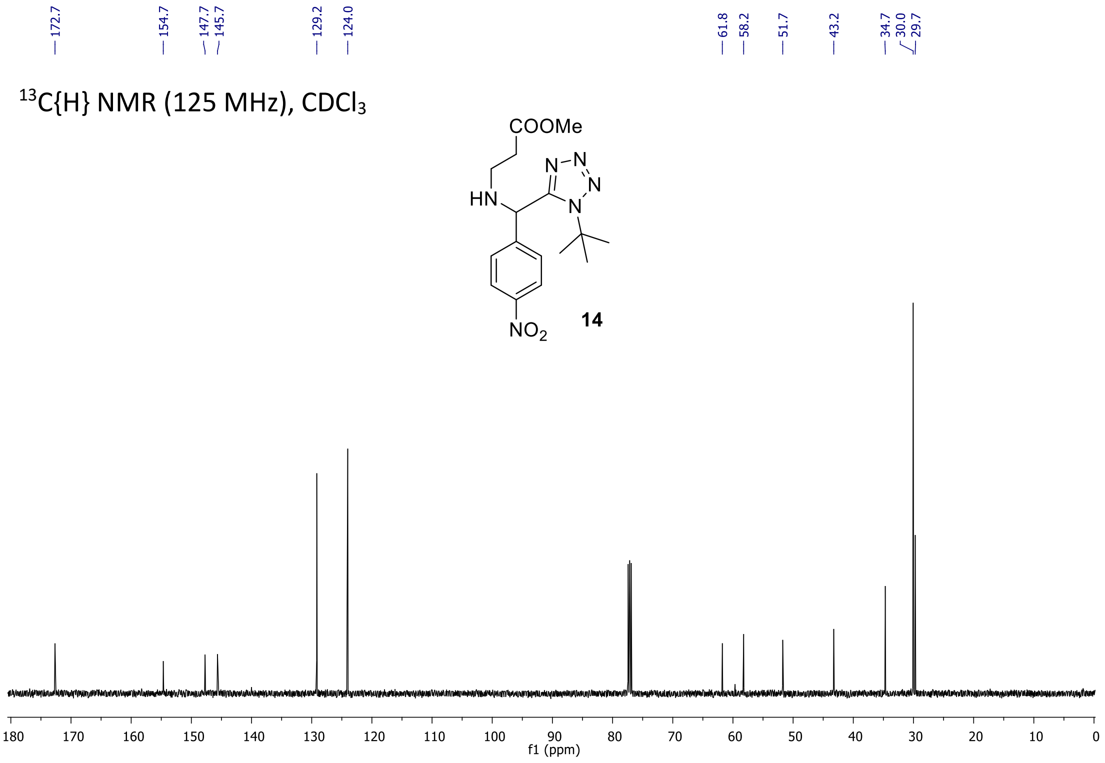

172.7
154.7
147.7
145.7
129.2
124.0
61.8
58.2
51.7
43.2
34.7
30.0
29.7
13C{H} NMR (125 MHz), CDCl₃
COOMe
HN
14
f1 (ppm)

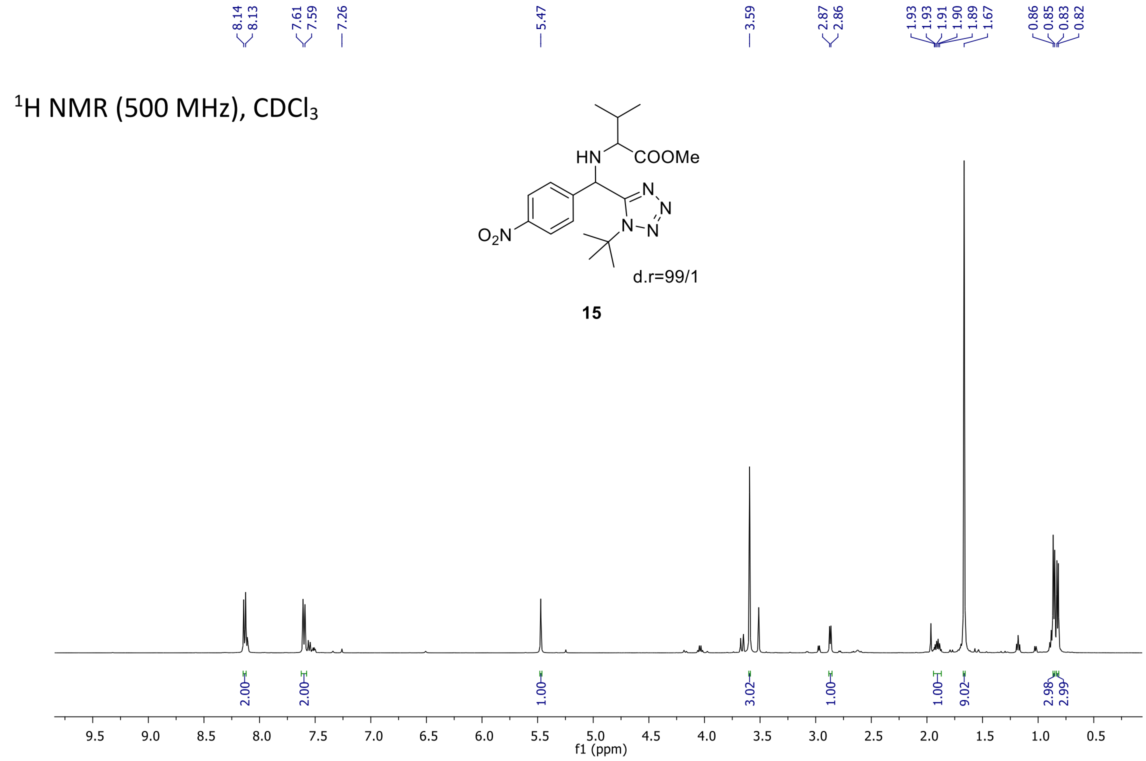

8.14
8.13
7.61
7.59
7.26
5.47
3.59
2.87
2.86
1.93
1.93
1.91
1.90
1.89
1.67
0.86
0.85
0.83
0.82
^{1}H NMR (500 MHz), CDCl$_3$
HN
COOMe
N
N
N
N
O$_2$N
d.r=99/1
15
2.00
2.00
1.00
3.02
1.00
1.00
9.02
2.98
2.99
9.5
9.0
8.5
8.0
7.5
7.0
6.5
6.0
5.5
5.0
4.5
4.0
3.5
3.0
2.5
2.0
1.5
1.0
0.5
f1 (ppm)

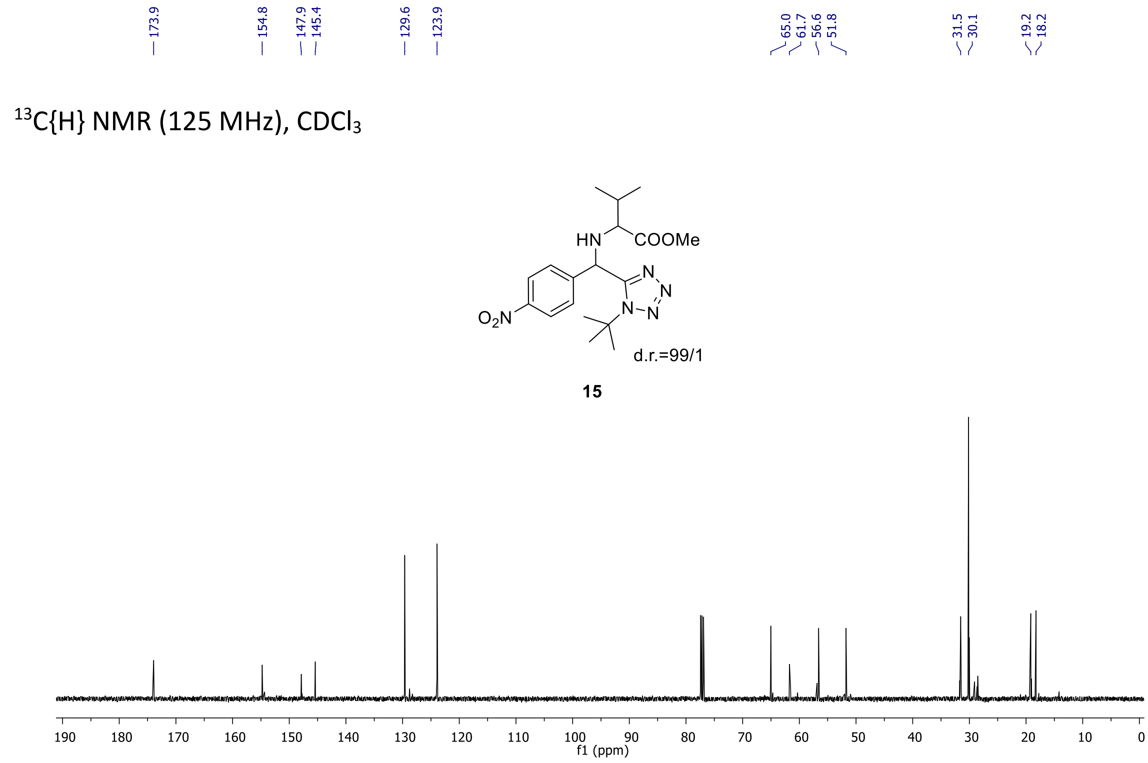

173.9
154.8
147.9
145.4
129.6
123.9
65.0
61.7
56.6
51.8
31.5
30.1
19.2
18.2
^{13}C{H} NMR (125 MHz), CDCl$_3$
HN
COOMe
N
N
N
N
O$_2$N
d.r.=99/1
15
190
180
170
160
150
140
130
120
110
100
90
80
70
60
50
40
30
20
10
0
f1 (ppm)

^1H NMR (500 MHz), $CDCl_3$

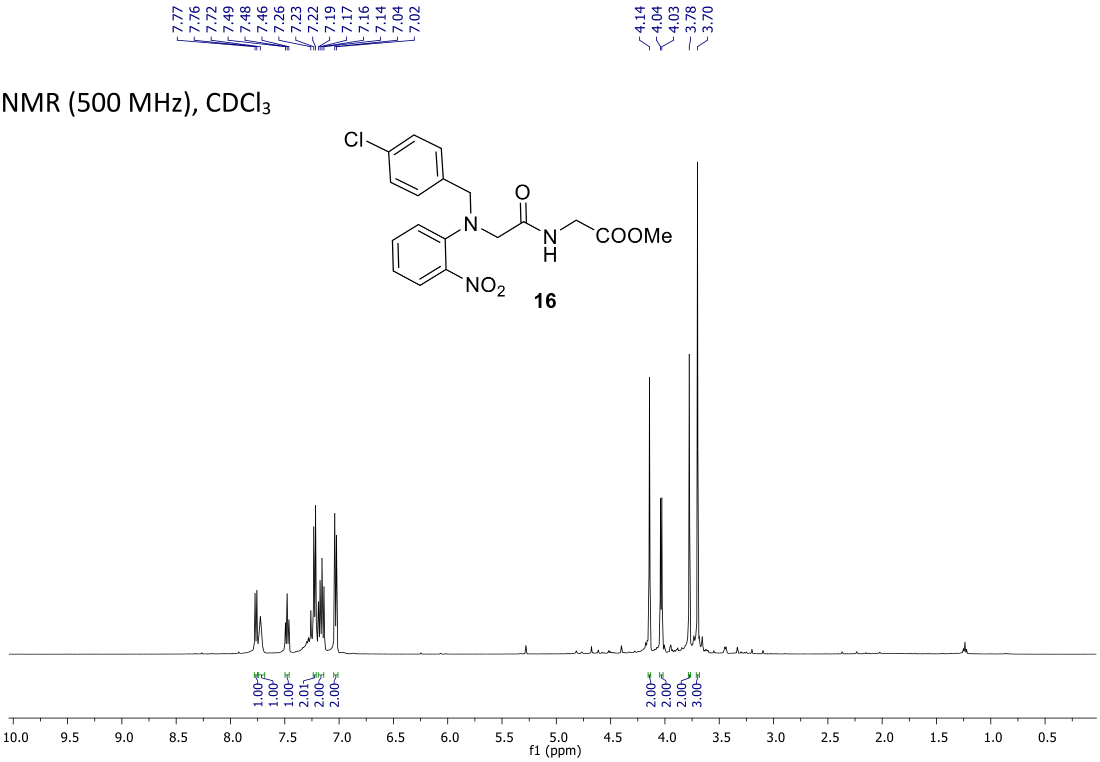

$^{13}C\{H\}$ NMR (125 MHz), $CDCl_3$

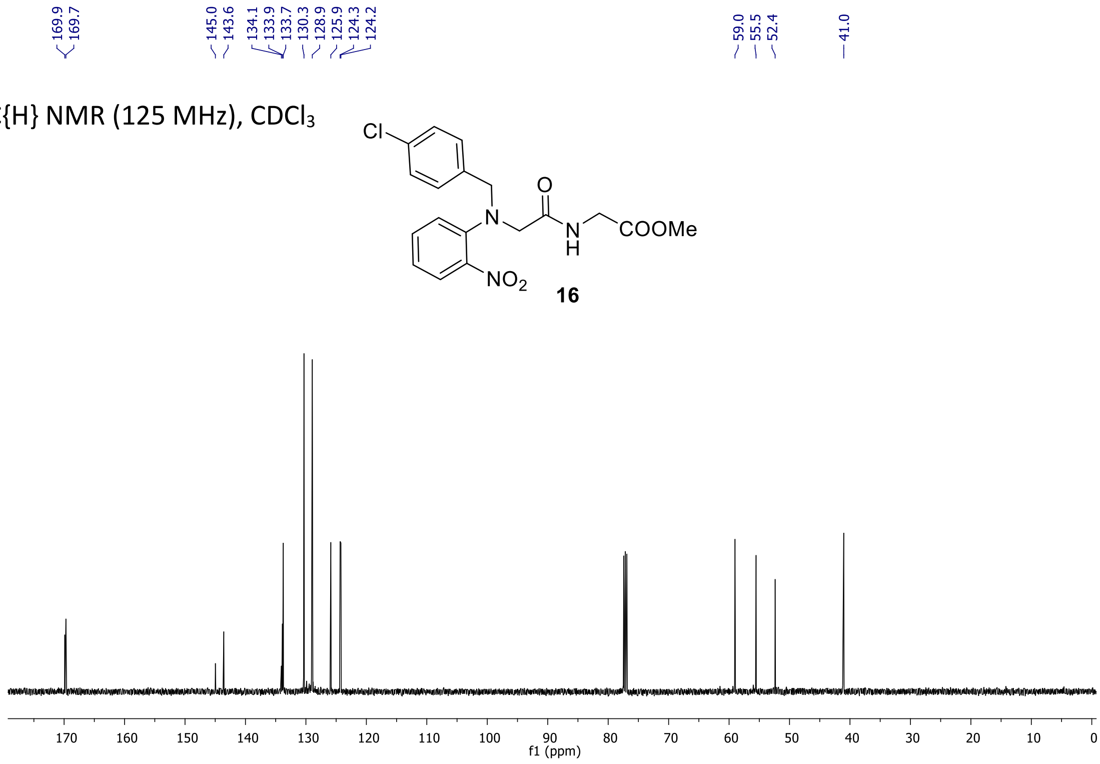

^{1}H NMR (500 MHz), $CDCl_3$

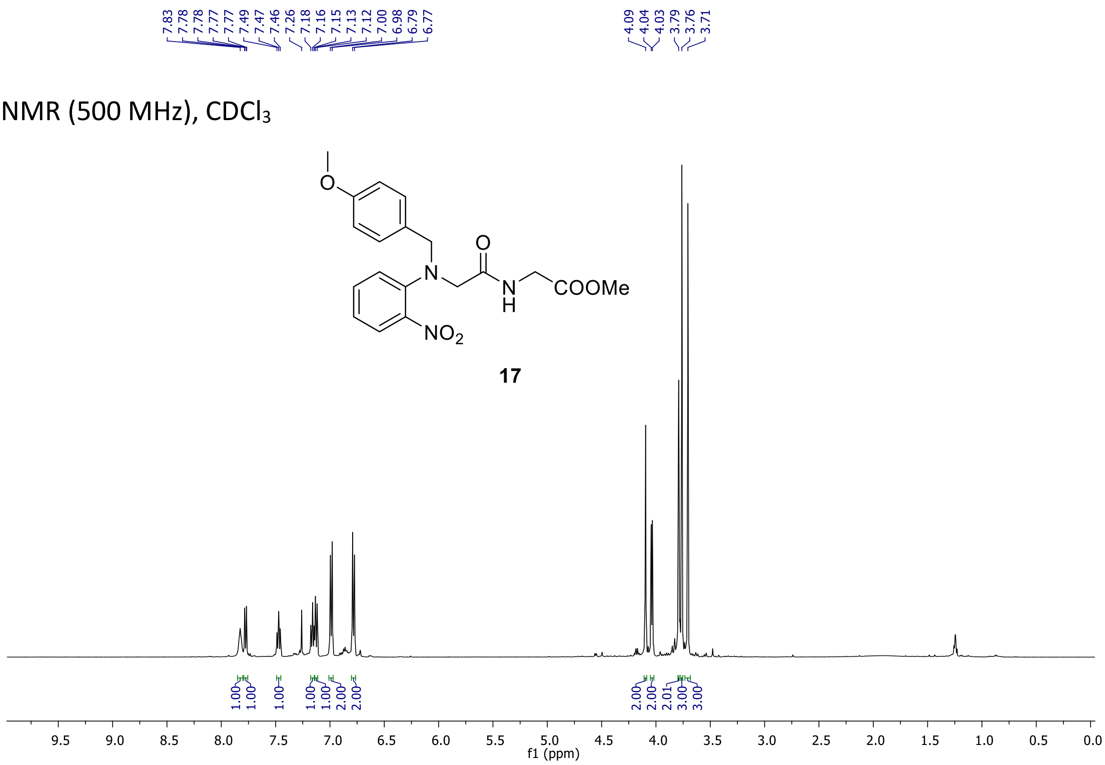

^{13}C{H} NMR (125 MHz), $CDCl_3$

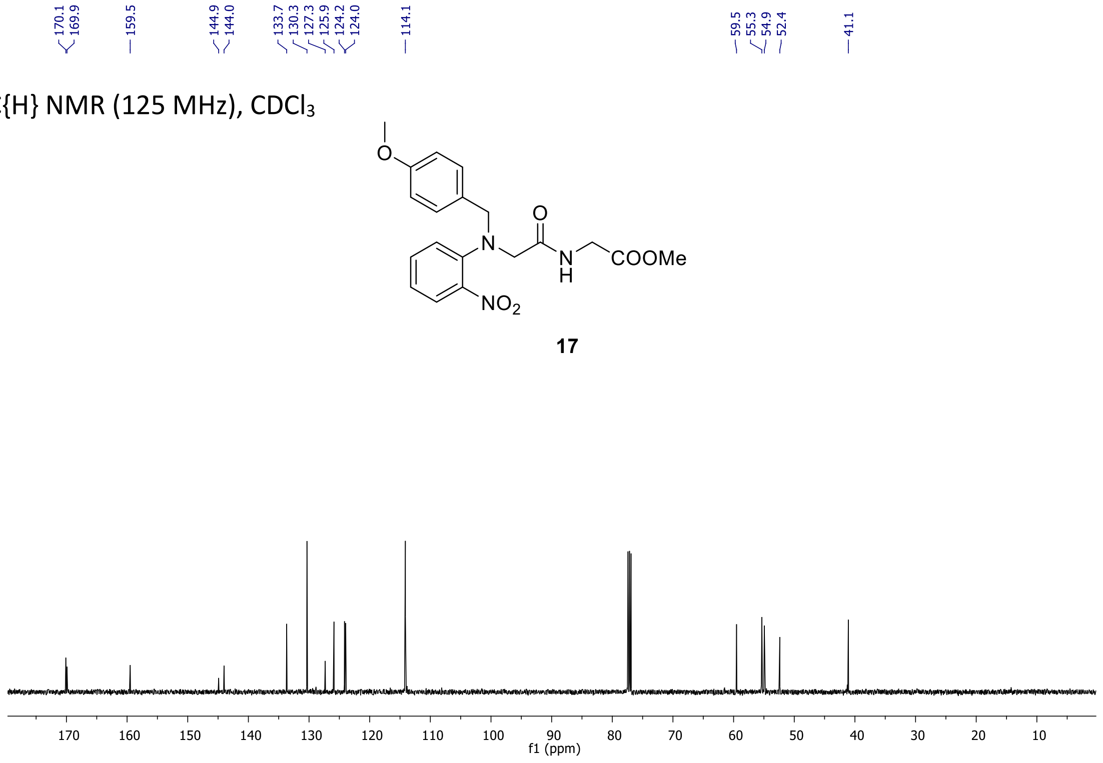

^{1}H NMR (500 MHz), $CDCl_3$

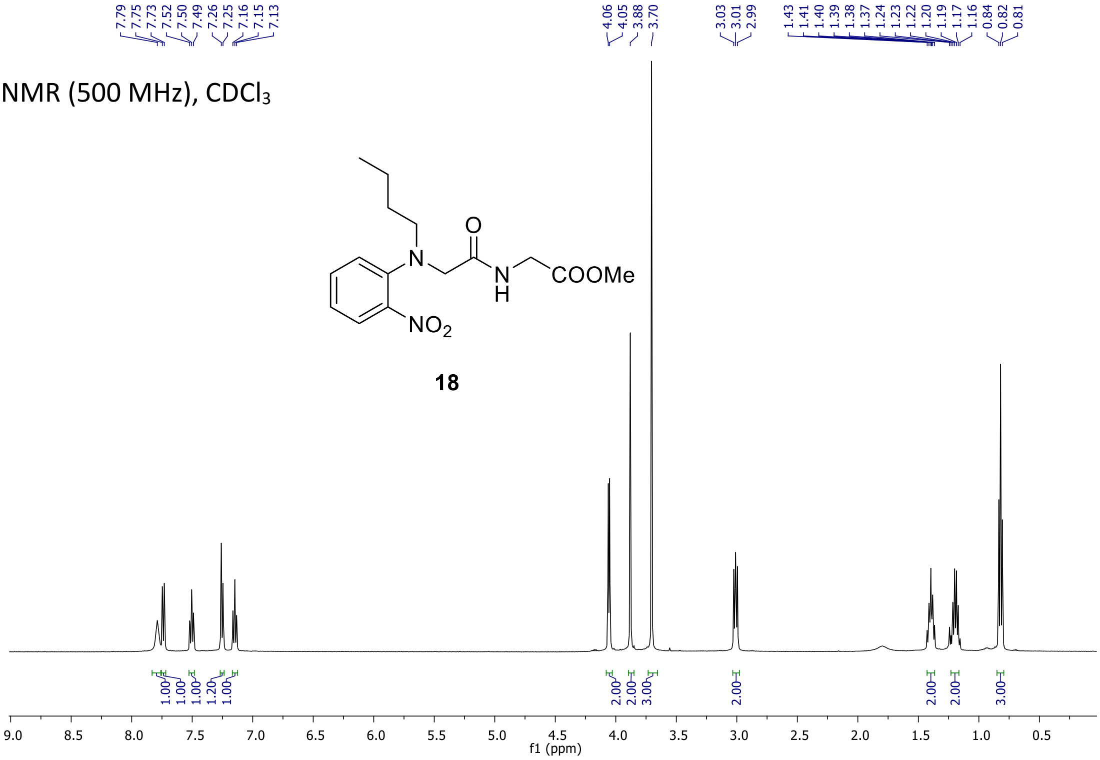

$^{13}C\{H\}$ NMR (125 MHz), $CDCl_3$

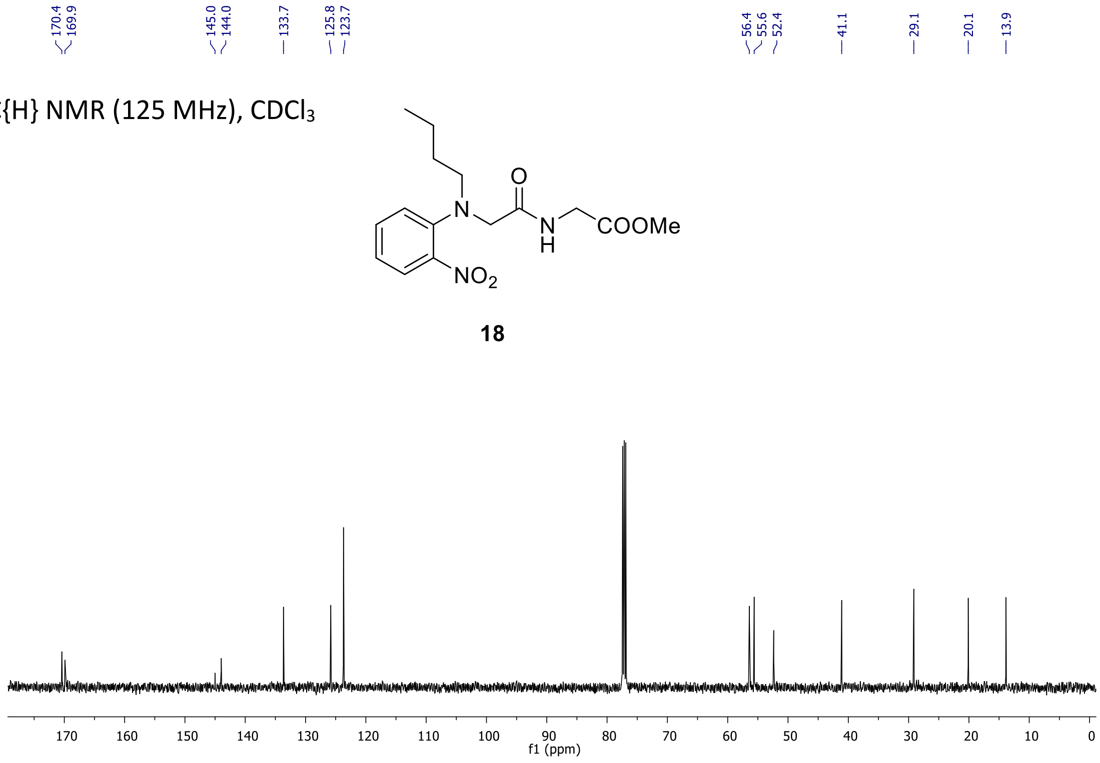

^{1}H NMR (500 MHz), $CDCl_3$

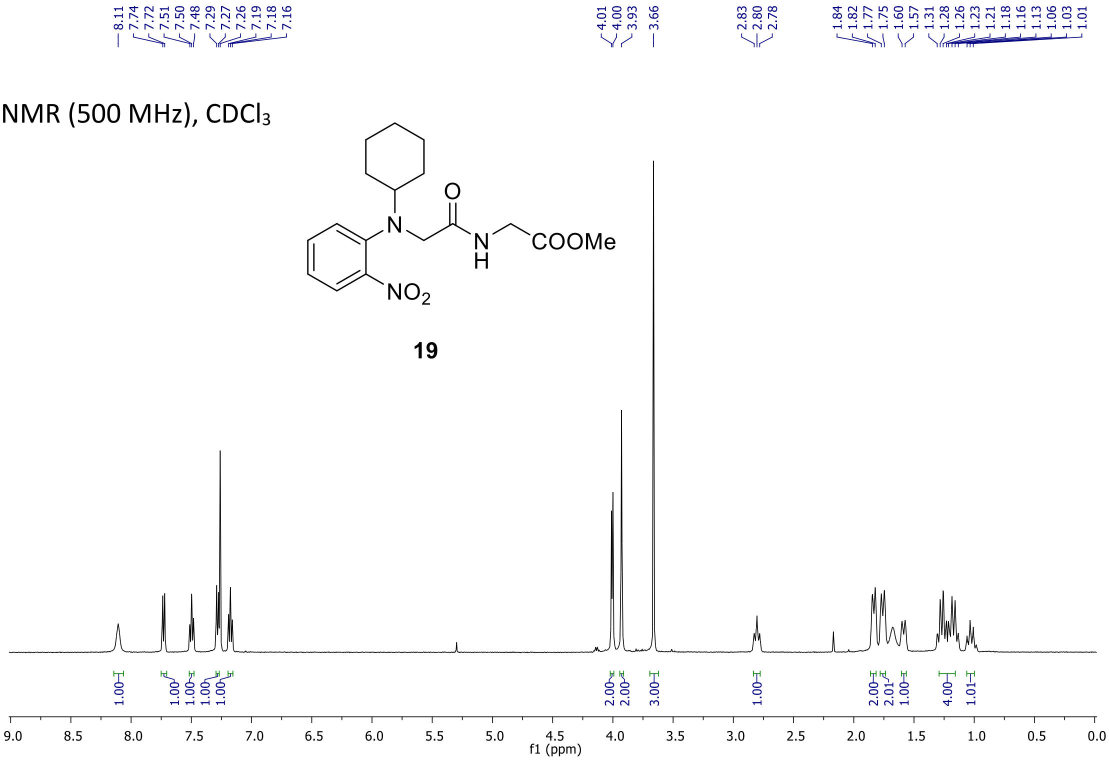

19

^{13}C{H} NMR (125 MHz), $CDCl_3$

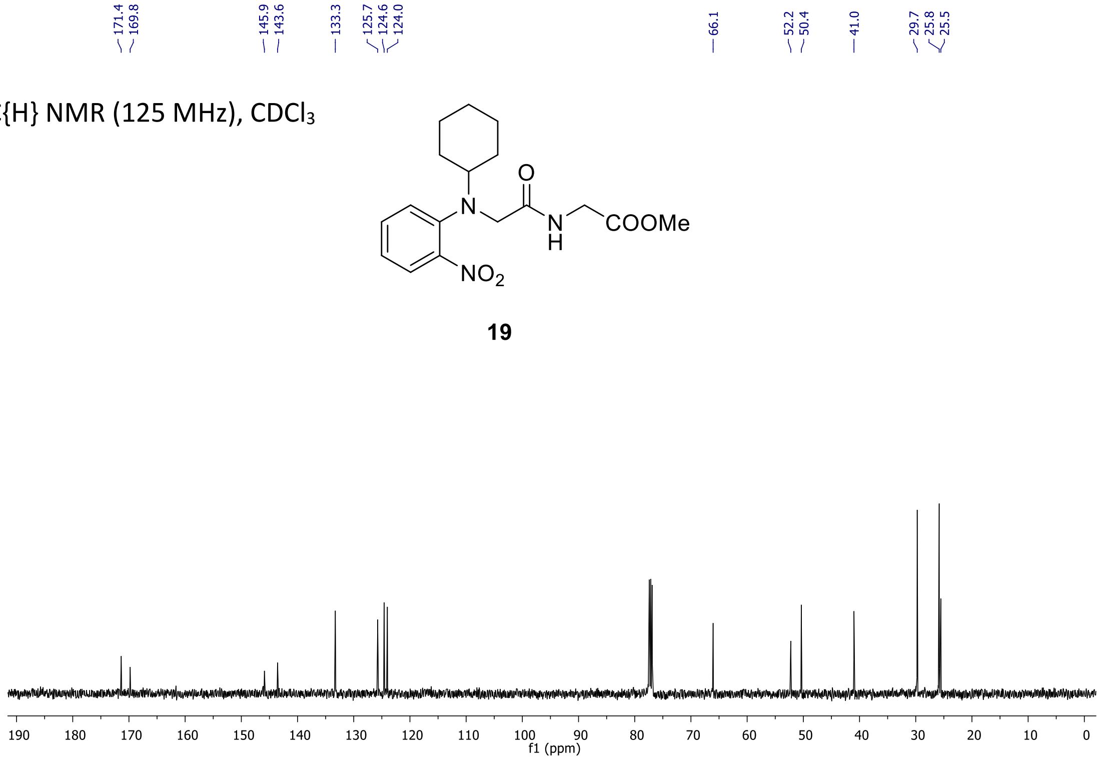

19

^{1}H NMR (500 MHz), $CDCl_3$

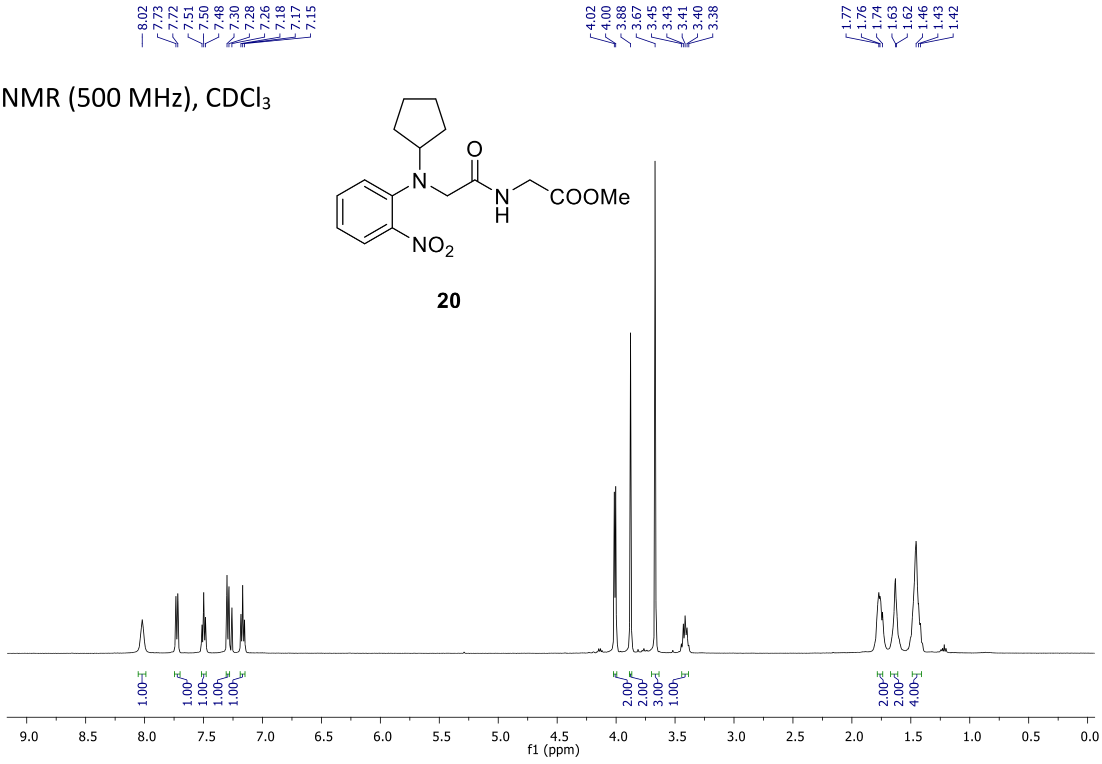

$^{13}C\{H\}$ NMR (125 MHz), $CDCl_3$

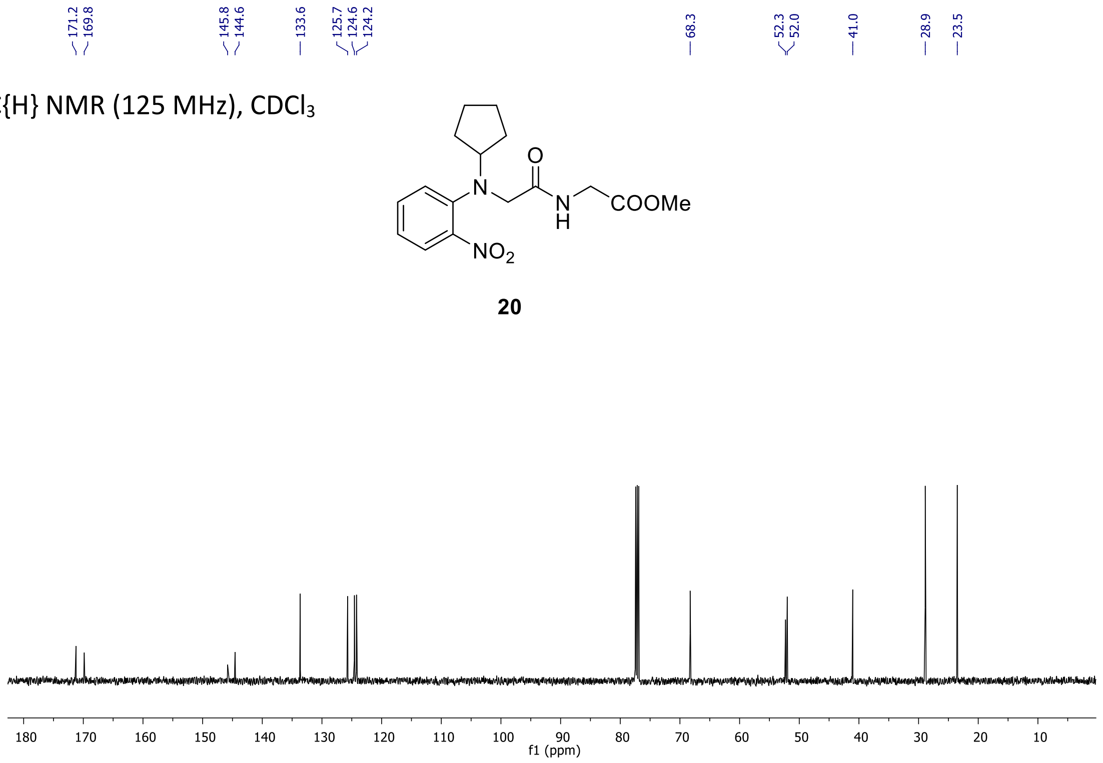

^{1}H NMR (500 MHz), $CDCl_3$

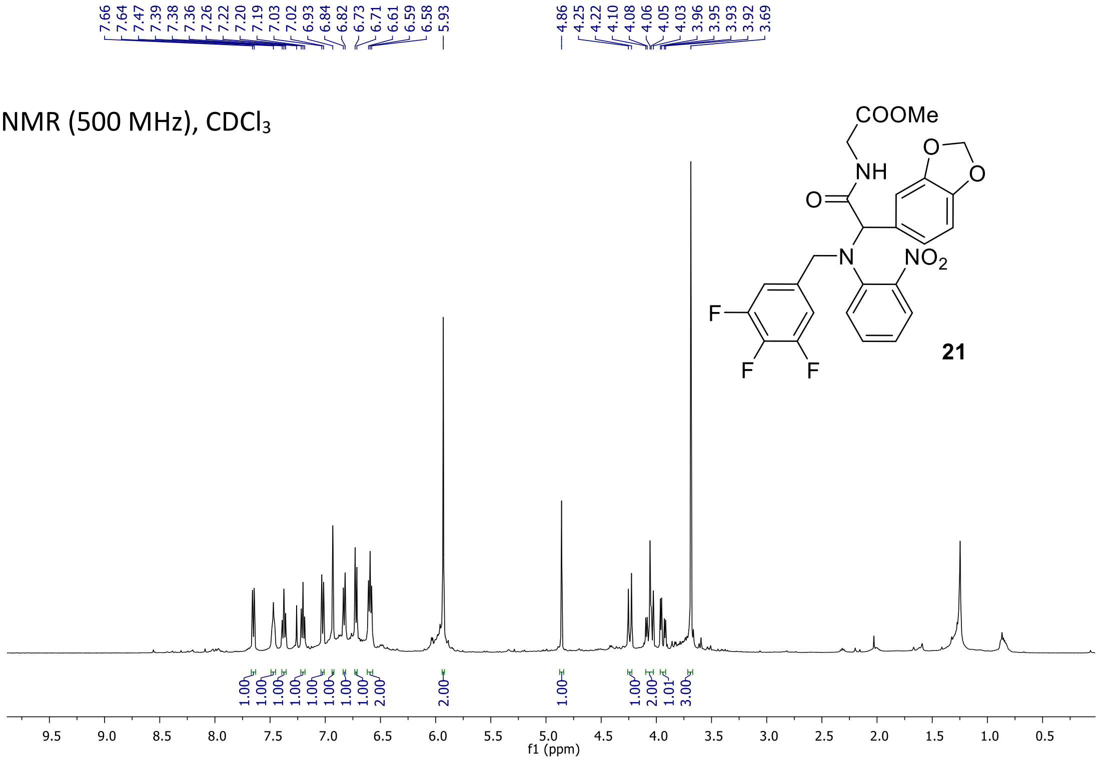

^{13}C{H} NMR (125 MHz), $CDCl_3$

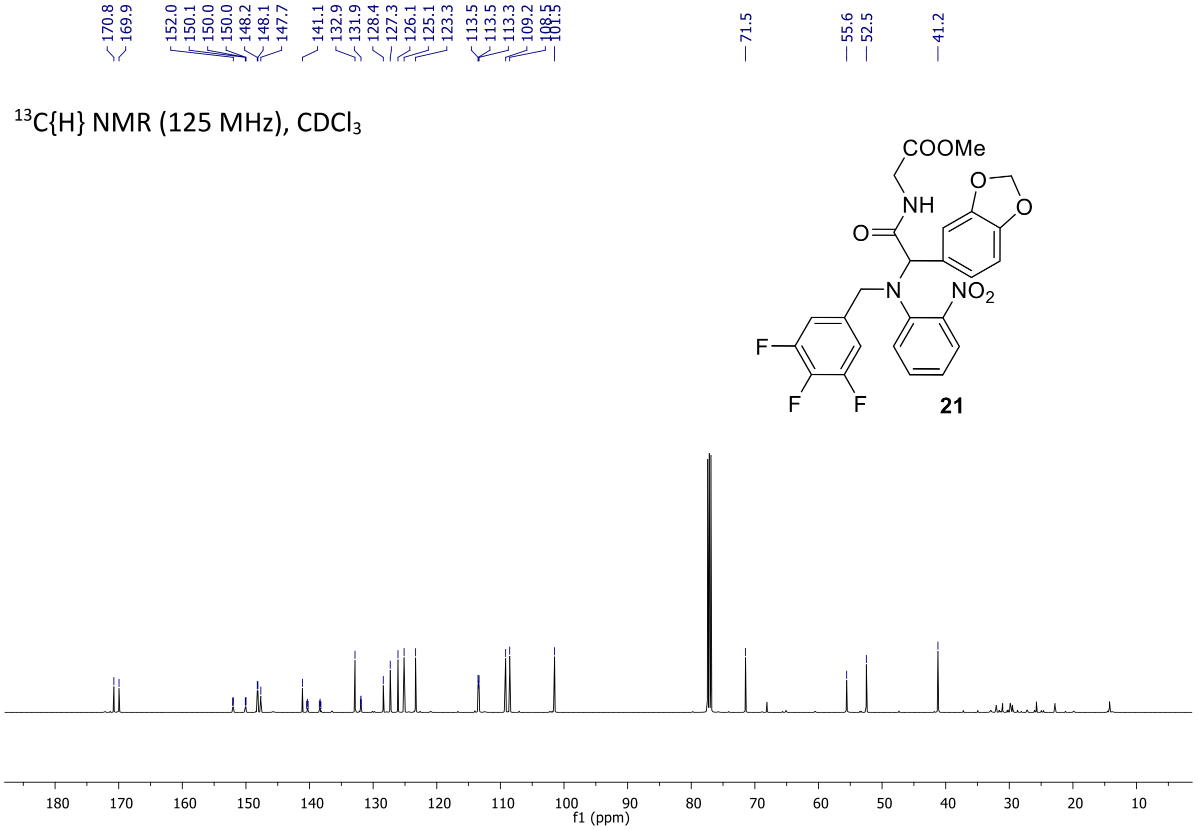

^{19}F NMR (188 MHz) of the compound 21

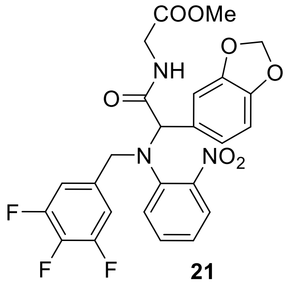

21

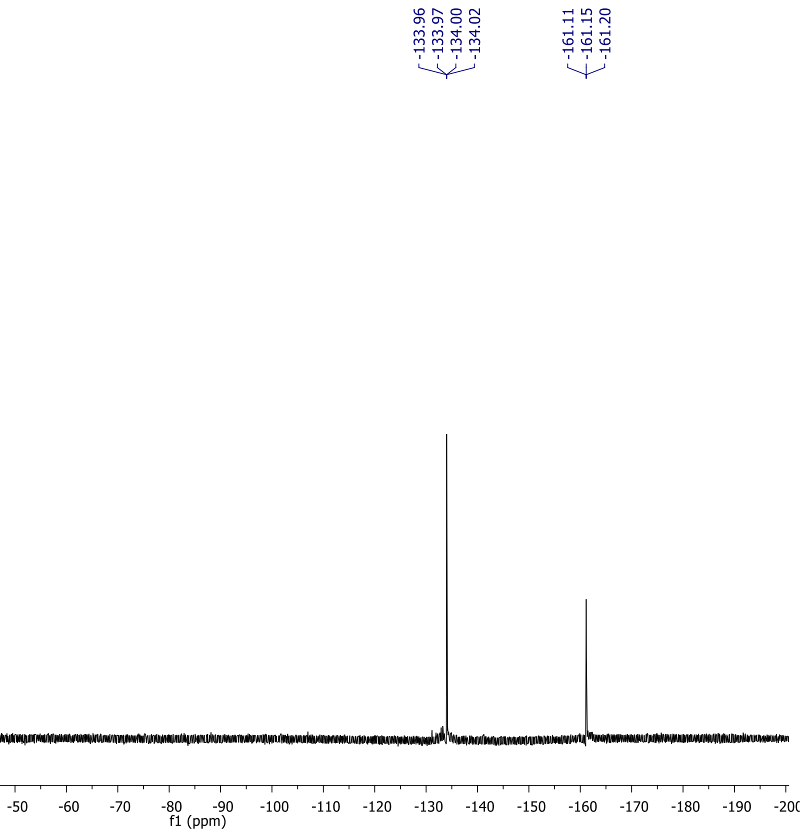

Copies of ^{1}H and ^{13}C NMR spectra of amines 1a-21a

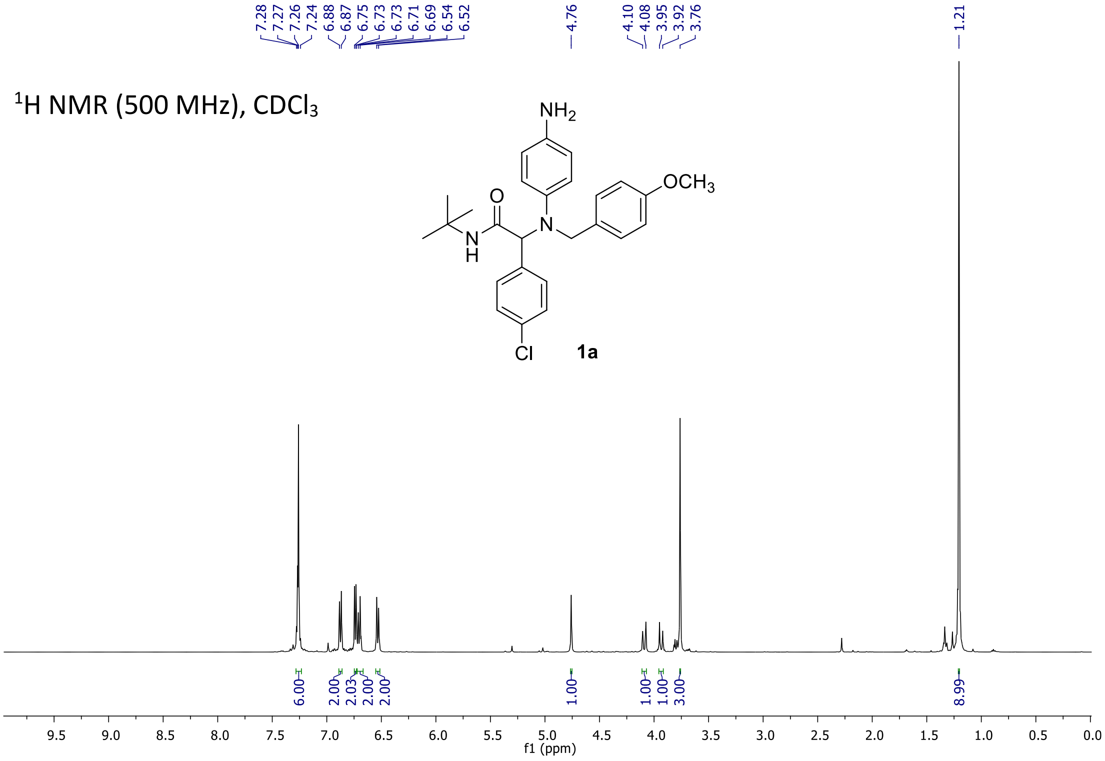

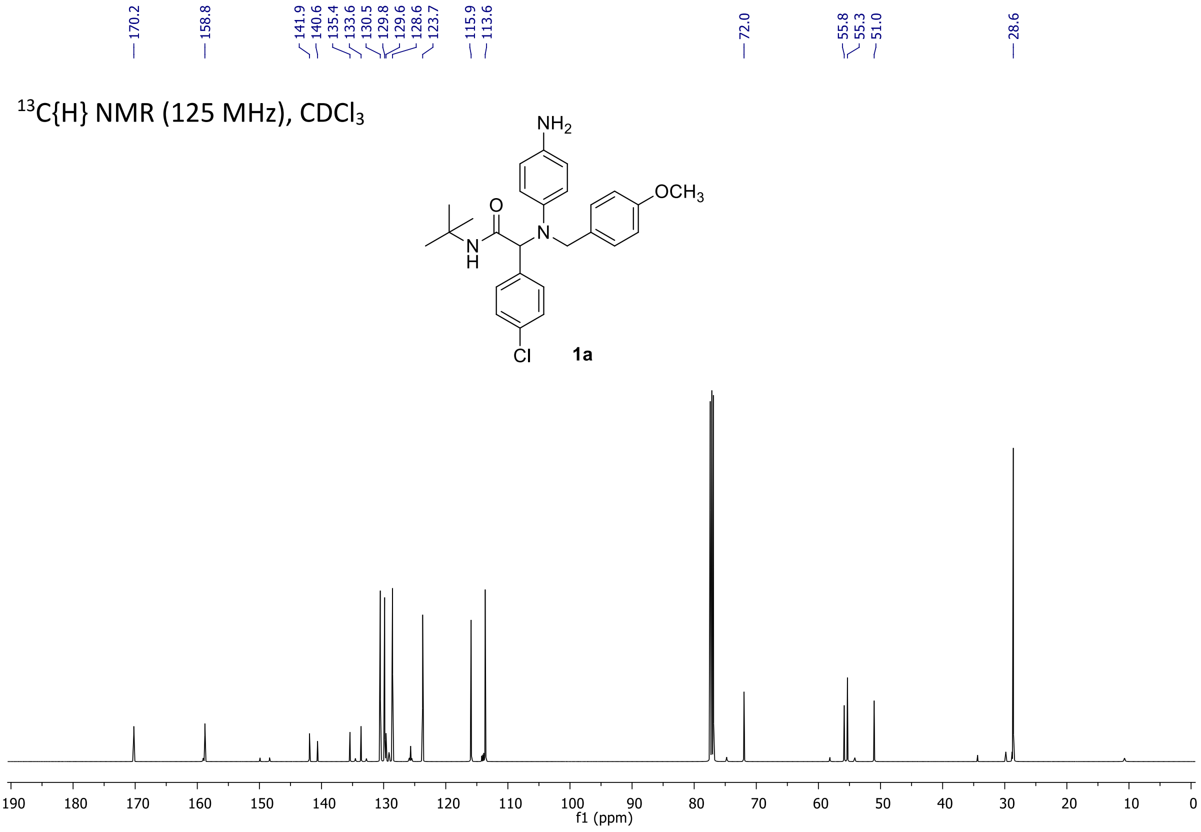

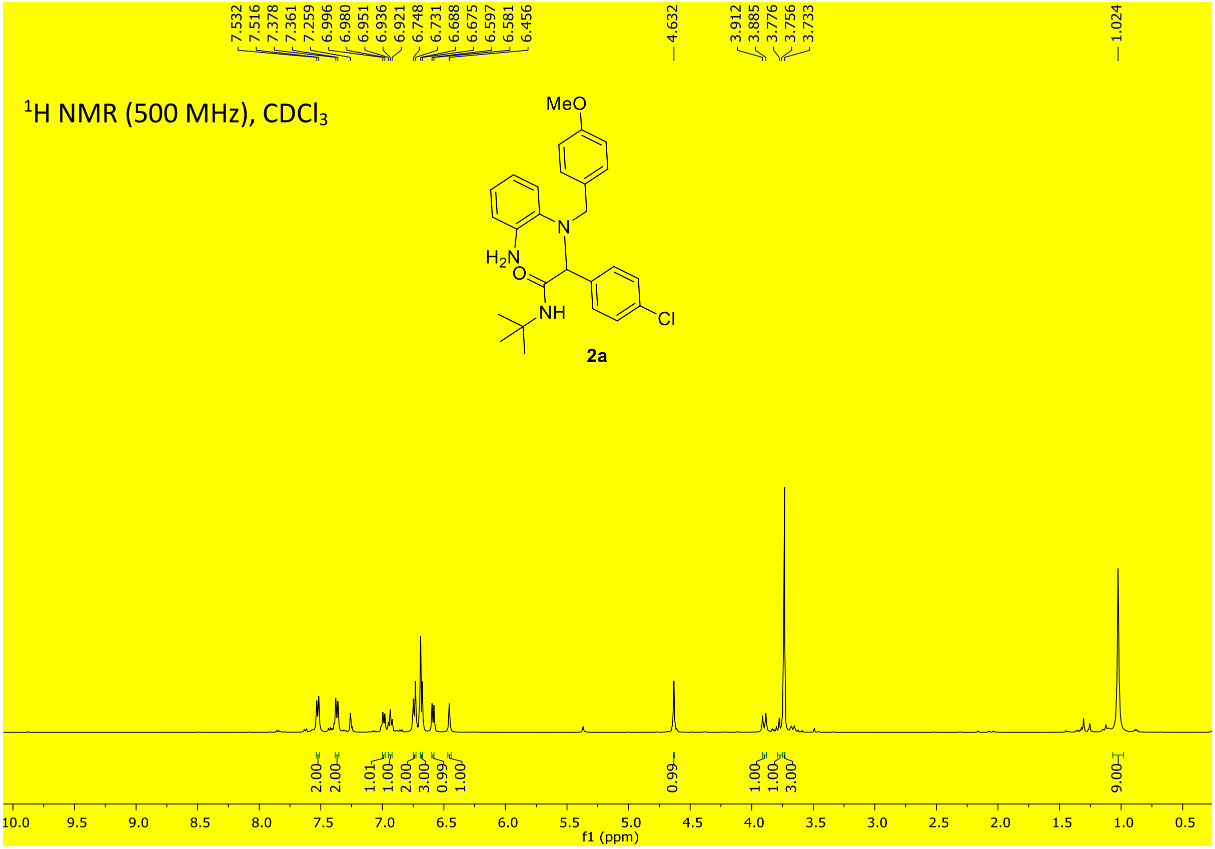
7.532
7.516
7.378
7.361
7.259
6.996
6.980
6.951
6.936
6.921
6.748
6.731
6.688
6.675
6.597
6.581
6.456
4.632
3.912
3.885
3.776
3.756
3.733
1.024
^{1}H NMR (500 MHz), CDCl$_3$
MeO
N
H_2N
O
NH
Cl
2a
2.00
2.00
1.01
1.00
2.00
3.00
0.99
1.00
0.99
1.00
1.00
3.00
9.00
f1 (ppm)

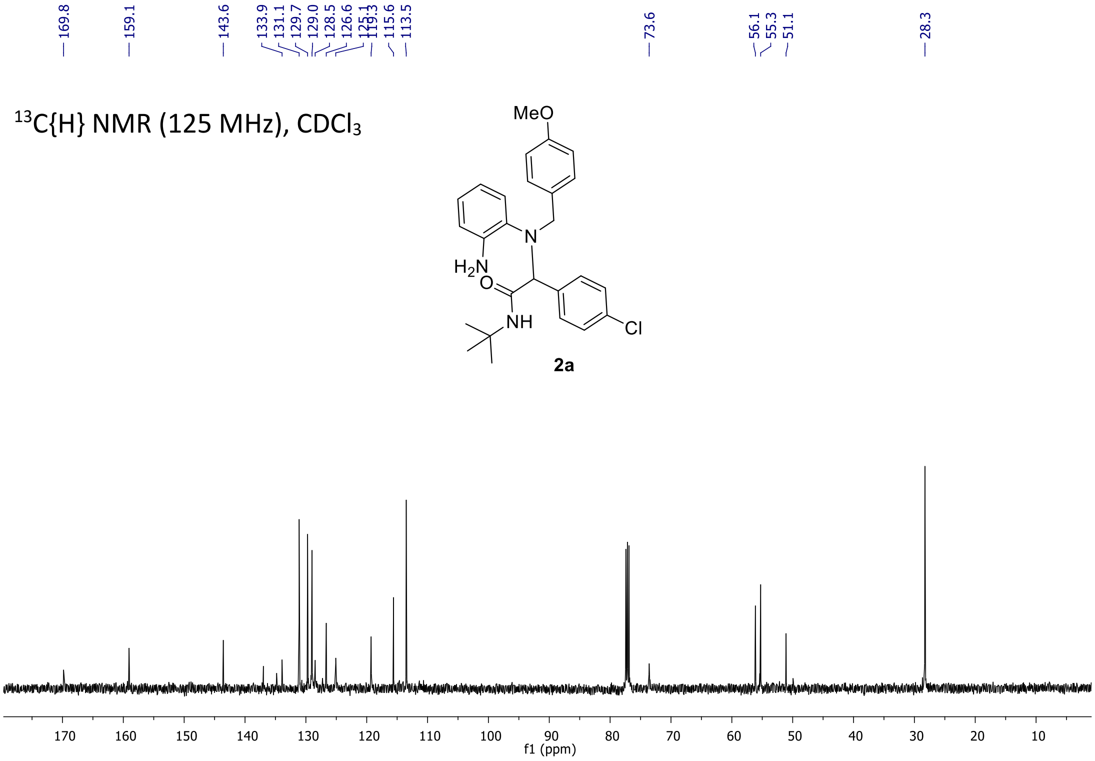
169.8
159.1
143.6
133.9
131.1
129.7
129.0
128.5
126.6
125.1
119.3
115.6
113.5
73.6
56.1
55.3
51.1
28.3
^{13}C{H} NMR (125 MHz), CDCl$_3$
MeO
N
H_2N
O
NH
Cl
2a
f1 (ppm)

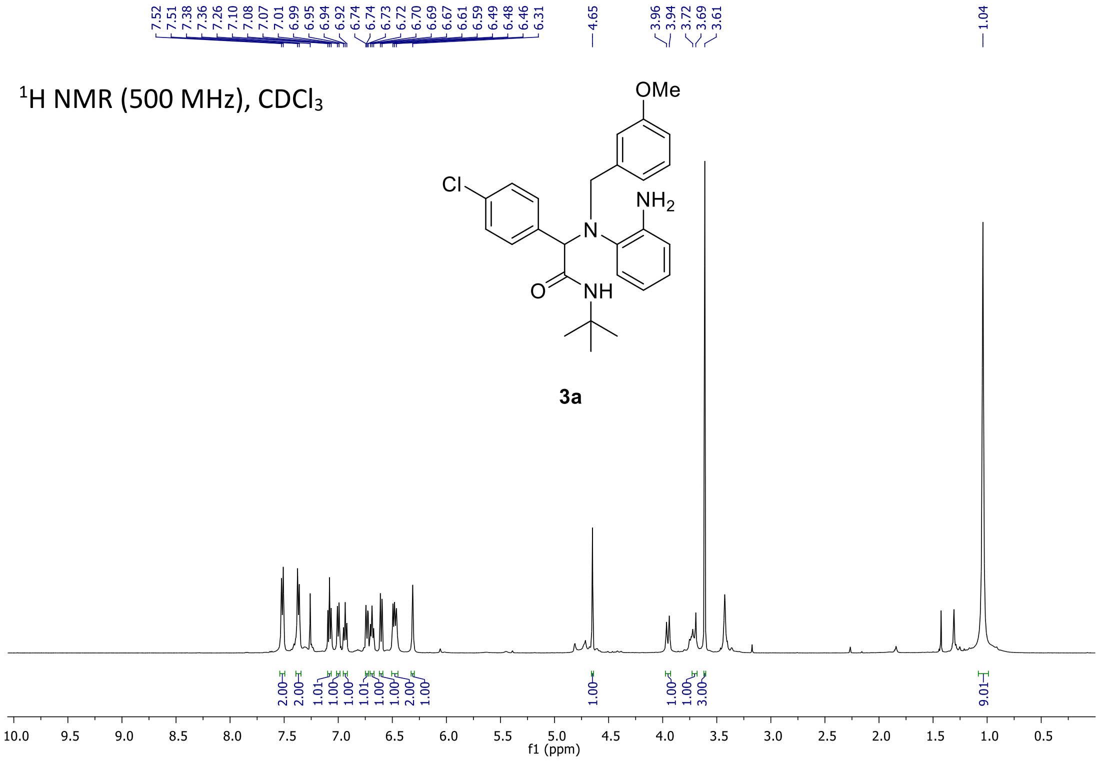
7.52
7.51
7.38
7.36
7.26
7.10
7.08
7.07
7.01
6.99
6.95
6.94
6.92
6.74
6.74
6.73
6.72
6.70
6.69
6.67
6.61
6.59
6.49
6.48
6.46
6.31
4.65
3.96
3.94
3.72
3.69
3.61
1.04
^{1}H NMR (500 MHz), $CDCl_3$
OMe
Cl
NH_2
N
O
NH
3a
2.00
2.00
1.01
1.00
1.00
1.01
1.00
1.00
2.00
1.00
1.00
1.00
1.00
3.00
9.01
f1 (ppm)

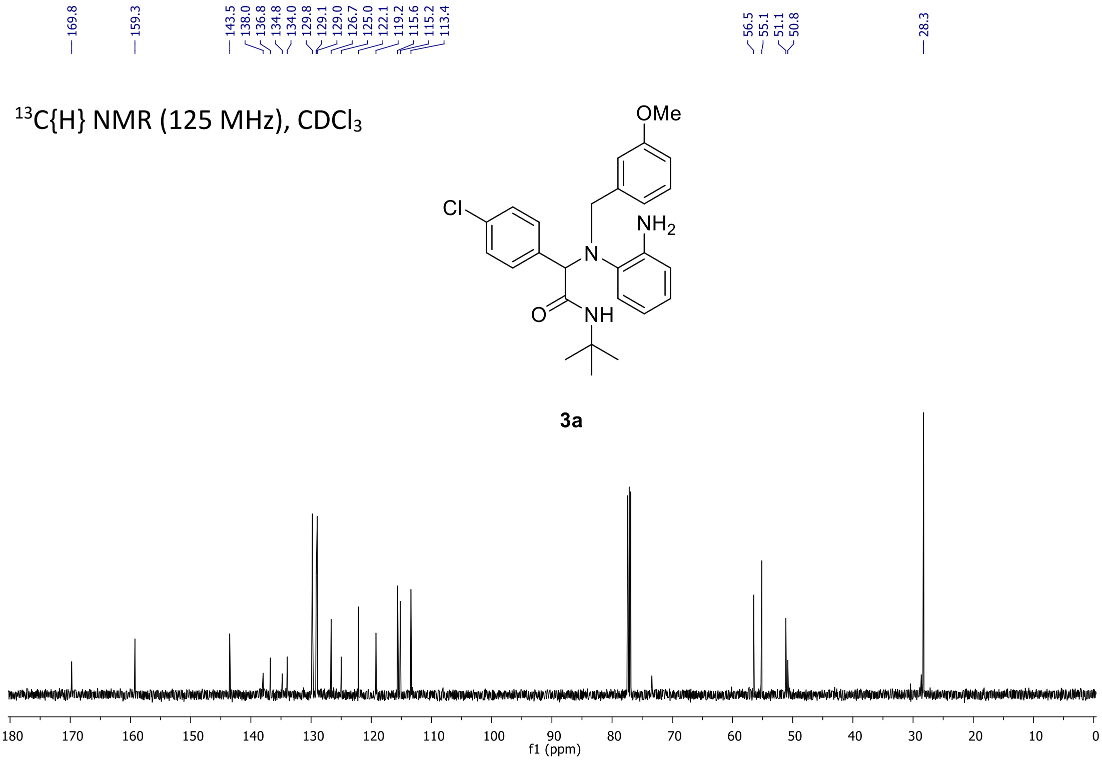
169.8
159.3
143.5
138.0
136.8
134.8
134.0
129.8
129.1
129.0
126.7
125.0
122.1
119.2
115.6
115.2
113.4
56.5
55.1
51.1
50.8
28.3
^{13}C{H} NMR (125 MHz), $CDCl_3$
OMe
Cl
NH_2
N
O
NH
3a
f1 (ppm)

^{1}H NMR (500 MHz), $CDCl_3$

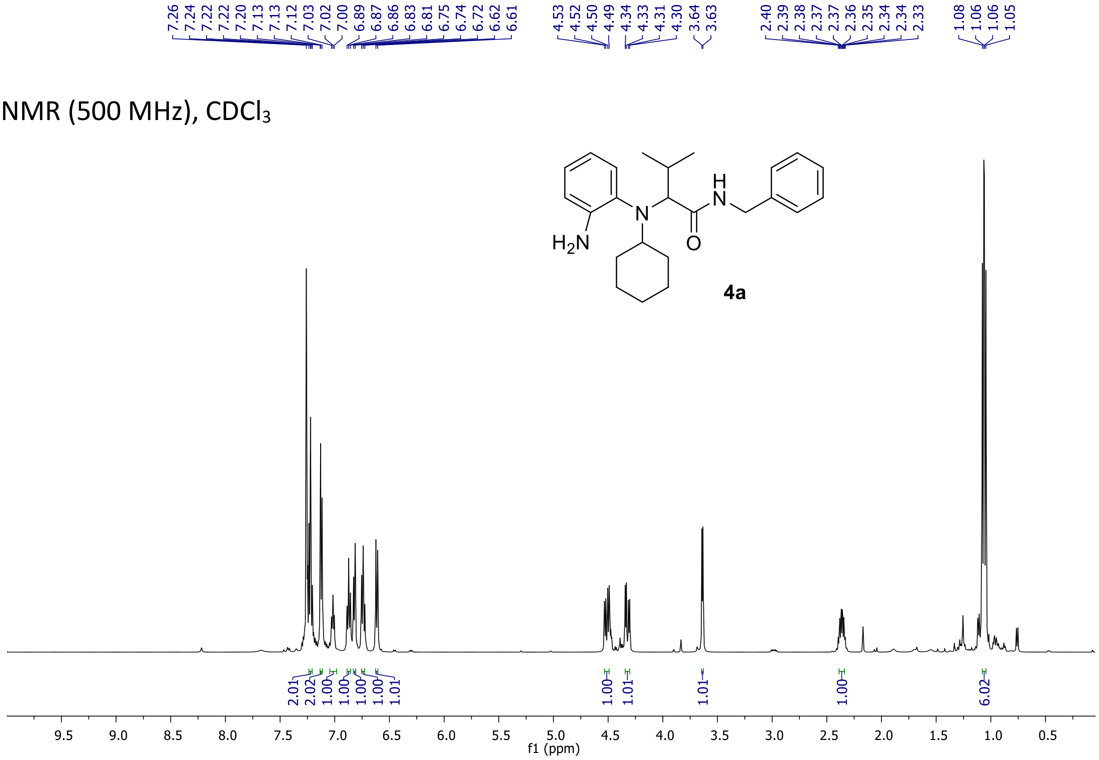

$^{13}C\{H\}$ NMR (125 MHz), $CDCl_3$

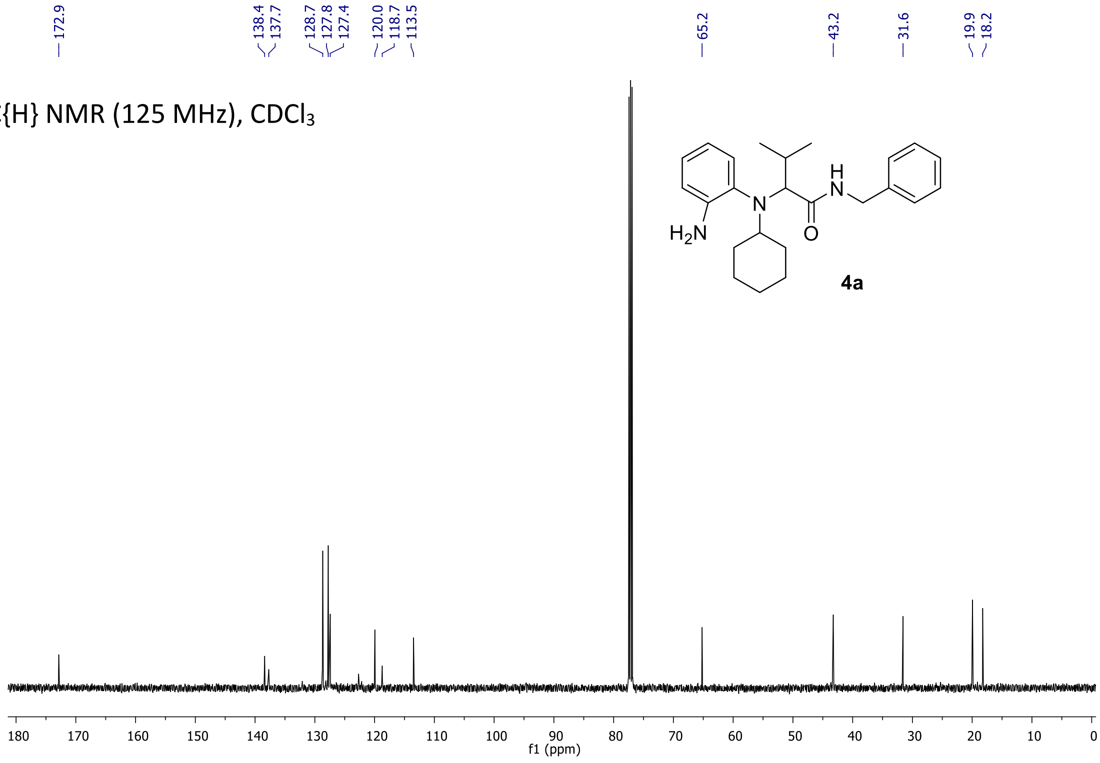

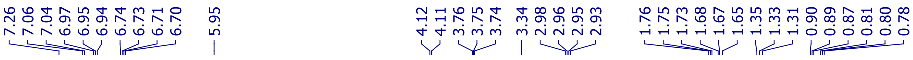

^{1}H NMR (500 MHz), $CDCl_3$

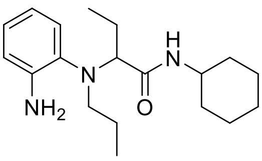

5a

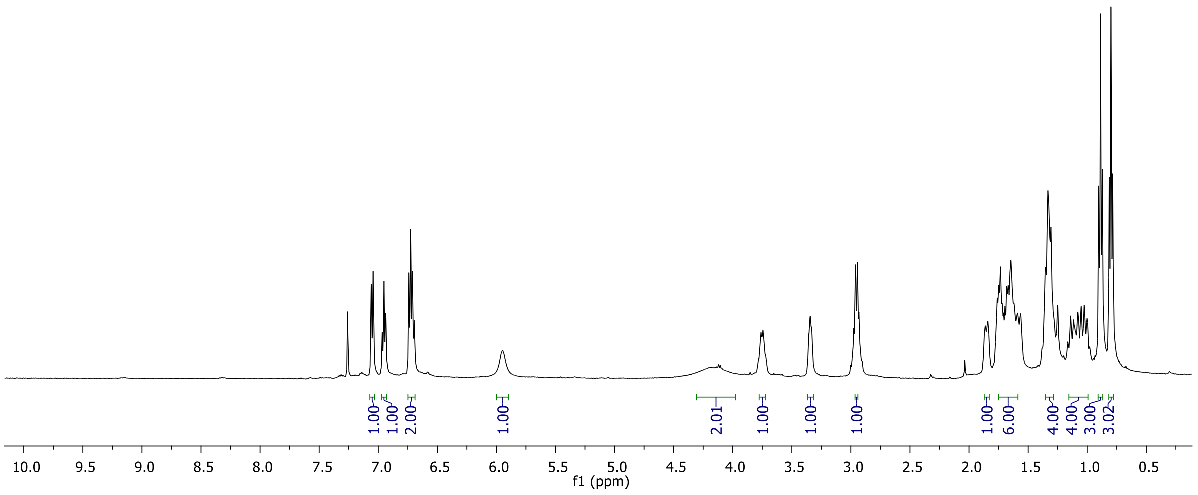

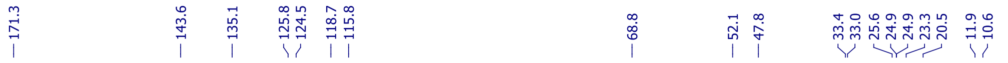

$^{13}C\{H\}$ NMR (125 MHz), $CDCl_3$

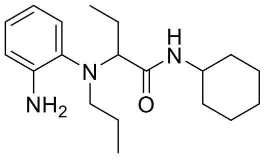

5a

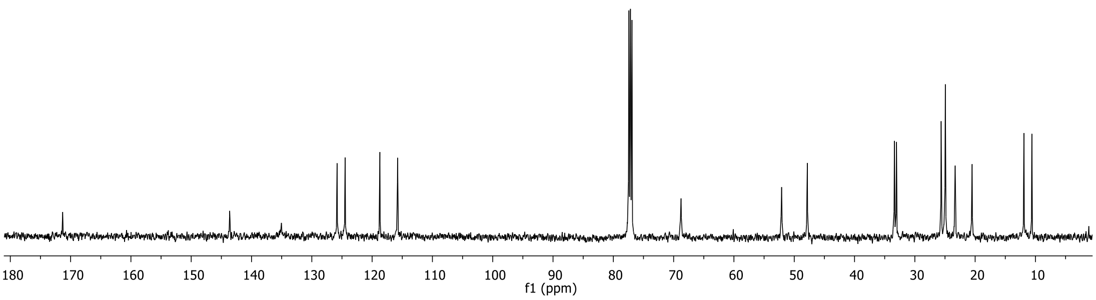

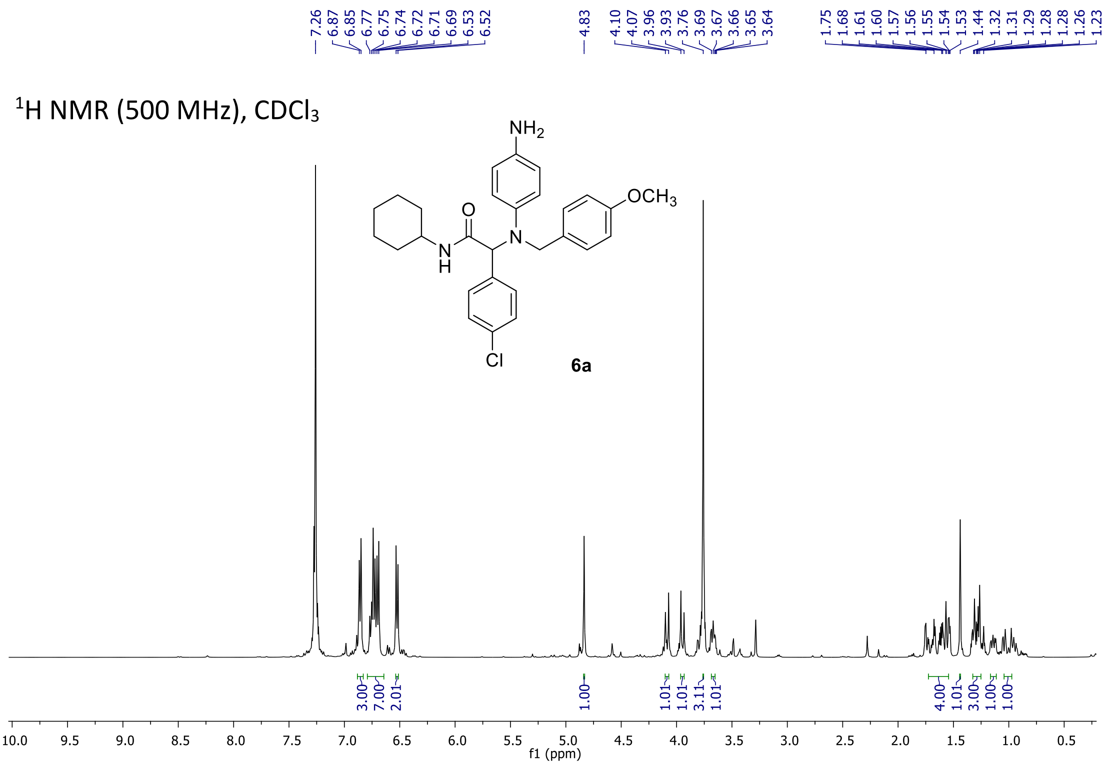
7.26
6.87
6.85
6.77
6.75
6.74
6.72
6.71
6.69
6.53
6.52
4.83
4.10
4.07
3.96
3.93
3.76
3.69
3.67
3.66
3.65
3.64
1.75
1.68
1.61
1.60
1.57
1.56
1.55
1.54
1.53
1.44
1.32
1.31
1.29
1.28
1.28
1.26
1.23
^{1}H NMR (500 MHz), CDCl$_3$
NH$_2$
OCH$_3$
O
N
N
H
Cl
6a
3.00
7.00
2.01
1.00
1.01
1.01
3.11
1.01
4.00
1.01
3.00
1.00
1.00
10.0 9.5 9.0 8.5 8.0 7.5 7.0 6.5 6.0 5.5 5.0 4.5 4.0 3.5 3.0 2.5 2.0 1.5 1.0 0.5
f1 (ppm)

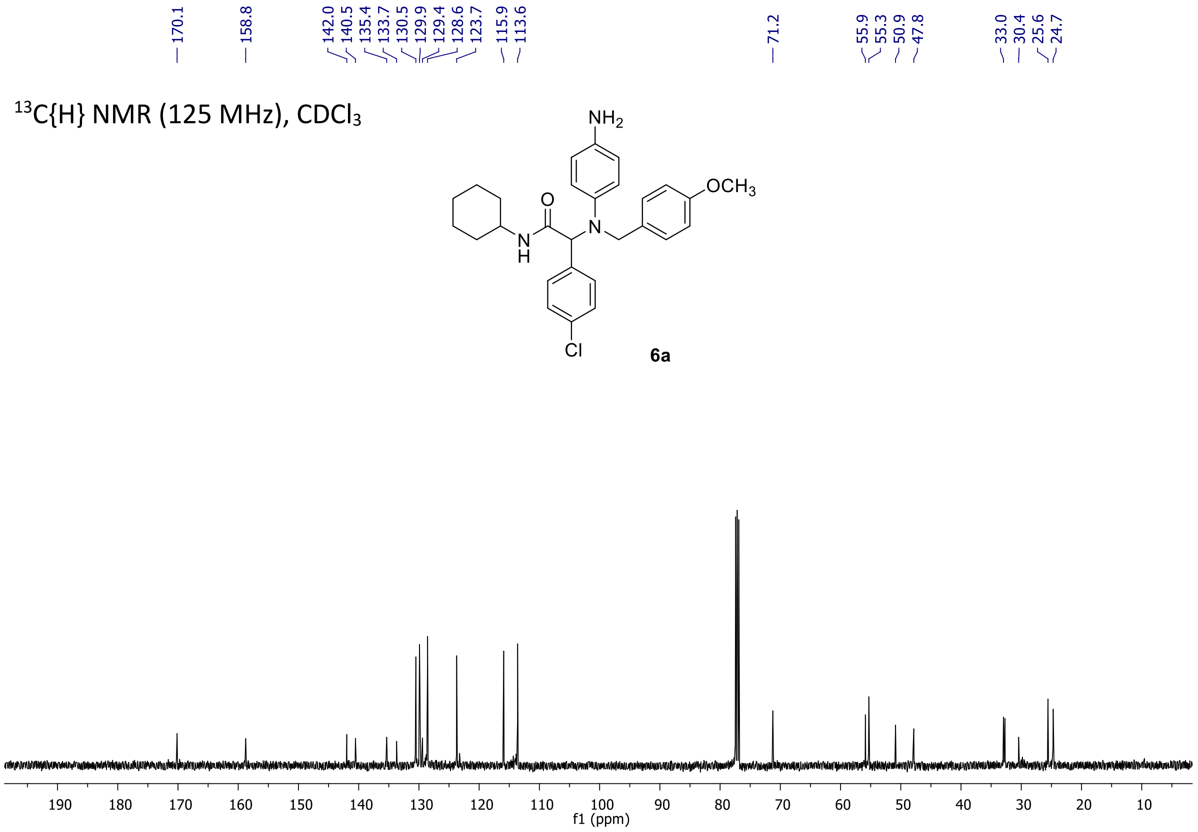
170.1
158.8
142.0
140.5
135.4
133.7
130.5
129.9
129.4
128.6
123.7
115.9
113.6
71.2
55.9
55.3
50.9
47.8
33.0
30.4
25.6
24.7
^{13}C{H} NMR (125 MHz), CDCl$_3$
NH$_2$
OCH$_3$
O
N
N
H
Cl
6a
190 180 170 160 150 140 130 120 110 100 90 80 70 60 50 40 30 20 10
f1 (ppm)

¹H NMR (500 MHz), CDCl$_3$

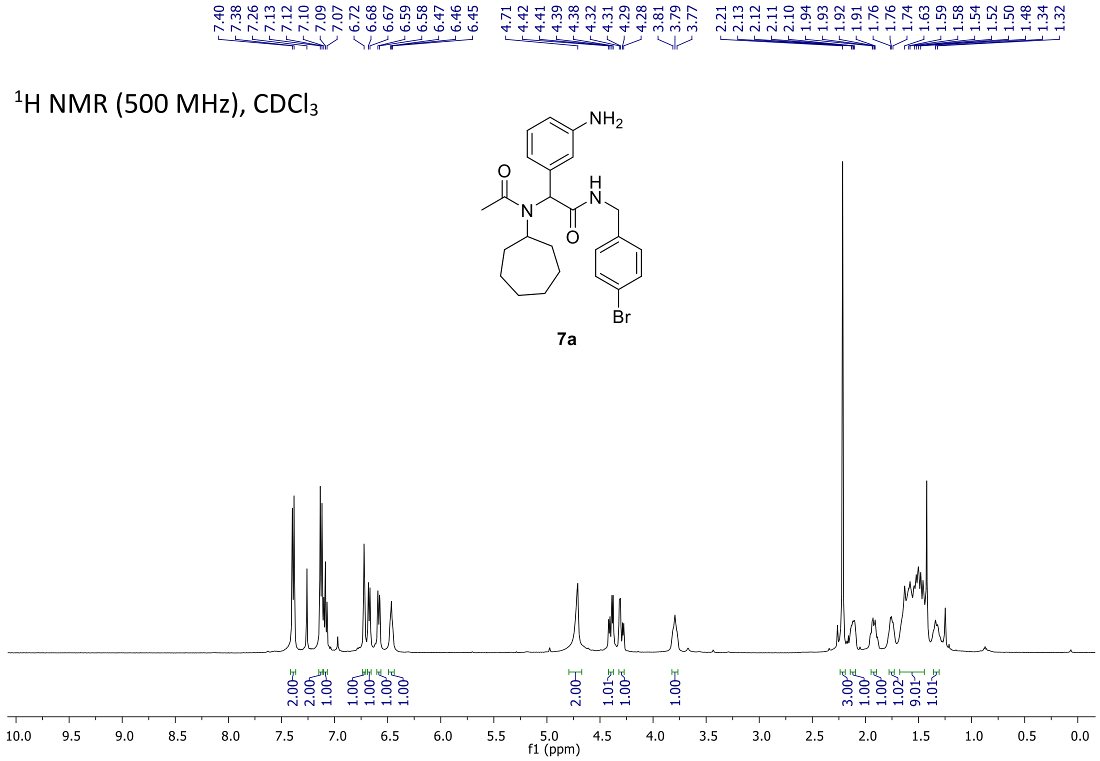

¹³C{H} NMR (125 MHz), CDCl$_3$

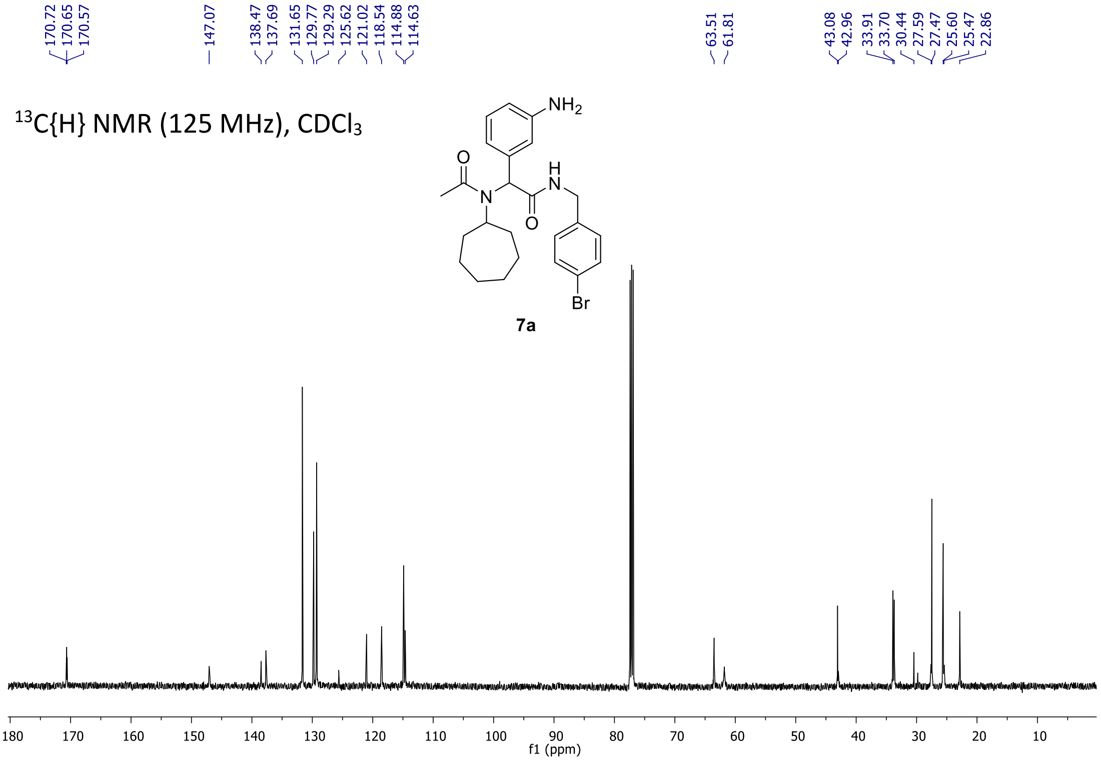

^{1}H NMR (500 MHz), $CDCl_3$

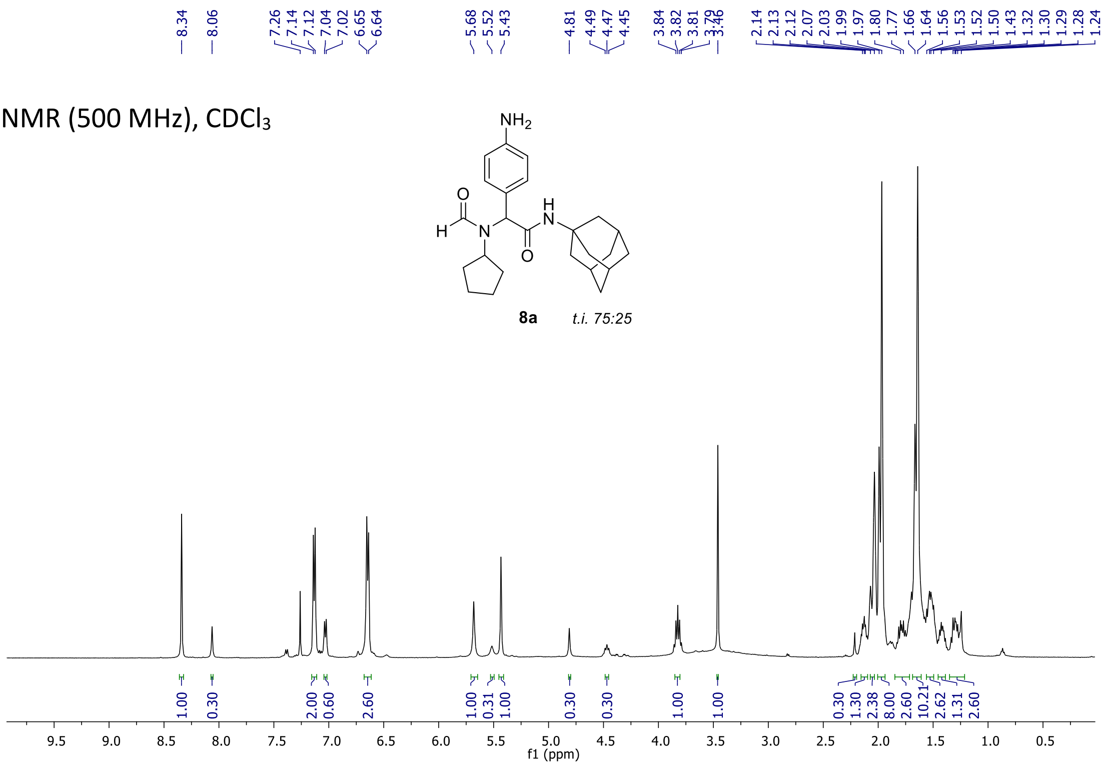

$^{13}C\{H\}$ NMR (125 MHz), $CDCl_3$

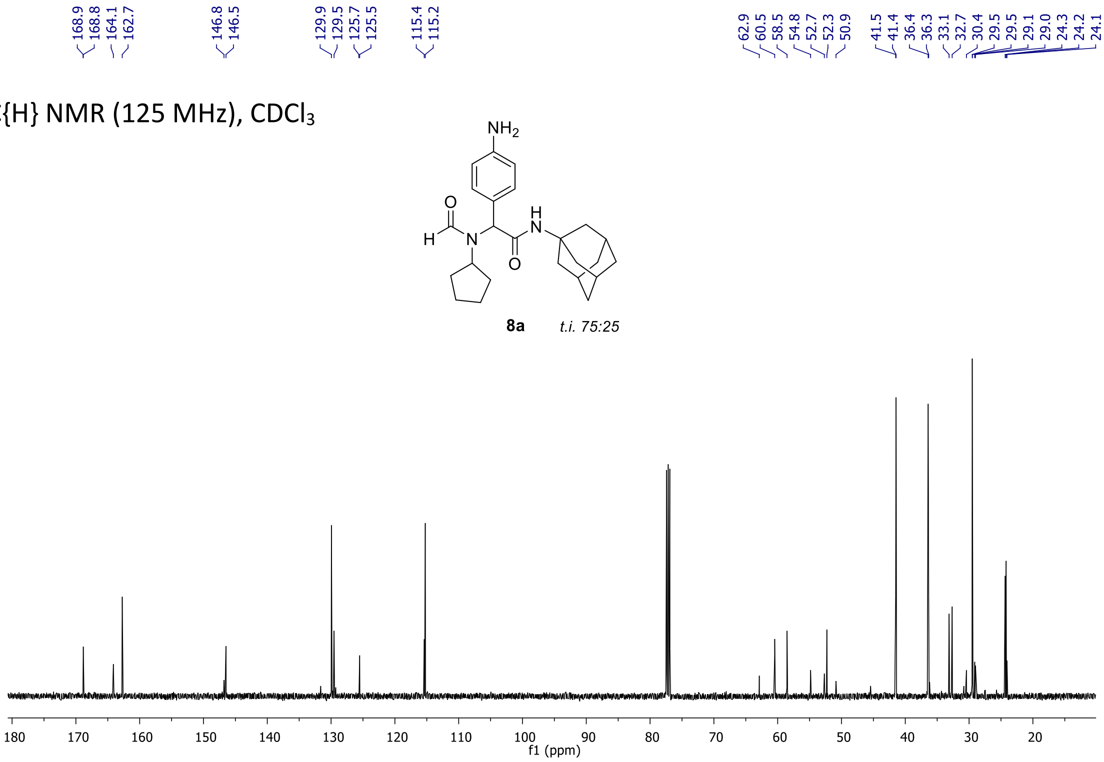

t.i.-topo isomers

^{1}H NMR (500 MHz), $CDCl_3$

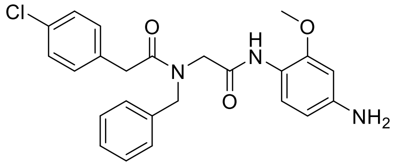

9a

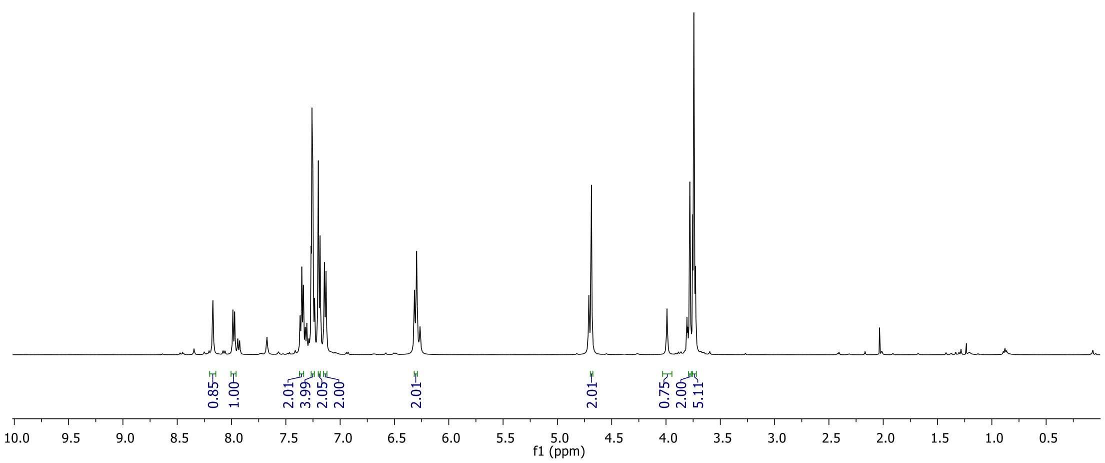

$^{13}C\{H\}$ NMR (125 MHz), $CDCl_3$

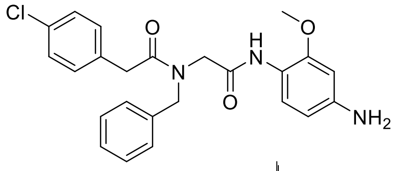

9a

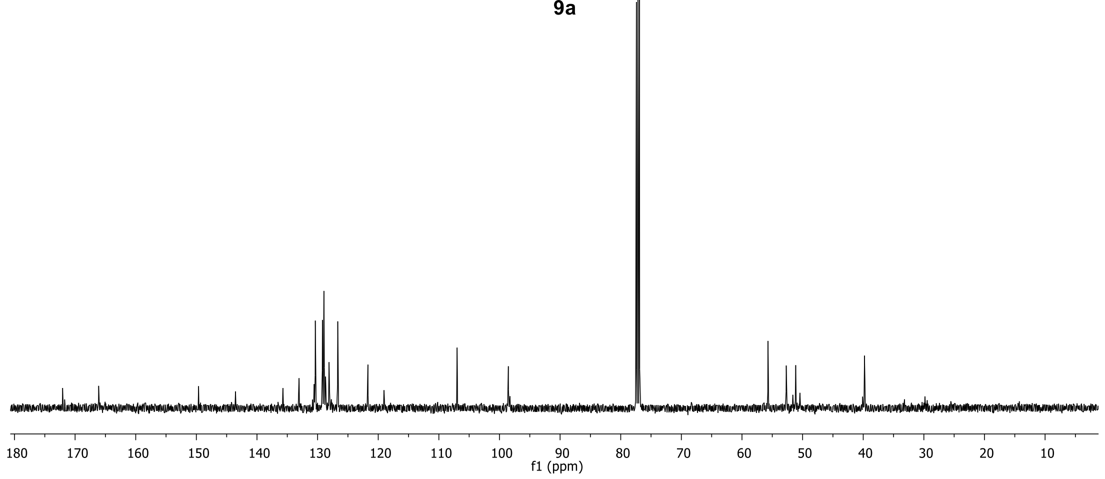

^{1}H NMR (500 MHz), $CDCl_3$

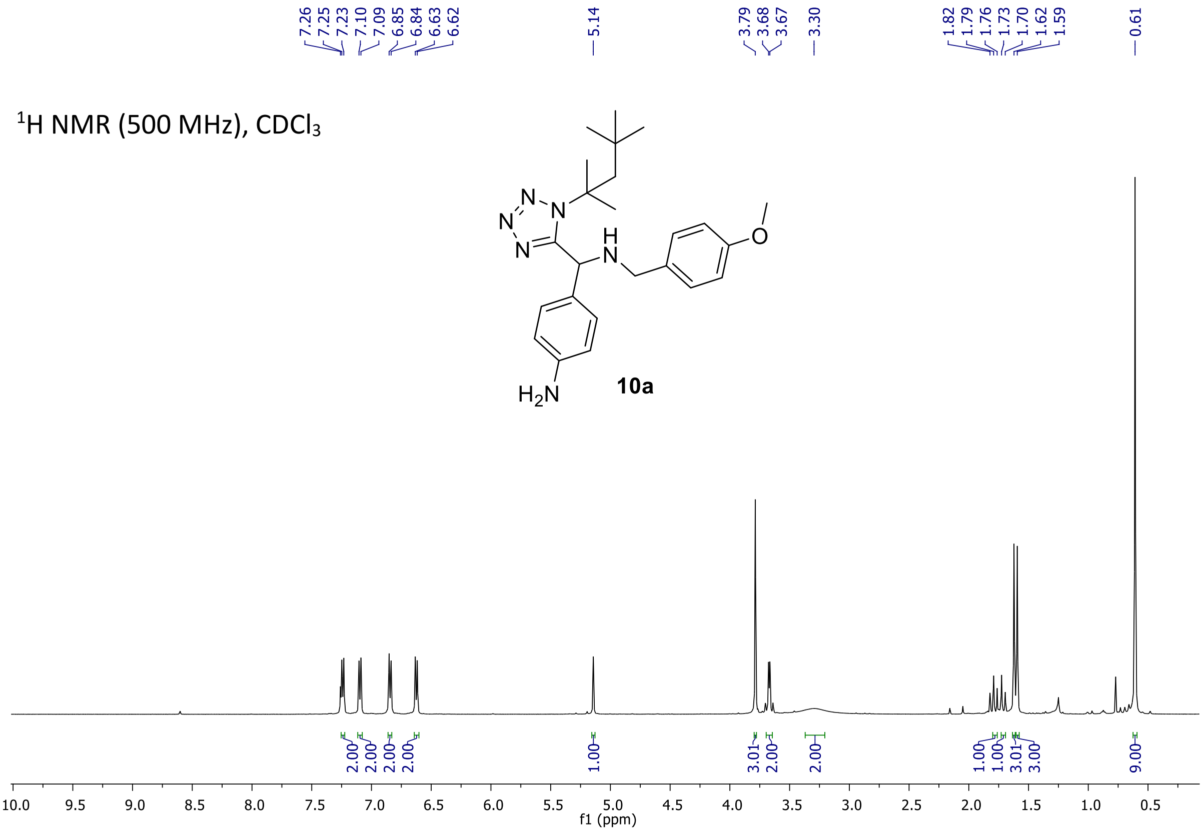

$^{13}C\{H\}$ NMR (125 MHz), $CDCl_3$

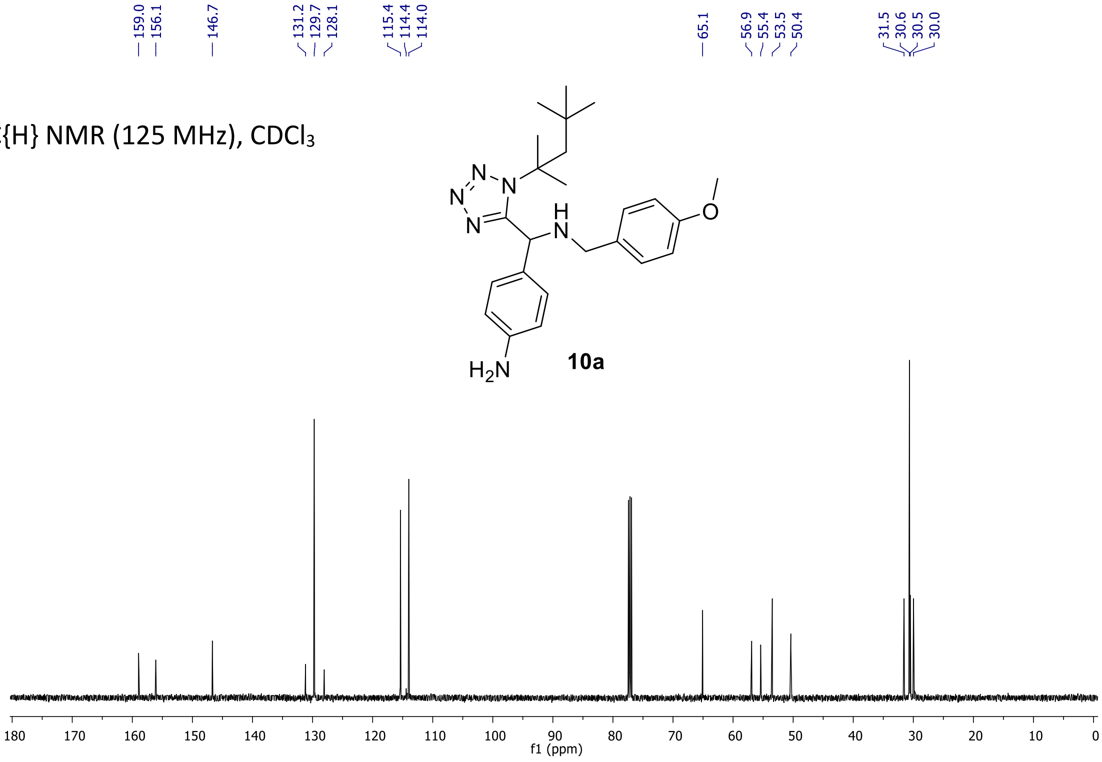

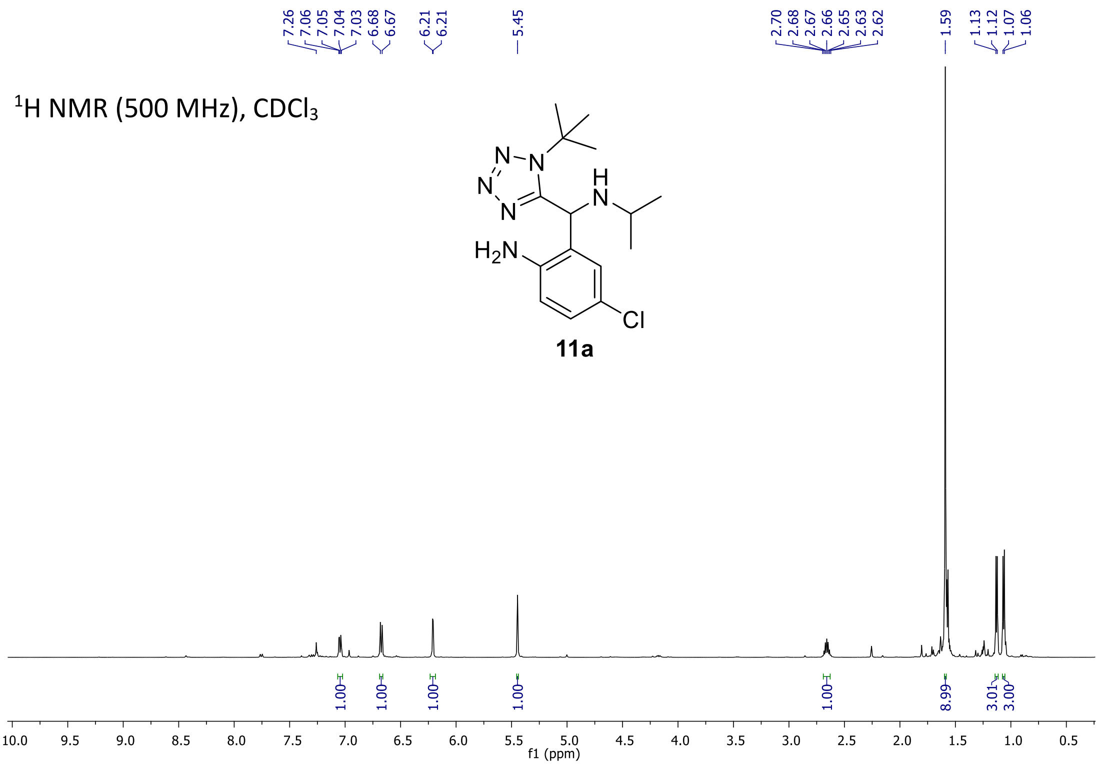
7.26
7.06
7.05
7.04
7.03
6.68
6.67
6.21
6.21
5.45
2.70
2.68
2.67
2.66
2.65
2.63
2.62
1.59
1.13
1.12
1.07
1.06
^{1}H NMR (500 MHz), $CDCl_3$
11a
1.00
1.00
1.00
1.00
1.00
8.99
3.01
3.00
f1 (ppm)

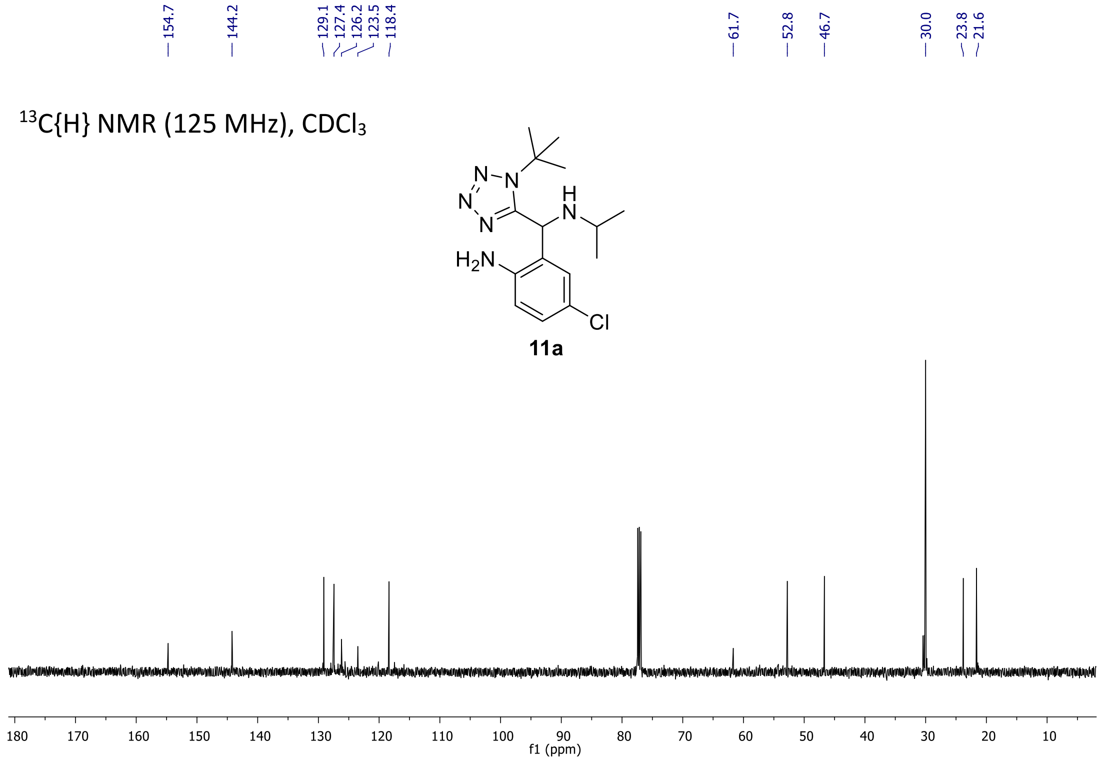
154.7
144.2
129.1
127.4
126.2
123.5
118.4
61.7
52.8
46.7
30.0
23.8
21.6
^{13}C{H} NMR (125 MHz), $CDCl_3$
11a
f1 (ppm)

^{1}H NMR (500 MHz), $CDCl_3$

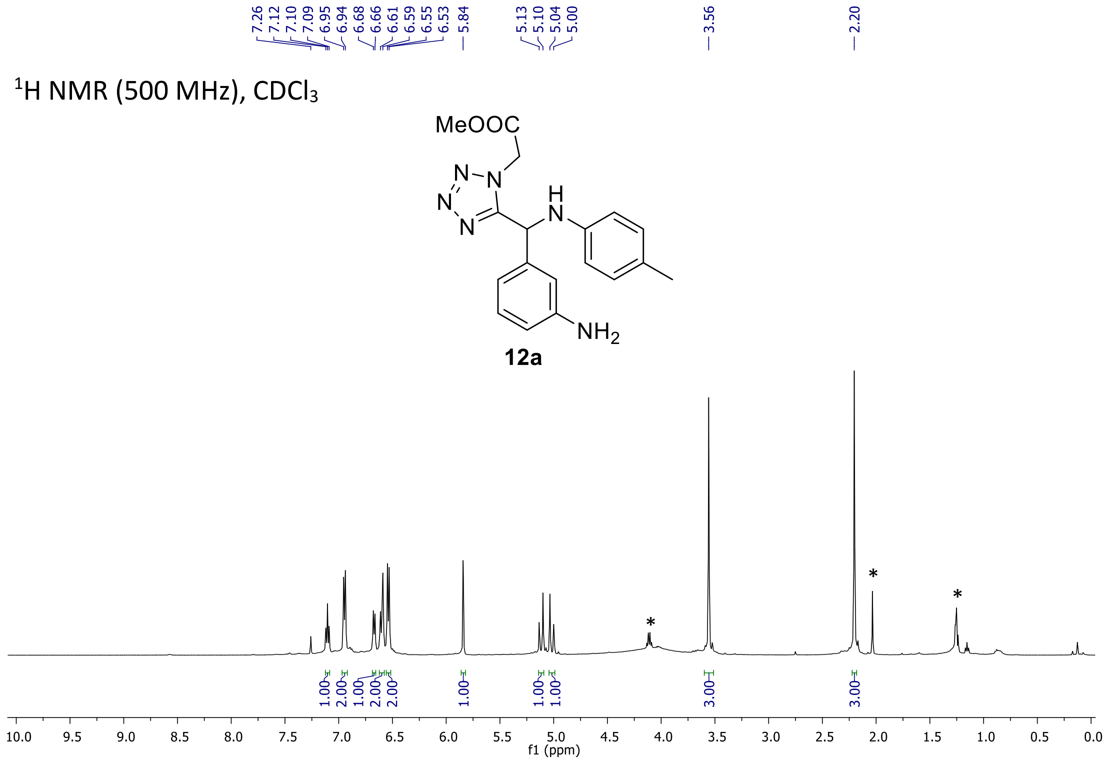

12a

^{13}C{H} NMR (125 MHz), $CDCl_3$

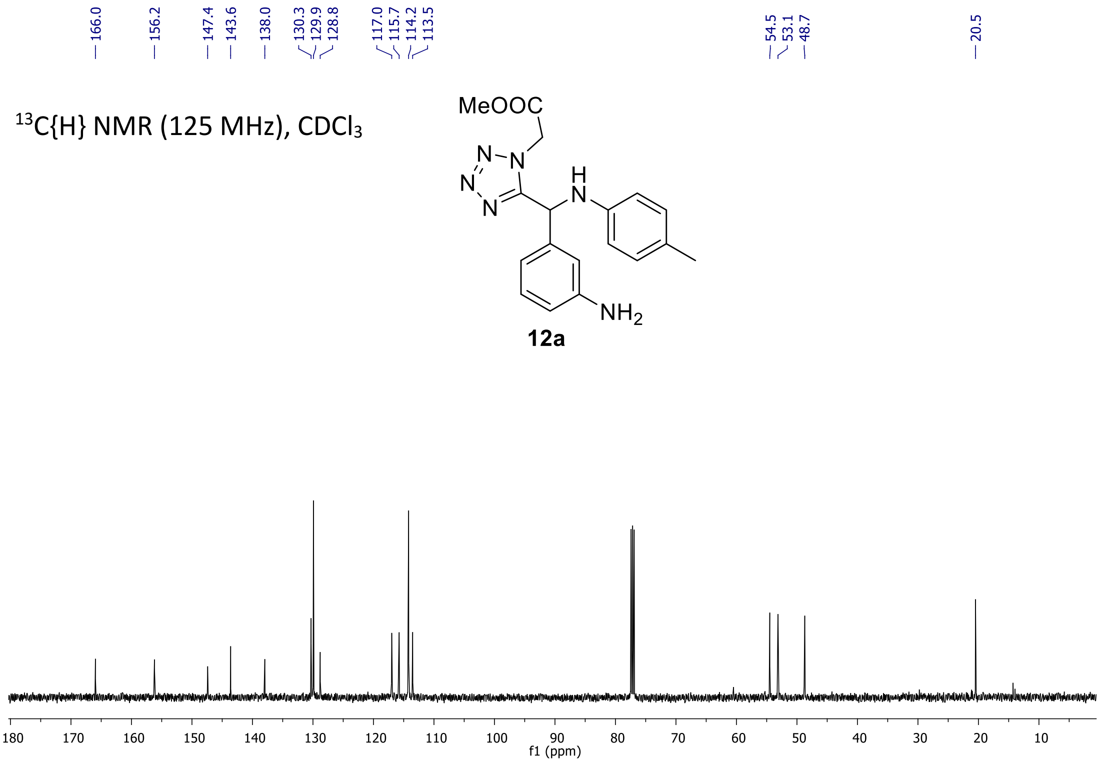

12a

*Ethyl acetate.

^{1}H NMR (500 MHz), $CDCl_3$

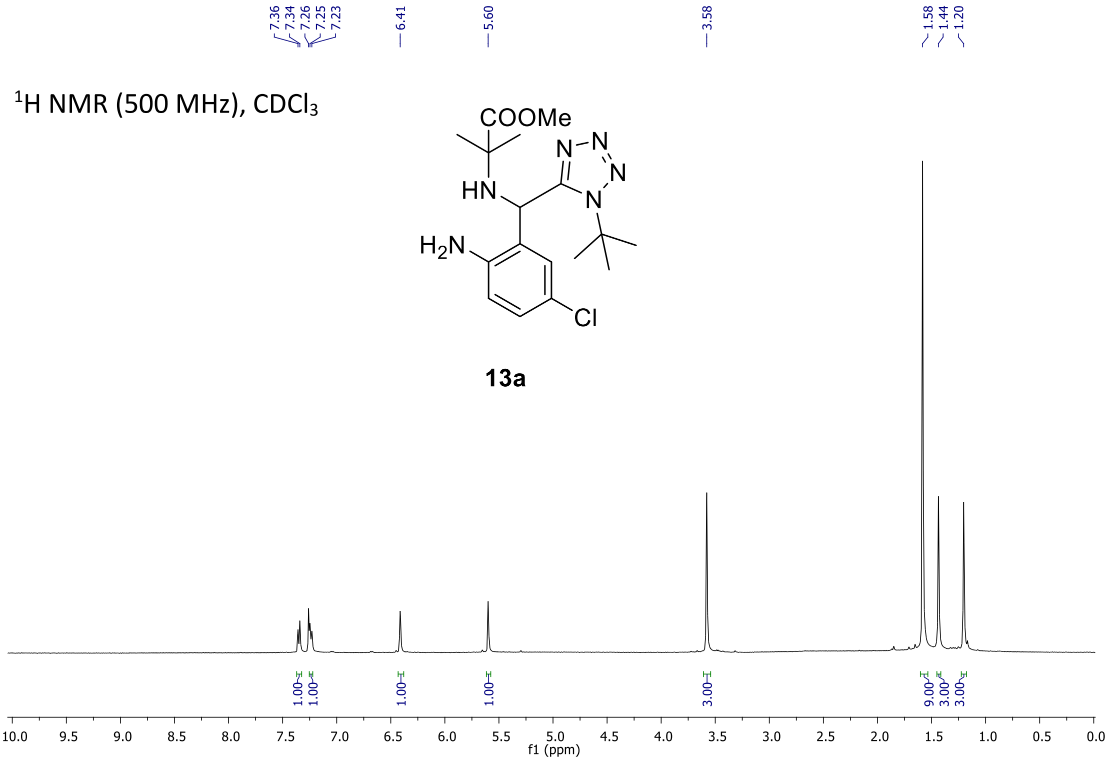

13a

$^{13}C\{H\}$ NMR (125 MHz), $CDCl_3$

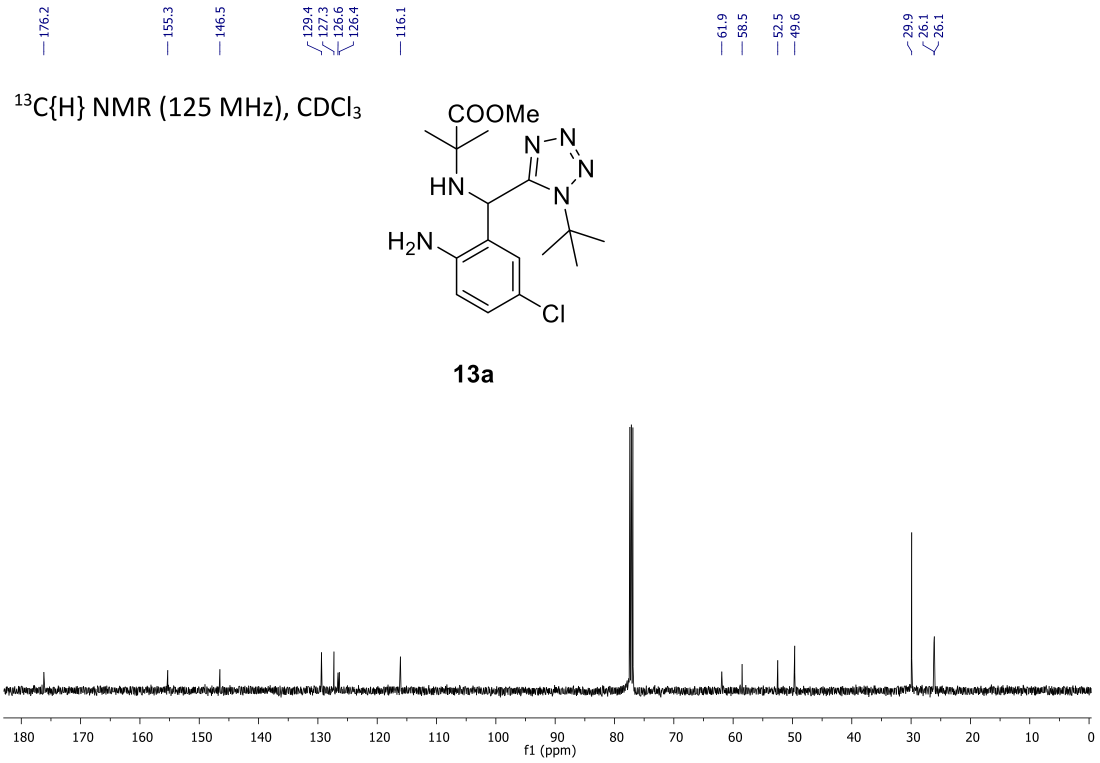

13a

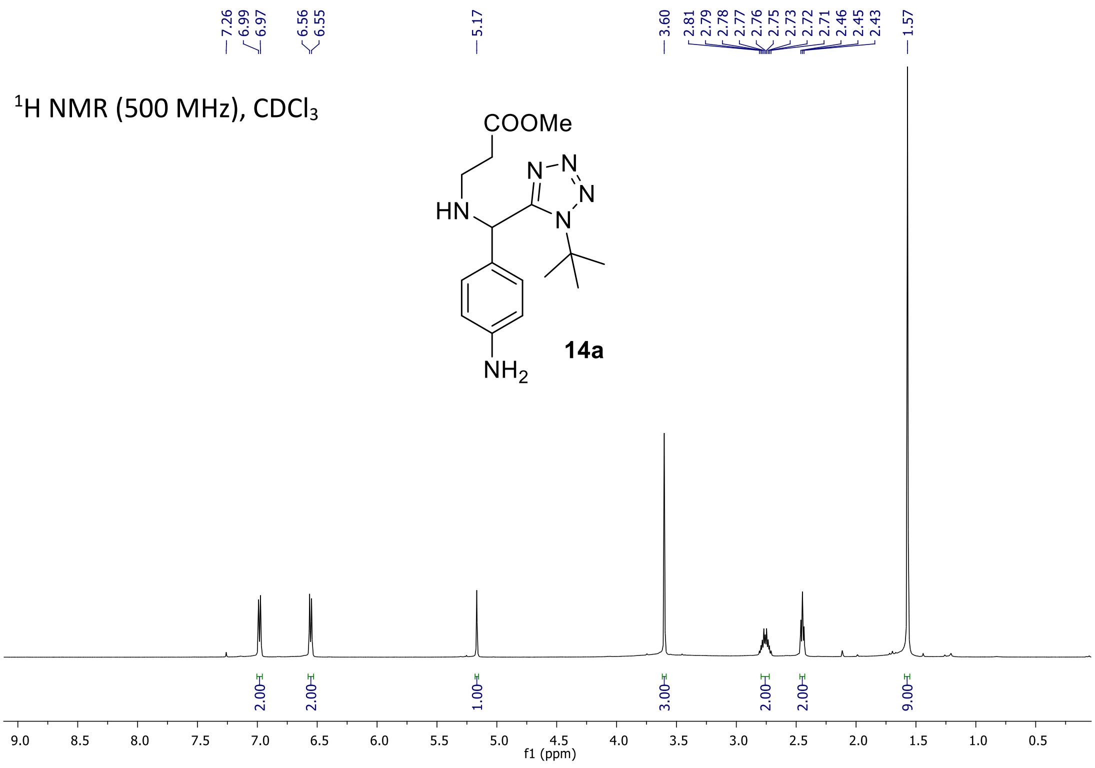

¹H NMR (500 MHz), CDCl₃
COOMe
HN
N
NH₂
14a
7.26
6.99
6.97
6.56
6.55
5.17
3.60
2.81
2.79
2.78
2.77
2.76
2.75
2.73
2.72
2.71
2.46
2.45
2.43
1.57
2.00
2.00
1.00
3.00
2.00
2.00
9.00
f1 (ppm)

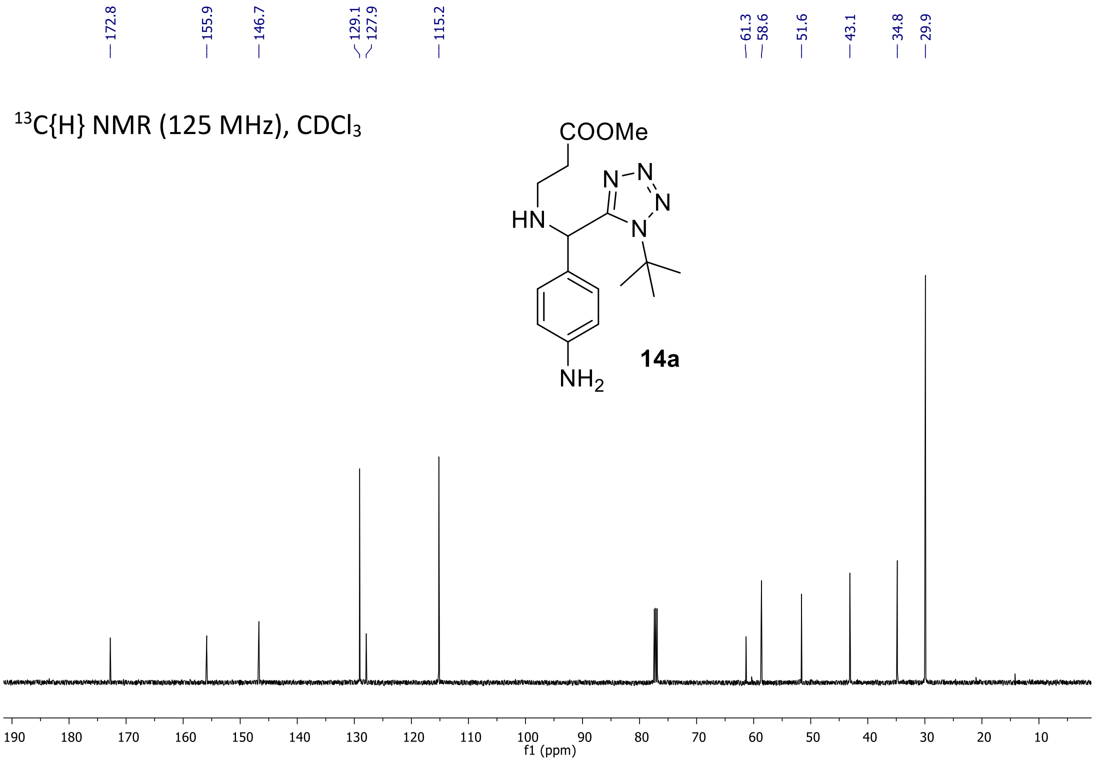

¹³C{H} NMR (125 MHz), CDCl₃
COOMe
HN
N
NH₂
14a
172.8
155.9
146.7
129.1
127.9
115.2
61.3
58.6
51.6
43.1
34.8
29.9
f1 (ppm)

^{1}H NMR (500 MHz), $CDCl_3$

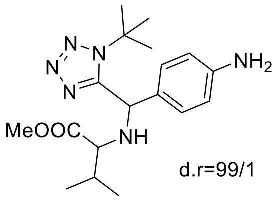

15a

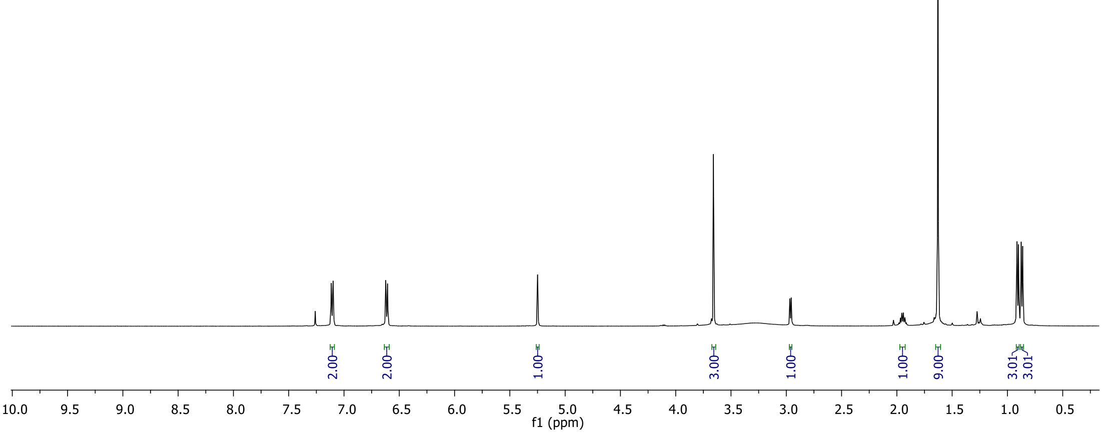

$^{13}C\{H\}$ NMR (125 MHz), $CDCl_3$

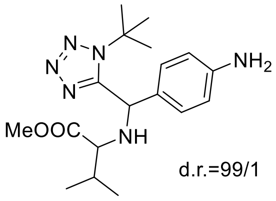

15a

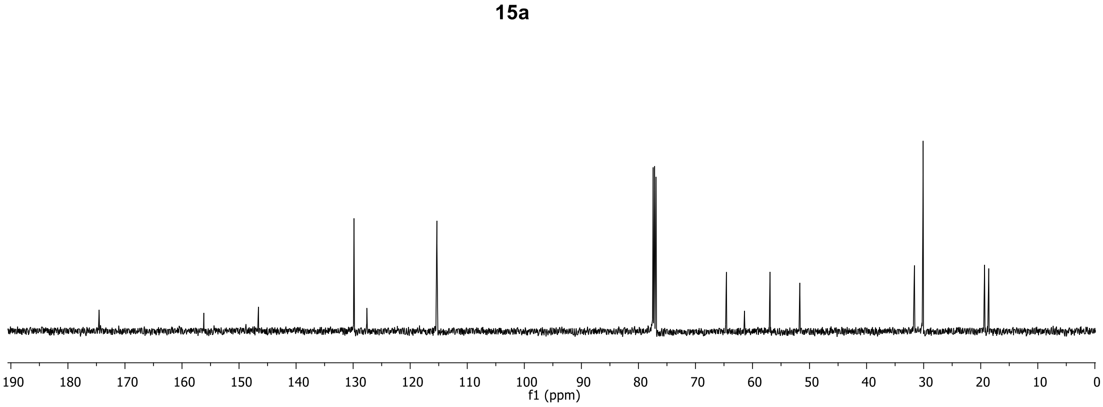

^{1}H NMR (500 MHz), $CDCl_3$

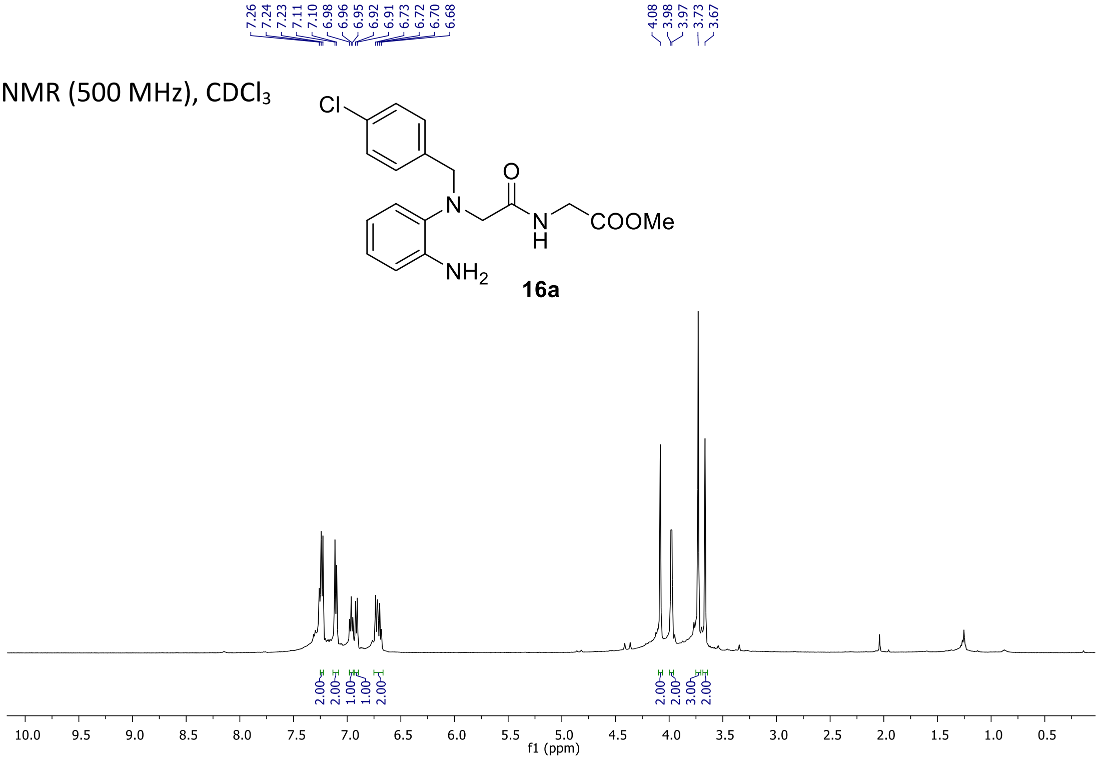

^{13}C{H} NMR (125 MHz), $CDCl_3$

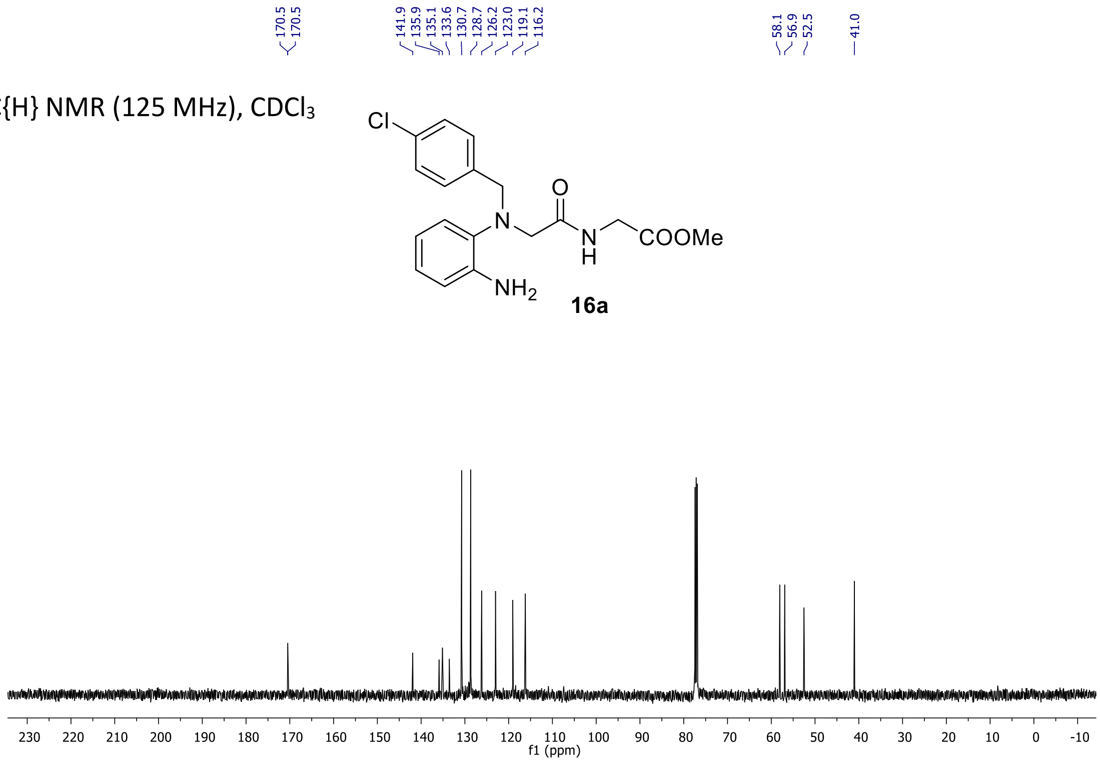

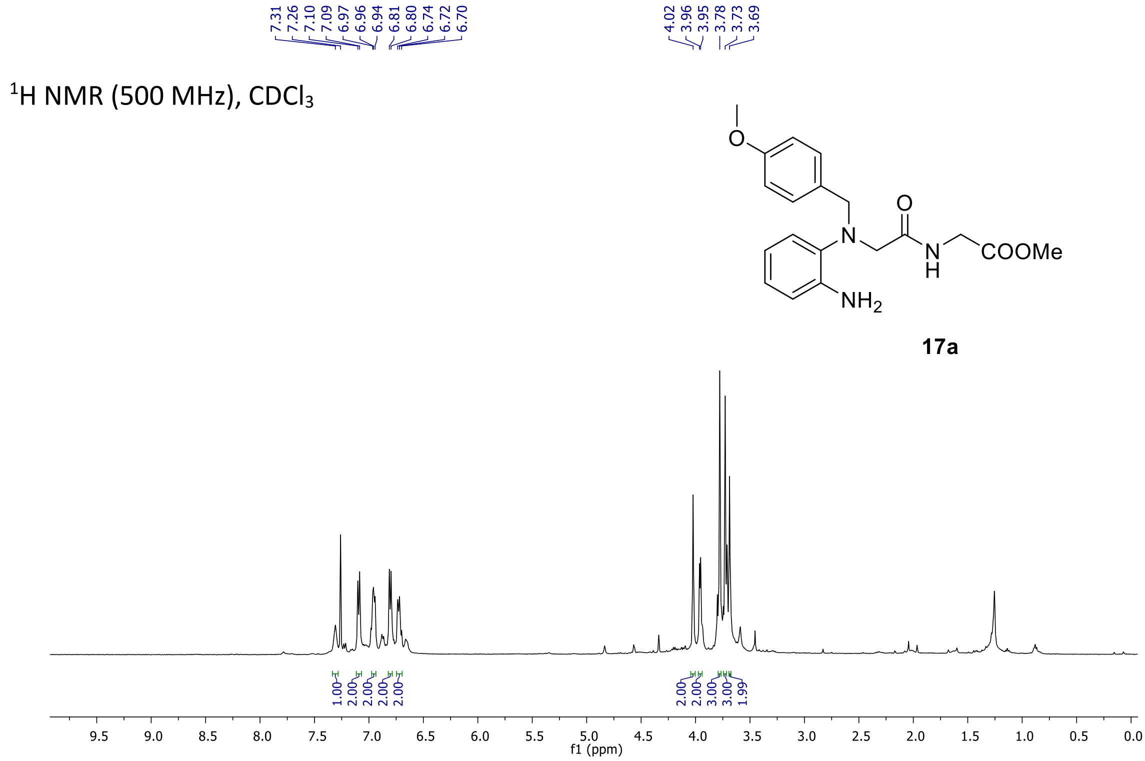
7.31 7.26 7.10 7.09 6.97 6.96 6.94 6.81 6.80 6.74 6.72 6.70
4.02 3.96 3.95 3.78 3.73 3.69
1H NMR (500 MHz), CDCl3
O
N
O
N
H
COOMe
NH2
17a
1.00 2.00 2.00 2.00 2.00
2.00 2.00 3.00 3.00 1.99
9.5 9.0 8.5 8.0 7.5 7.0 6.5 6.0 5.5 5.0 4.5 4.0 3.5 3.0 2.5 2.0 1.5 1.0 0.5 0.0
f1 (ppm)

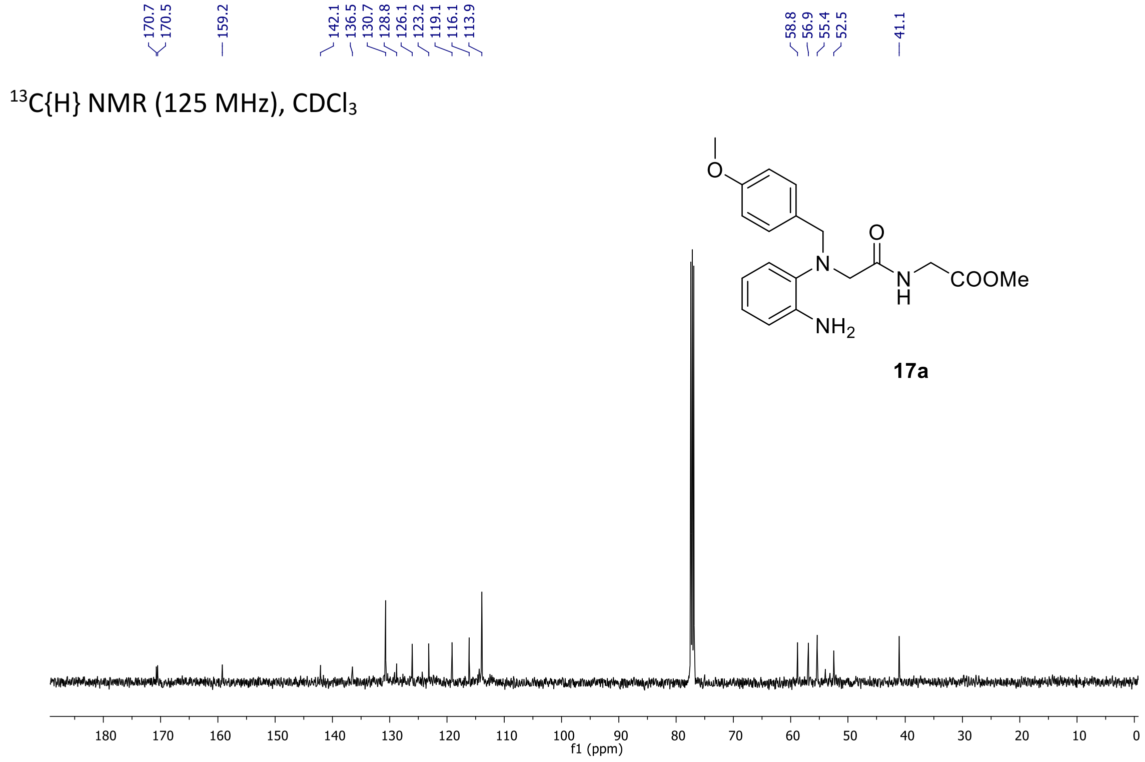
170.7 170.5 159.2 142.1 136.5 130.7 128.8 126.1 123.2 119.1 116.1 113.9
58.8 56.9 55.4 52.5 41.1
13C{H} NMR (125 MHz), CDCl3
O
N
O
N
H
COOMe
NH2
17a
180 170 160 150 140 130 120 110 100 90 80 70 60 50 40 30 20 10 0
f1 (ppm)

^{1}H NMR (500 MHz), CDCl$_3$

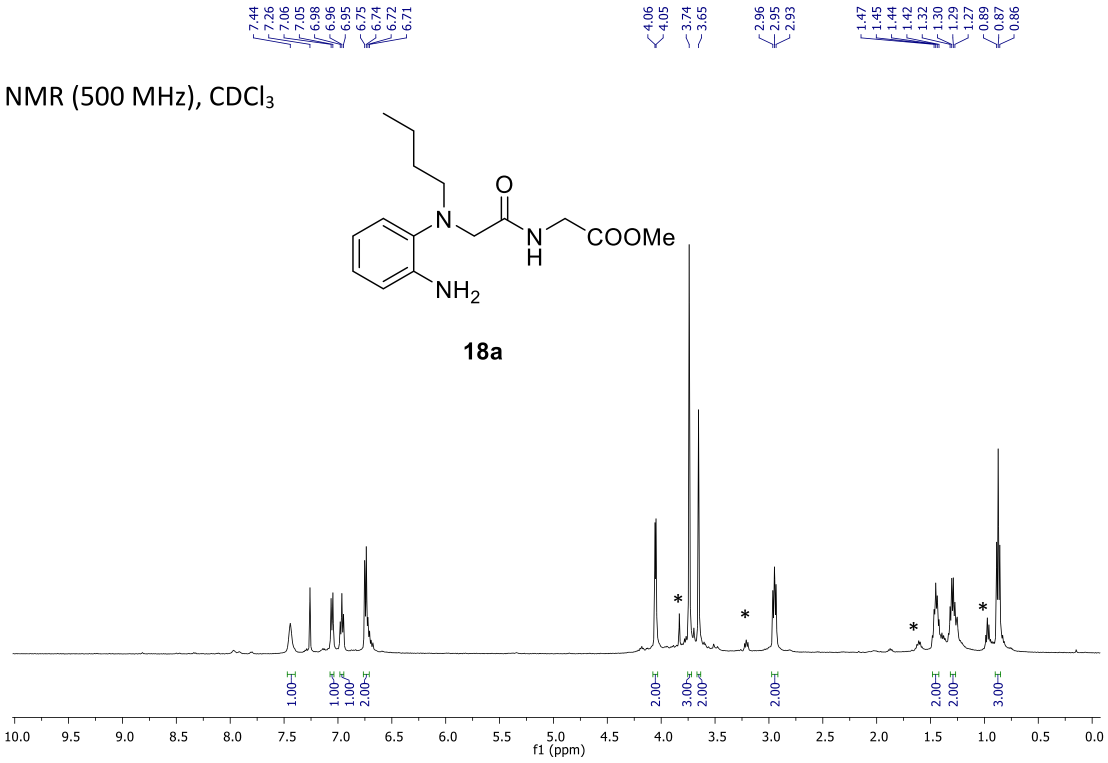

^{13}C{H} NMR (125 MHz), CDCl$_3$

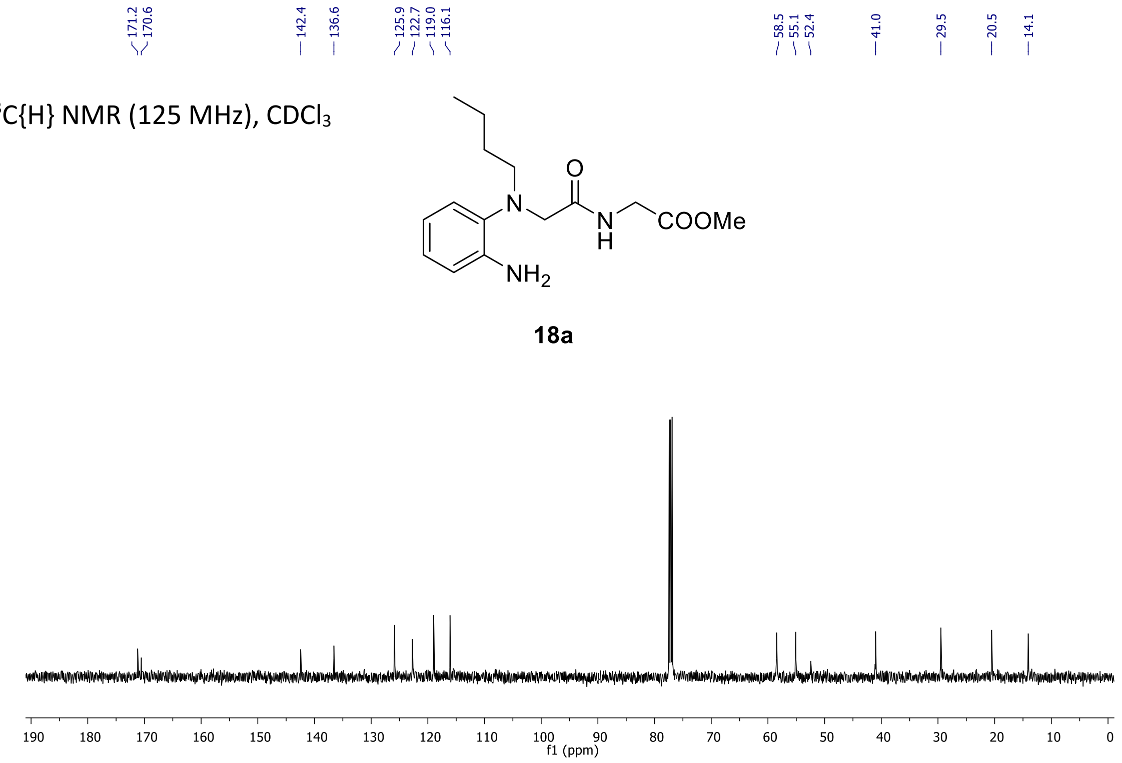

* Small amount of **18b**.

^{1}H NMR (500 MHz), $CDCl_3$

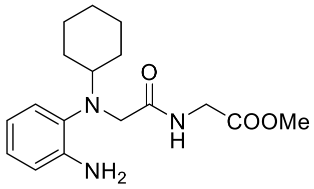

19a

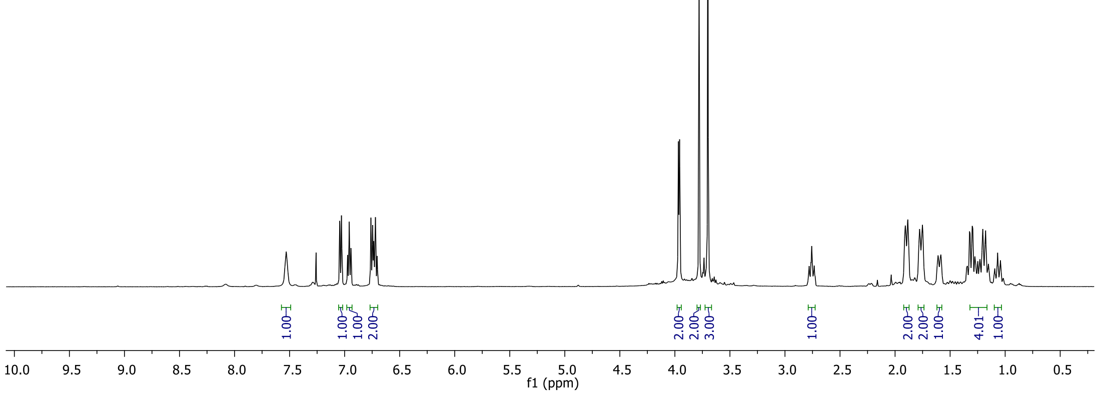

$^{13}C\{H\}$ NMR (125 MHz), $CDCl_3$

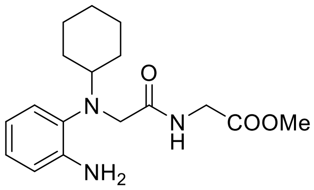

19a

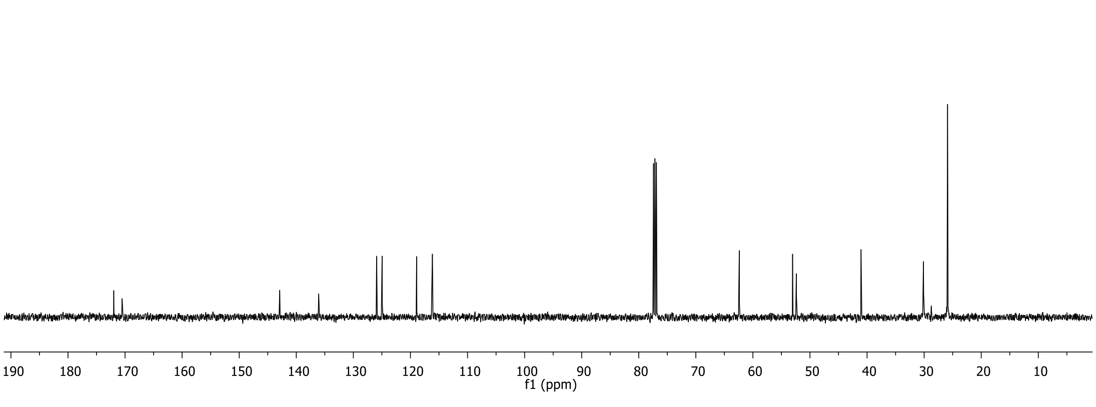

^{1}H NMR (500 MHz), $CDCl_3$

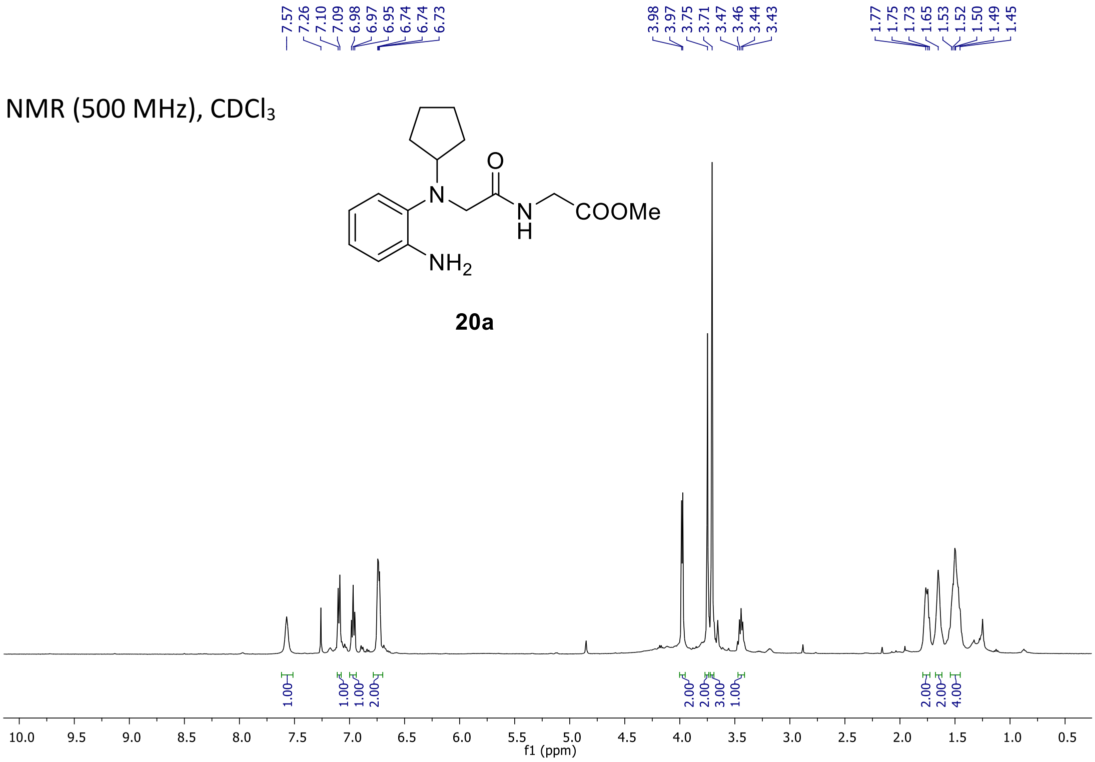

20a

$^{13}C\{H\}$ NMR (125 MHz), $CDCl_3$

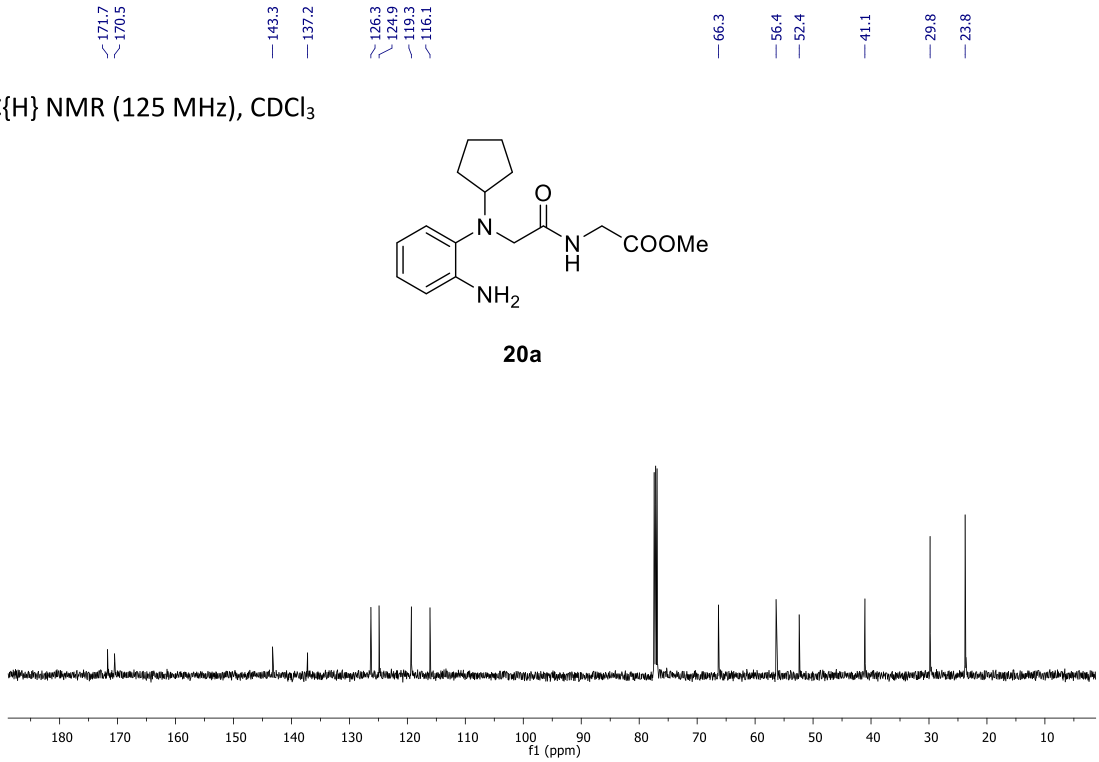

20a

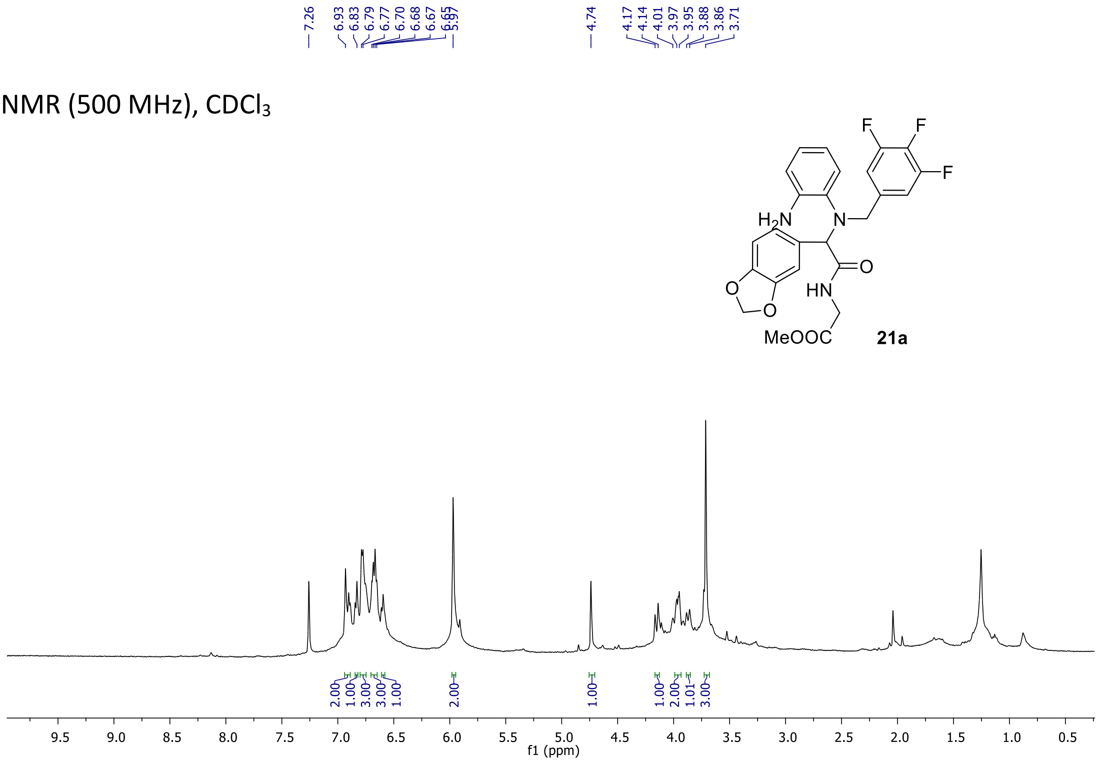
7.26
6.93
6.83
6.79
6.77
6.70
6.68
6.67
6.65
5.97
4.74
4.17
4.14
4.01
3.97
3.95
3.88
3.86
3.71
¹H NMR (500 MHz), CDCl₃
H₂N
N
F
F
F
O
HN
O
O
MeOOC
21a
2.00
1.00
3.00
3.00
1.00
2.00
1.00
1.00
2.00
1.01
3.00
9.5
9.0
8.5
8.0
7.5
7.0
6.5
6.0
5.5
5.0
4.5
4.0
3.5
3.0
2.5
2.0
1.5
1.0
0.5
f1 (ppm)

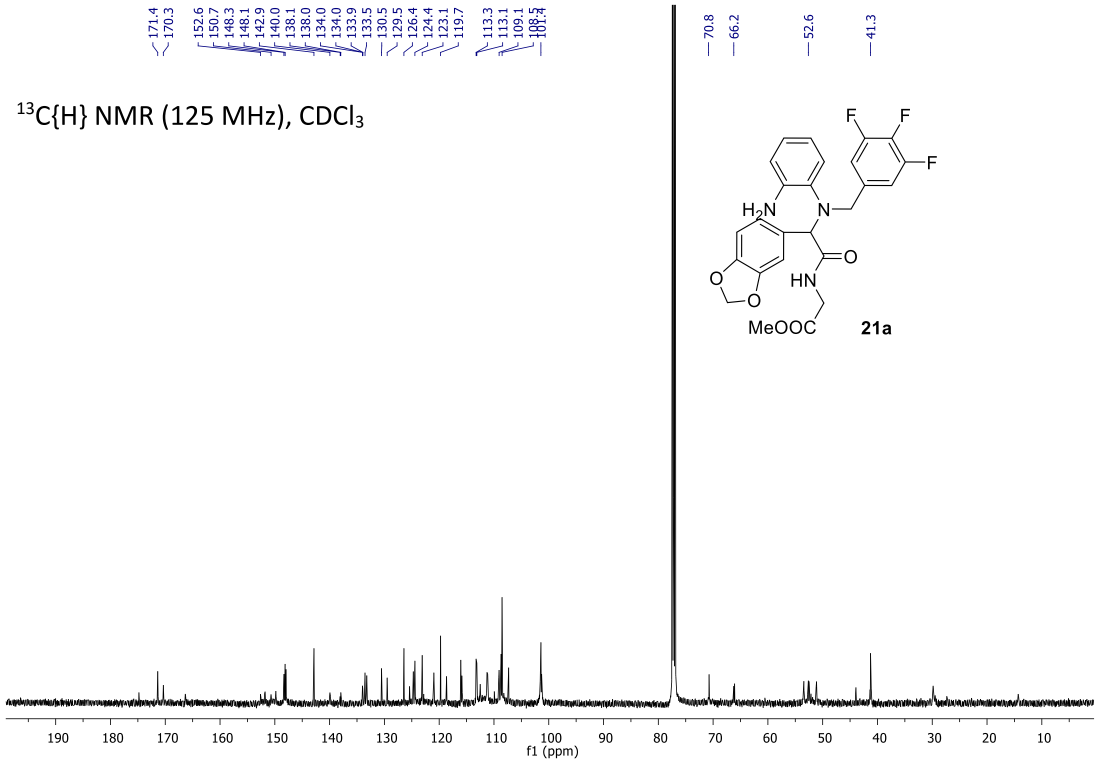
171.4
170.3
152.6
150.7
148.3
148.1
142.9
140.0
138.1
138.0
134.0
134.0
133.9
133.5
130.5
129.5
126.4
124.4
123.1
119.7
113.3
113.1
109.1
108.5
101.4
70.8
66.2
52.6
41.3
¹³C{H} NMR (125 MHz), CDCl₃
H₂N
N
F
F
F
O
HN
O
O
MeOOC
21a
190
180
170
160
150
140
130
120
110
100
90
80
70
60
50
40
30
20
10
f1 (ppm)

Copies of ^{1}H and ^{13}C NMR spectra of dihydroquinoxalinones 16b-21b

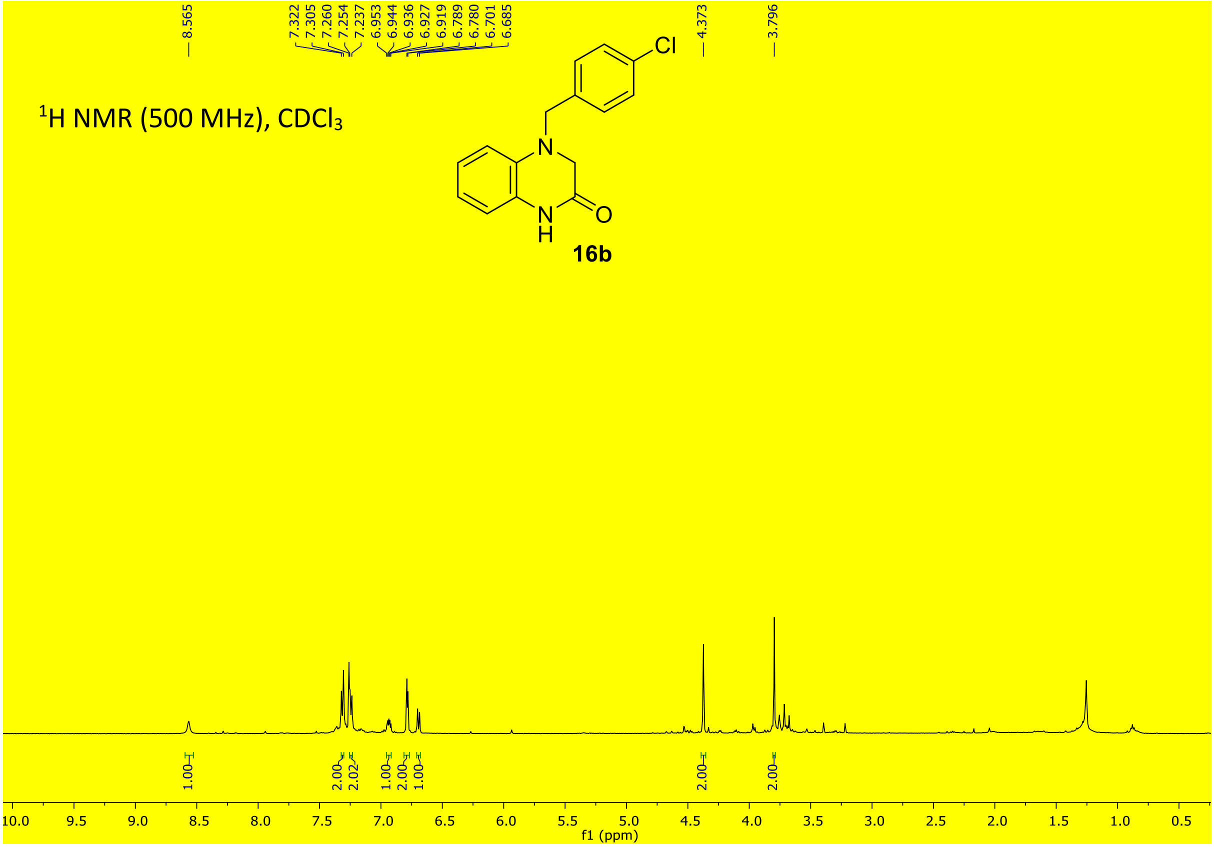

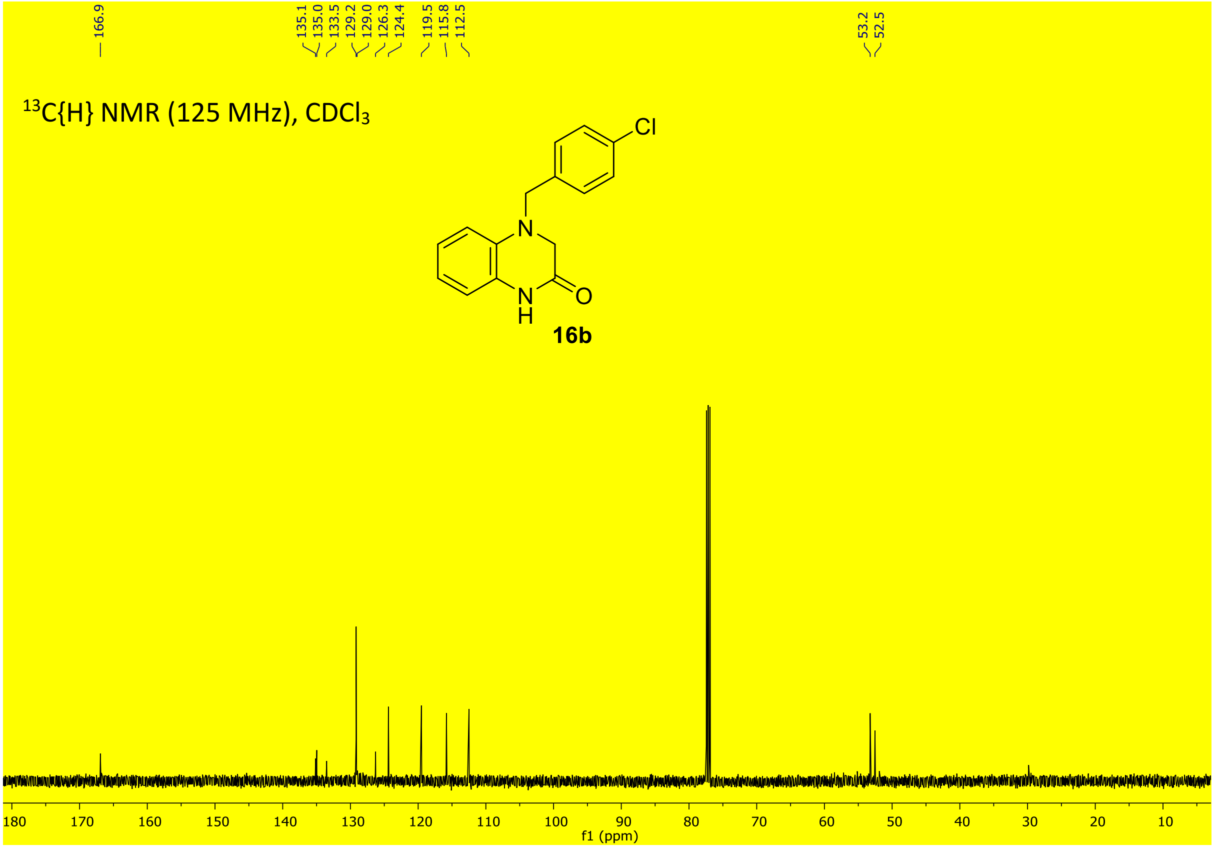

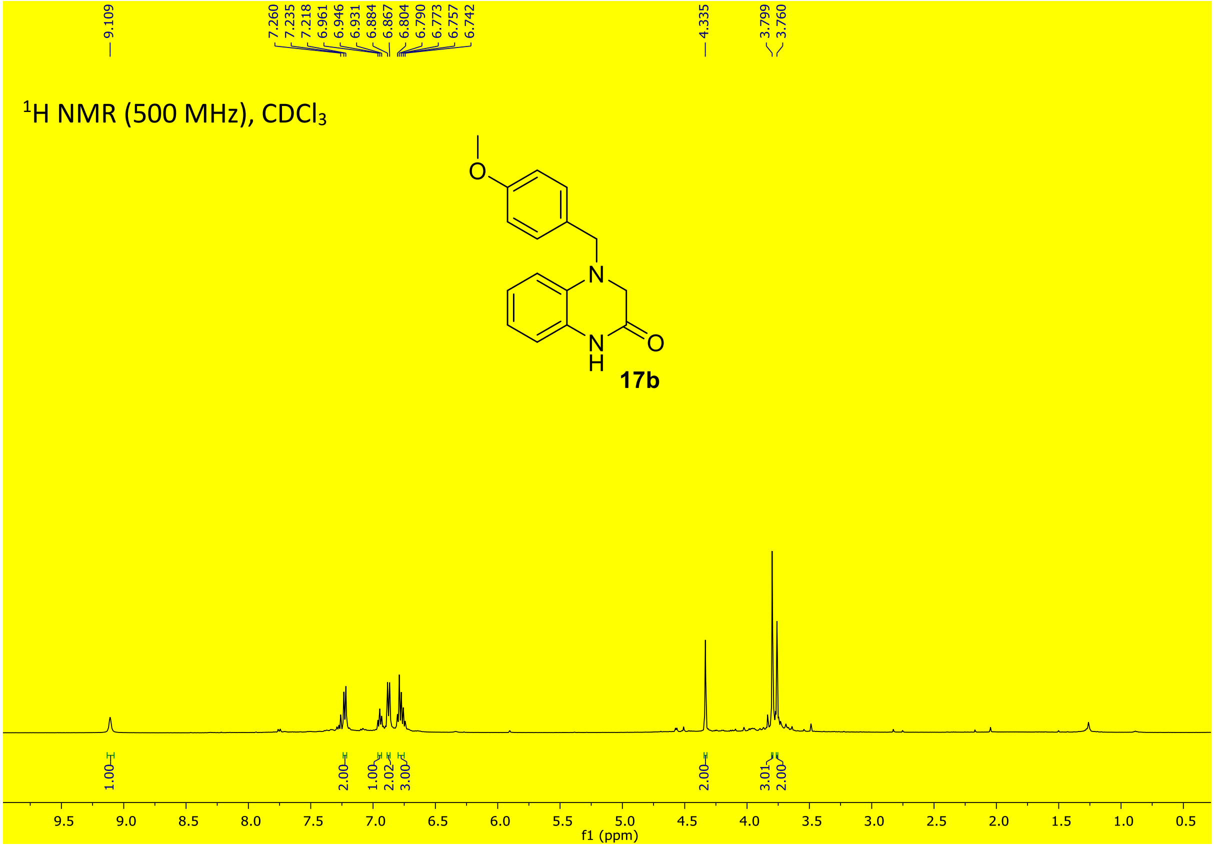

9.109
7.260
7.235
7.218
6.961
6.946
6.931
6.884
6.867
6.804
6.790
6.773
6.757
6.742
4.335
3.799
3.760
^{1}H NMR (500 MHz), $CDCl_3$
O
N
N
H
O
17b
1.00
2.00
1.00
2.02
3.00
2.00
3.01
2.00
9.5 9.0 8.5 8.0 7.5 7.0 6.5 6.0 5.5 5.0 4.5 4.0 3.5 3.0 2.5 2.0 1.5 1.0 0.5
f1 (ppm)

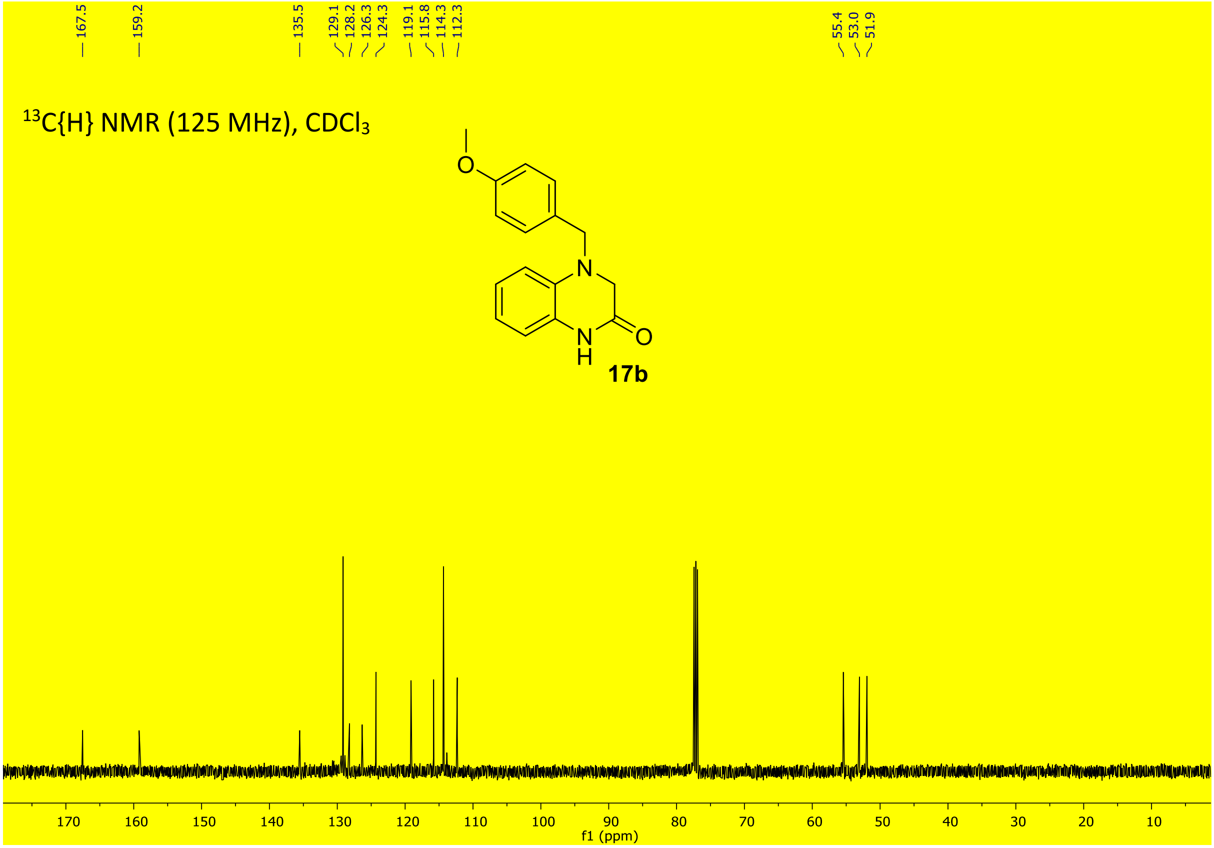

167.5
159.2
135.5
129.1
128.2
126.3
124.3
119.1
115.8
114.3
112.3
55.4
53.0
51.9
^{13}C{H} NMR (125 MHz), $CDCl_3$
O
N
N
H
O
17b
170 160 150 140 130 120 110 100 90 80 70 60 50 40 30 20 10
f1 (ppm)

^1H NMR (500 MHz), $CDCl_3$

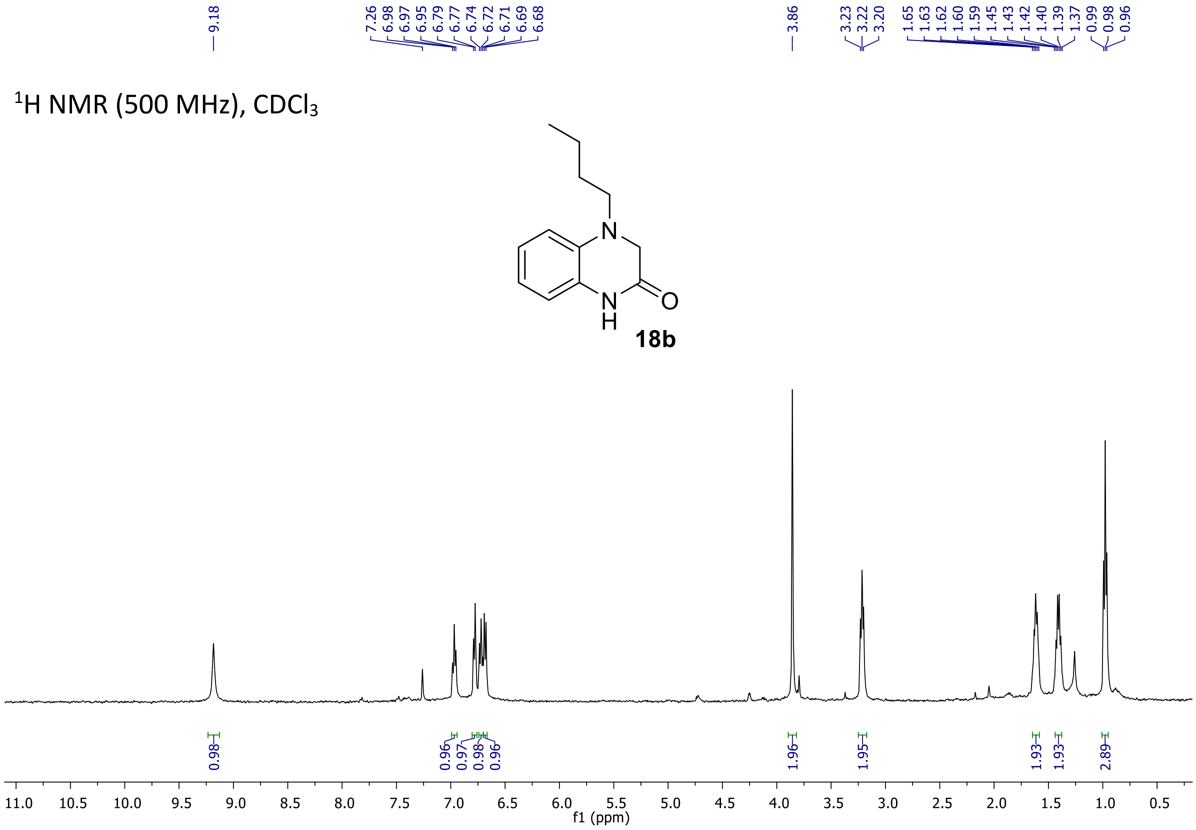

18b

$^{13}C\{H\}$ NMR (125 MHz), $CDCl_3$

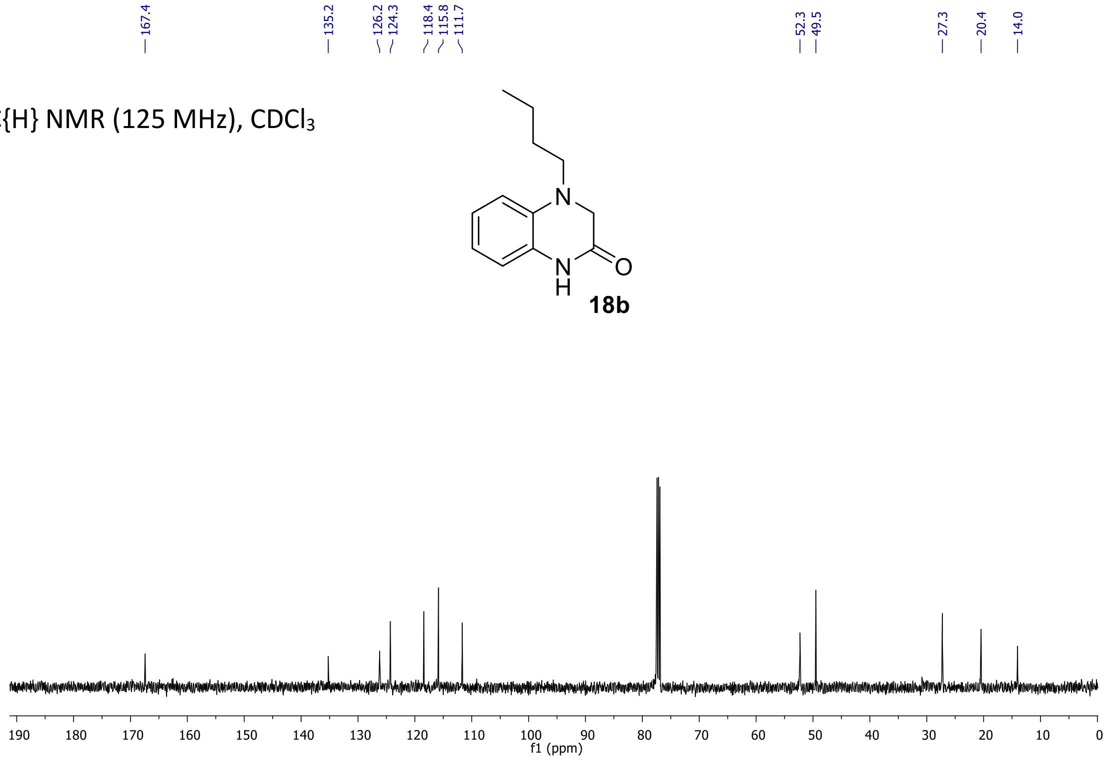

18b

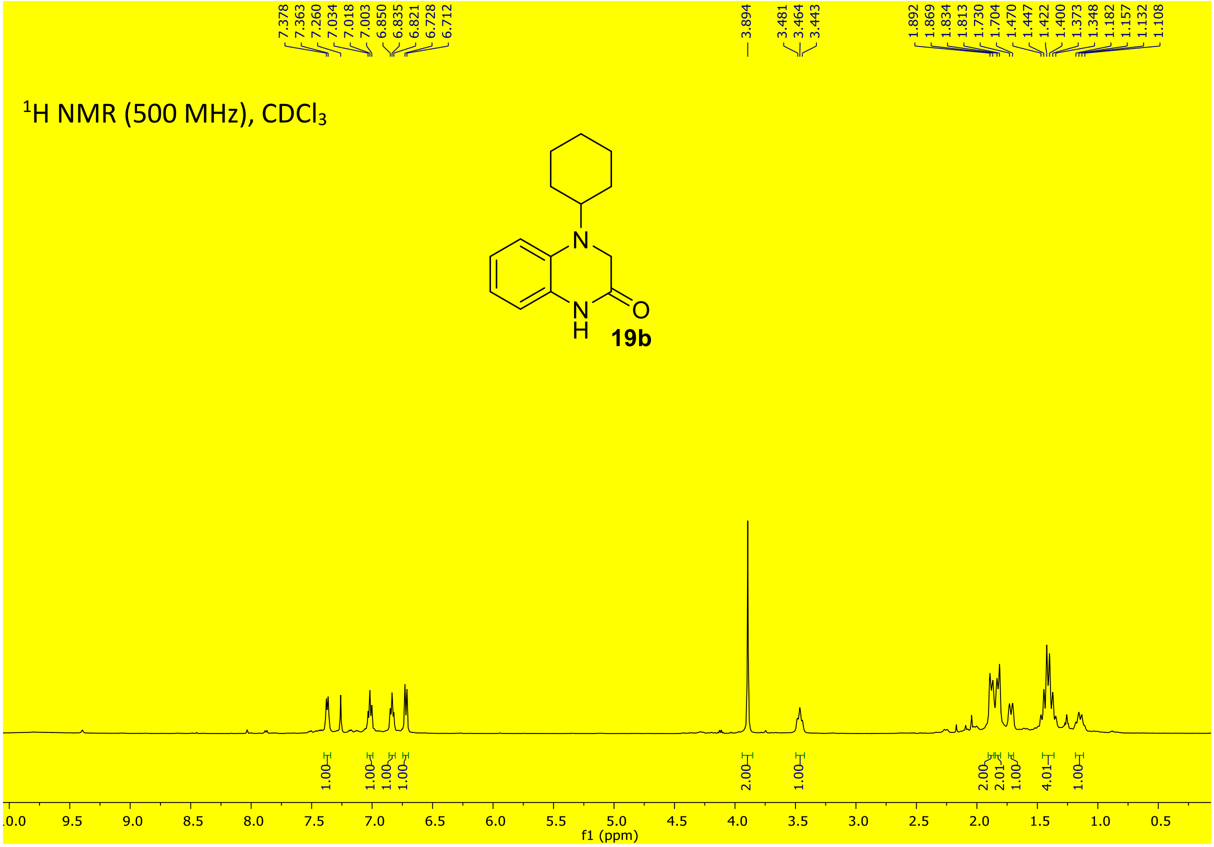
7.378
7.363
7.260
7.034
7.018
7.003
6.850
6.835
6.821
6.728
6.712
3.894
3.481
3.464
3.443
1.892
1.869
1.834
1.813
1.730
1.704
1.470
1.447
1.422
1.400
1.373
1.348
1.182
1.157
1.132
1.108
1H NMR (500 MHz), CDCl3
N
N
H
O
19b
1.00
1.00
1.00
1.00
2.00
1.00
2.00
2.01
1.00
4.01
1.00
f1 (ppm)

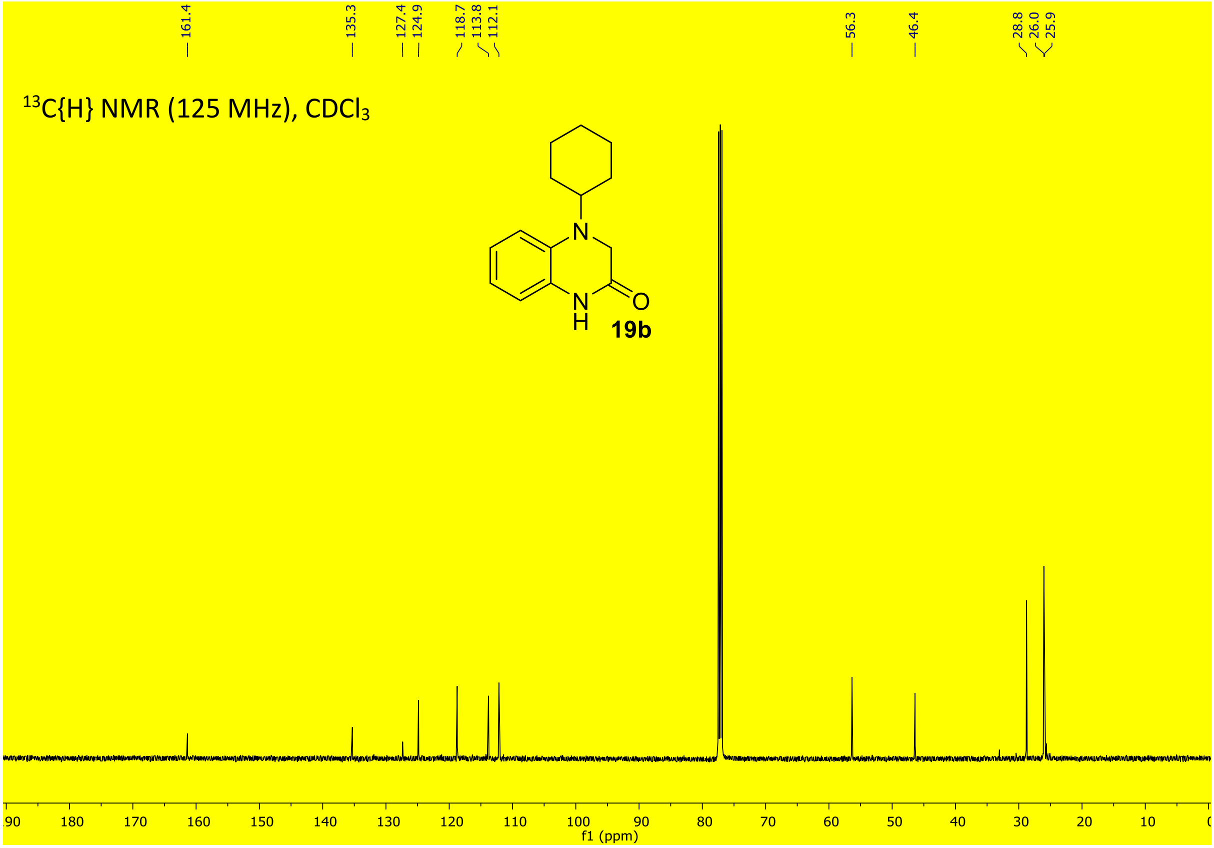
161.4
135.3
127.4
124.9
118.7
113.8
112.1
56.3
46.4
28.8
26.0
25.9
13C{H} NMR (125 MHz), CDCl3
N
N
H
O
19b
f1 (ppm)

^1H NMR (500 MHz), $CDCl_3$

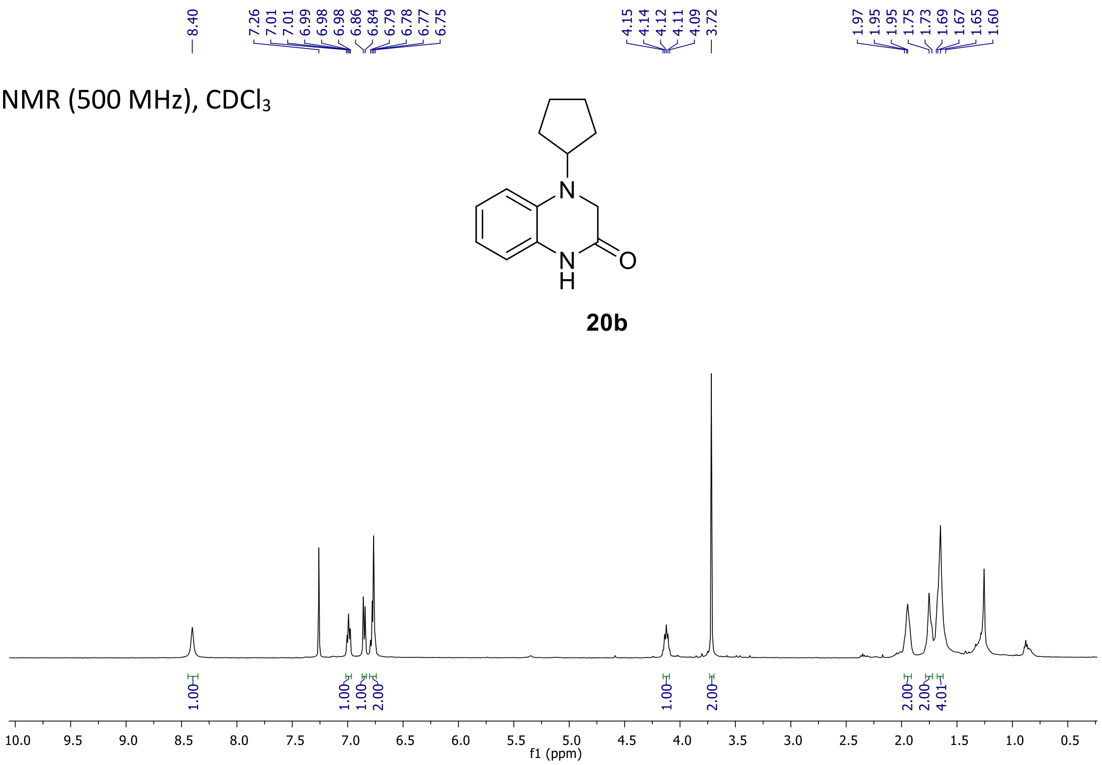

20b

$^{13}C\{H\}$ NMR (125 MHz), $CDCl_3$

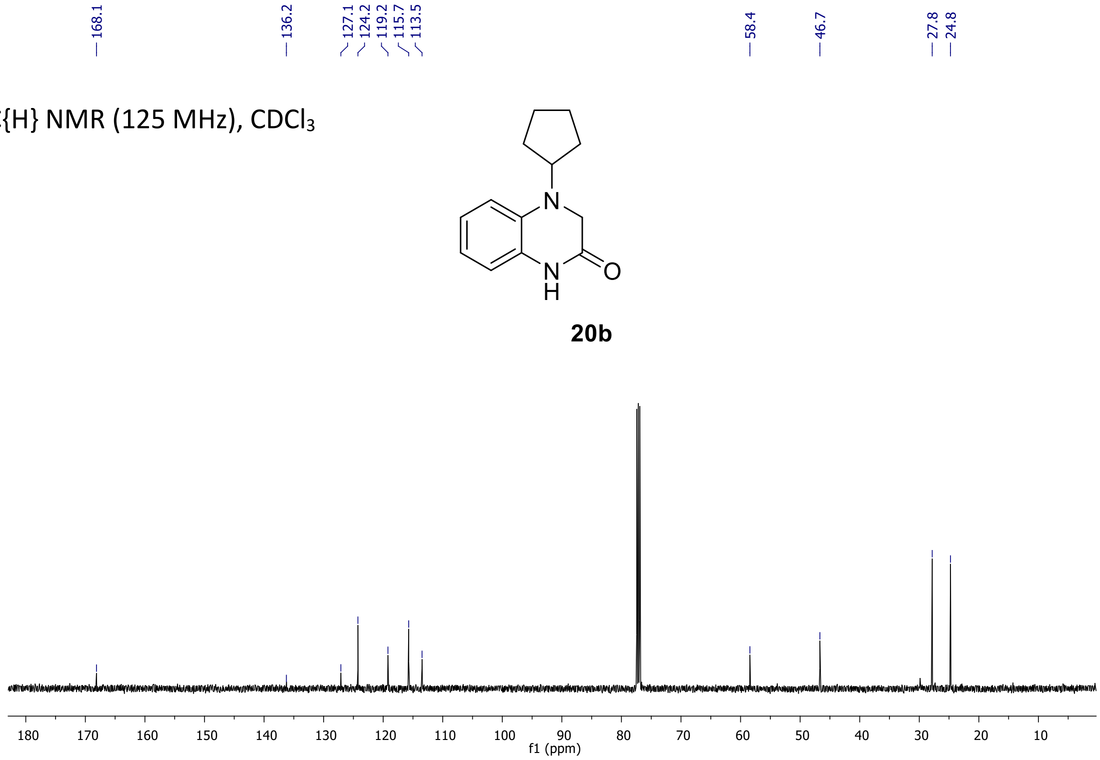

20b

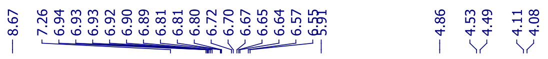

^{1}H NMR (500 MHz), $CDCl_3$

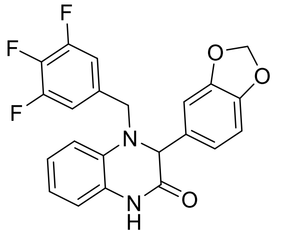

21b

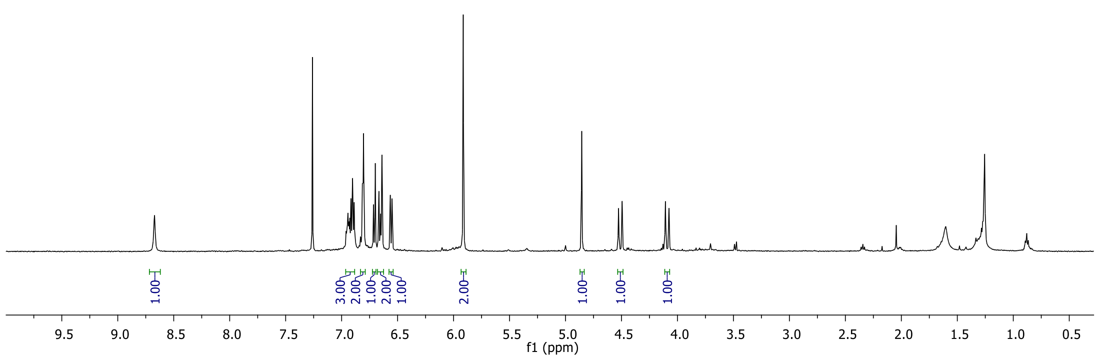

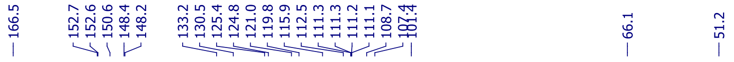

$^{13}C\{H\}$ NMR (125 MHz), $CDCl_3$

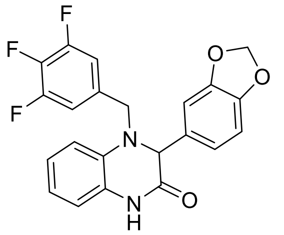

21b

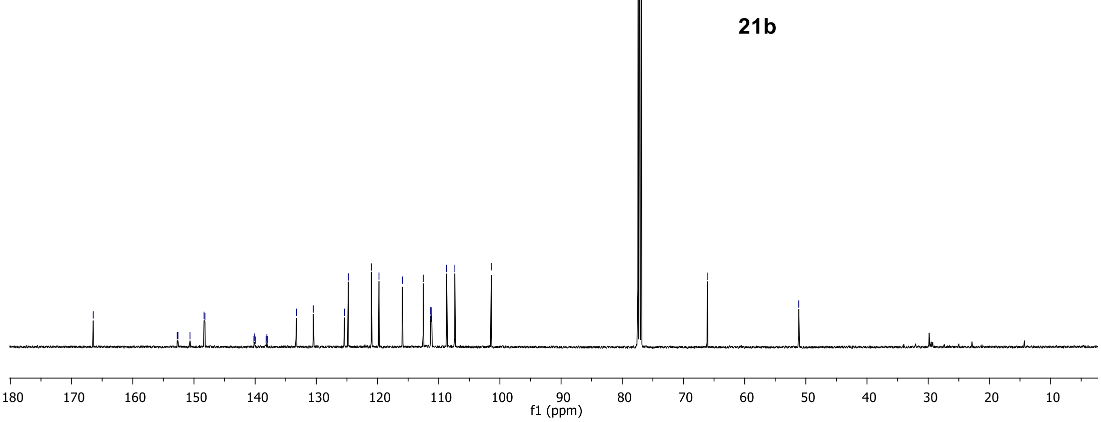

Supplement: Supplementary file 1 [file molecules-27-04395-s001.zip › molecules-1788150-supplementary.pdf]
